# Structure of the endosomal Commander complex linked to Ritscher-Schinzel syndrome

**DOI:** 10.1016/j.cell.2023.04.003

**Published:** 2023-05-11

**Authors:** Michael D. Healy, Kerrie E. McNally, Rebeka Butkovič, Molly Chilton, Kohji Kato, Joanna Sacharz, Calum McConville, Edmund R.R. Moody, Shrestha Shaw, Vicente J. Planelles-Herrero, Sathish K.N. Yadav, Jennifer Ross, Ufuk Borucu, Catherine S. Palmer, Kai-En Chen, Tristan I. Croll, Ryan J. Hall, Nikeisha J. Caruana, Rajesh Ghai, Thi H.D. Nguyen, Kate J. Heesom, Shinji Saitoh, Imre Berger, Christiane Schaffitzel, Tom A. Williams, David A. Stroud, Emmanuel Derivery, Brett M. Collins, Peter J. Cullen

**Affiliations:** 1 Centre for Cell Biology of Chronic Disease, Institute for Molecular Bioscience, The University of Queensland, St. Lucia, QLD 4072, Australia; 2 School of Biochemistry, Biomedical Sciences Building, University of Bristol, BS8 1TD Bristol, UK; 3 MRC Laboratory of Molecular Biology, CB2 0QH Cambridge, UK; 4 Department of Biochemistry and Pharmacology, The Bio21 Molecular Science and Biotechnology Institute, The University of Melbourne, Parkville, VIC, Australia; 5 School of Biological Sciences, University of Bristol, BS8 1TD Bristol, UK; 6 Cambridge Institute for Medical Research, University of Cambridge, CB2 0XY Cambridge, UK; 7 Institute of Health and Sport (iHeS), Victoria University, Melbourne, VIC Australia; 8 Proteomics Facility, School of Biochemistry, Biomedical Sciences Building, University of Bristol, BS8 1TD Bristol, UK; 9 Department of Pediatrics and Neonatology, Nagoya City University Graduate School of Medical Sciences and Medical School, Nagoya, Japan; 10 Max Planck Bristol Centre for Minimal Biology, Department of Chemistry, University of Bristol, BS8 1TS Bristol, UK; 11 Murdoch Children’s Research Institute, Royal Children’s Hospital, Melbourne, VIC Australia

**Keywords:** Retriever, COMMD, Commander, Retromer, Endosome, Ritscher-Schinzel syndrome, AlphaFold, DENND10, VPS29, CCDC22, CCDC93, CCC complex

## Abstract

The Commander complex is required for endosomal recycling of diverse transmembrane cargos and is mutated in Ritscher-Schinzel syndrome. It comprises two sub-assemblies: Retriever composed of VPS35L, VPS26C, and VPS29; and the CCC complex which contains twelve subunits: COMMD1-COMMD10 and the coiled-coil domain-containing (CCDC) proteins CCDC22 and CCDC93. Combining X-ray crystallography, electron cryomicroscopy, and *in silico* predictions, we have assembled a complete structural model of Commander. Retriever is distantly related to the endosomal Retromer complex but has unique features preventing the shared VPS29 subunit from interacting with Retromer-associated factors. The COMMD proteins form a distinctive hetero-decameric ring stabilized by extensive interactions with CCDC22 and CCDC93. These adopt a coiled-coil structure that connects the CCC and Retriever assemblies and recruits a 16th subunit, DENND10, to form the complete Commander complex. The structure allows mapping of disease-causing mutations and reveals the molecular features required for the function of this evolutionarily conserved trafficking machinery.

## Highlights

• CryoEM, crystal and modeled structure of the trimeric Retriever complex
• Crystal and CryoEM structures of the CCC complex
• Model and functional validation of the holo-Commander complex
• Disease-causing mutations in Commander perturb stability and subunit interactions

## Graphical abstract

**Figure:**
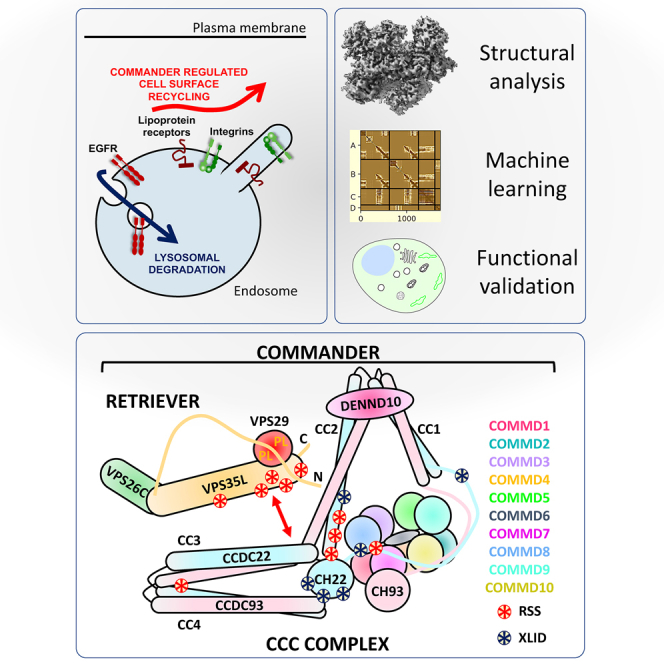

## Teaser

Complete structural model and functional validation of the sixteen-subunit human Commander complex reveals mechanisms of assembly and how mutations causing Ritscher-Schinzel syndrome perturb stability and subunit interactions of this evolutionarily conserved trafficking machinery.

## Introduction

Membrane trafficking through the endosomal network is central to eukaryotic cell biology. Proteins entering the network are sorted between lysosomal degradation or retrieval and recycling to organelles that include the cell surface and the biosynthetic and autophagic compartments.^1^ Several protein machineries are essential for cargo transport, including Retromer and the recently identified Commander complex.^2^^,^^3^^,^^4^^,^^5^ Commander regulates Retromer-independent retrieval and recycling of hundreds of proteins including integrins and lipoprotein receptors,^6^ and mutations in its subunits are causative for X-linked intellectual disability (XLID) and Ritscher-Schinzel syndrome (RSS), a multi-system developmental disorder characterised by abnormal craniofacial features, cerebellar hypoplasia, and stunted cardiovascular development.^7^^,^^8^^,^^9^^,^^10^^,^^11^^,^^12^^,^^13^^,^^14^

**Figure 1:**
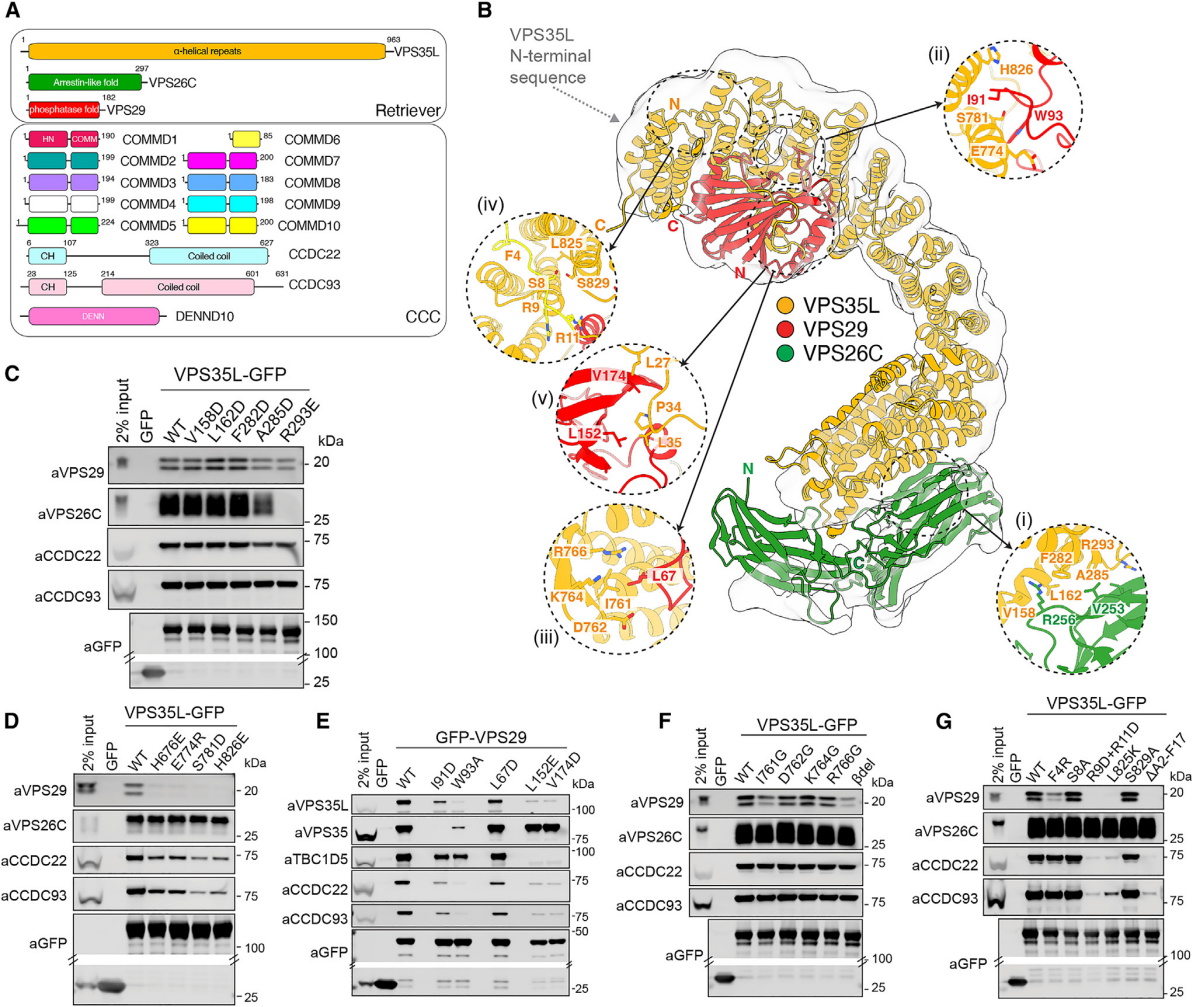
Architecture of the human Retriever complex (A) Schematic of Retriever and CCC sub-complexes that form the Commander assembly. (B) Low resolution cryoEM envelope of human Retriever with docked AlphaFold2 model (Methods S1). Insets show details of: (*i*). VPS35L:VPS26C interface; (*ii*). VPS35L:VPS29 interaction; (*iii*). β-hairpin of VPS35L interacting with VPS29; (*iv*). intramolecular interaction of N terminus of VPS35L with its C terminus; (*v*). PL motifs in the N terminus of VPS35L interacting with the hydrophobic surface of VPS29. (C and D) GFP-nanotrap of GFP-VPS35L mutants targeting the interface with (C) VPS26C and (D) VPS29. (E) GFP-nanotrap of GFP-VPS29 mutants targeting the major interfaces within Retriever. (F) GFP-nanotrap of GFP-VPS35L mutants targeting the β-hairpin. (G) GFP-nanotrap of GFP-VPS35L mutants targeting the N-terminal sequence mediating intramolecular interactions with the VPS35L C terminus. All blots are representative of three independent experiments. Data S1 shows quantified and raw blots (n = 3). See also Figure S1 and Methods S1.

Commander is composed of sixteen subunits, arranged in two sub-assemblies, the CCC and Retriever complexes. Retriever, a VPS26C:VPS35L:VPS29 trimer, shares distant homology to Retromer, itself a trimer of VPS29, VPS35, and either VPS26A or VPS26B (paralogues with VPS26C).^2^^,^^15^ The CCC complex comprises twelve components, the coiled coil domain-containing proteins CCDC22 and CCDC93 and ten COMMD (copper metabolism MURR1 [Mouse U2af1-rs1 region 1) domain) family members COMMD1-COMMD10.^2^^,^^16^^,^^17^^,^^18^^,^^19^ The 16th subunit is DENND10 (differentially expressed in normal and neoplastic cells-containing protein 10, also called FAM45A)^6^^,^^16^^,^^20^^,^^21^^,^^22^ (Figure 1A).

Most transmembrane proteins sorted by Commander, including α5β1 integrin, the amyloid precursor protein (APP), and lipoprotein receptors contain ΦxNxx[YF] sequence motifs (where Φ is a hydrophobic amino acid) that are recruited to Commander via the sorting nexin 17 (SNX17) cargo adaptor.^6^^,^^20^^,^^23^^,^^24^^,^^25^^,^^26^^,^^27^^,^^28^^,^^29^ Mutations in Commander lead to hypercholesterolemia through reduced trafficking of LDLRs.^7^^,^^8^^,^^9^^,^^10^^,^^12^^,^^13^^,^^14^ Commander is also required for cellular infection by human papilloma virus (HPV)^6^ and SARS-CoV-2.^30^^,^^31^^,^^32^^,^^33^ In addition, early studies of individual Commander subunits (COMMD1 and COMMD7) implicated these proteins in regulating NF-κB levels and transcriptional pathways through interactions with cullin-containing ubiquitin ligases.^12^^,^^34^^,^^35^^,^^36^^,^^37^^,^^38^^,^^39^^,^^40^

The COMMD proteins possess a C-terminal COMM domain approximately 70–80 residues in length, as well as an α-helical N-terminal (HN) domain.^41^^,^^42^ The HN domain, while relatively divergent in sequence, has a conserved globular structure of six α-helices α1-α6.^42^^,^^43^ The COMM domain has high sequence similarity across the ten proteins and is composed of three anti-parallel β-strands, β1-β3, and a C-terminal α-helix α7.^42^ The structure of the COMM domain requires it to form obligate dimers, where the β-strands and α-helix of two monomers are tightly interlocked in a “left-handed handshake” topology that buries otherwise solvent exposed hydrophobic sidechains.^42^ Apart from COMMD9^42^ and VPS29,^44^^,^^45^^,^^46^^,^^47^^,^^48^^,^^49^^,^^50^^,^^51^ the structure of Commander is almost completely uncharacterized, and the stoichiometry of the different subunits remain unclear.

Here we present a complete model of the sixteen subunit Commander complex. We show that Retriever, despite a superficial similarity to the distantly related Retromer, forms a heterotrimer with unique features that mediate its divergent function. The ten COMMD proteins assemble into a remarkable and unique structure, forming a hetero-decameric ring from five specific heterodimers, with a precise and evolutionarily conserved subunit organization. The CCDC22 and CCDC93 proteins stabilize the CCC complex, with natively unstructured N-terminal sequences forming extensive interactions around the decameric COMMD ring. An overall model of the fully assembled complex shows how CCDC proteins link the COMMD decameric ring to Retriever and recruit DENND10 through a central coiled-coil structure. Finally, our work allowed us to structurally map all known missense mutations that cause XLID and RSS. Many of these are found near interfaces between subunits, and our unbiased proteomic studies confirm that they perturb complex formation. These studies provide key insights into the assembly and function of the Commander complex required for endosomal recycling of many essential transmembrane cargos.

## Results

### Structure of the trimeric Retriever complex

Recombinant human Retriever (3xStrepII-VPS26C, VPS35L, and VPS29-6xHis) was expressed in insect cells using the biGBac system/MultiBac BEVS^52^^,^^53^ and isolated using affinity purification and size-exclusion chromatography (Methods S1).^6^ The resultant peak contained VPS26C, VPS35L, and VPS29 in a stable 1:1:1 heterotrimer (Methods S1). Dispersed particles with an elongated “footprint”-like morphology were observed in negative stain EM (Methods S1), and single particle 2D/3D cryo-EM classes were dominated by the front view of the “footprint,” with limited other orientations (Methods S1). Due to preferential orientation of the particles, gold-standard Fourier shell correlations (FSCs) are overestimated, and the 3D reconstruction was insufficient for *ab initio* model building (Table S1). However, a high confidence AlphaFold2^54^^,^^55^^,^^56^ model of Retriever aligned well with the low-resolution 3D cryo-EM envelope (Figure 1B; Methods S1; Video S1).

Video S1. Structure of Retriever determined by cryoEM and AlphaFold2 modeling, related to Figure 1

**Figure S1:**
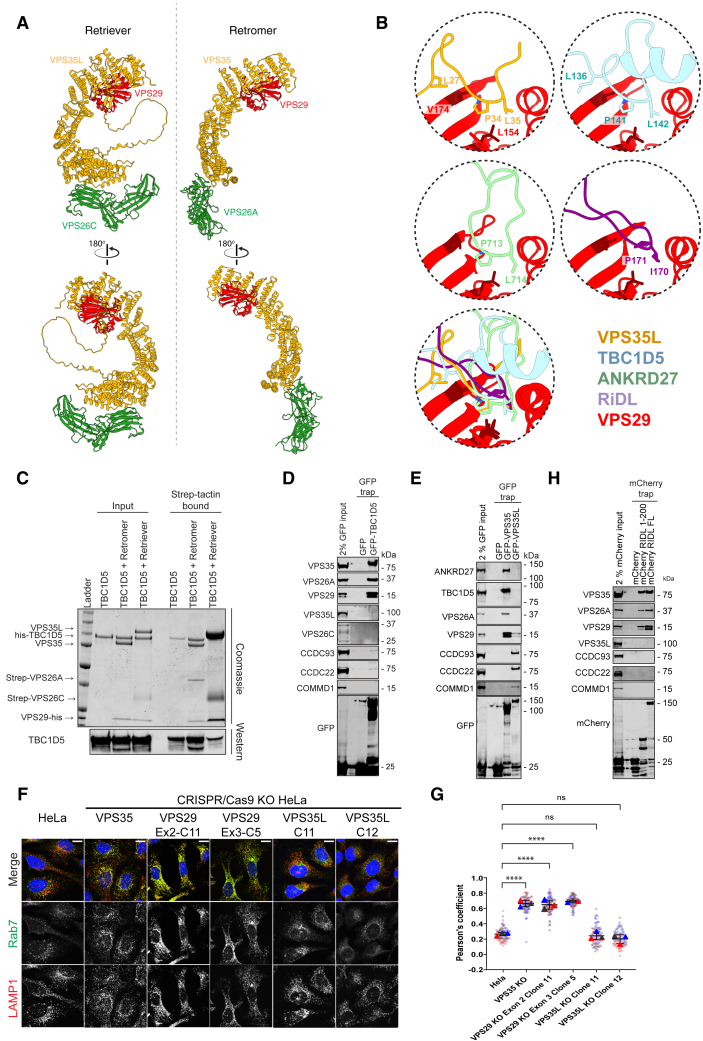
Comparative architecture of Retriever and Retromer assembly and context specific role of VPS29 in accessory protein binding, related to Figures 1 and 2 (A) Comparison between Retriever and Retromer assemblies. (B) VPS35L PL motif binding to VPS29 mimics association of Retromer accessory proteins, TBC1D5 (5GTU) and ANKRD27 (6TL0), and the *Legionella* effector RidL (5WYH) to VPS29. (C) Recombinant Strep-tagged VPS26A-Retromer and Strep-tagged VPS26C-Retriever were incubated with recombinant his-tagged TBC1D5 and subjected to Strep-tactin affinity isolation. Coomassie staining and Western analysis reveals robust association with Retromer but limited association with Retriever. Representative of two independent experiments. (D, E, and H) HEK293T cells were transfected with GFP and (D) GFP-TBC1D5, (E) GFP-VPS35 and GFP-VPS35L, and (H) mCherry-RidL (1–200) or full length (FL) RidL and subjected to GFP- or mCherry-nanotrap. Representative of three independent experiments. (F) VPS35L KO cells do not have elevated lysosomal RAB7 levels. HeLa WT or HeLa KO cells were imaged by confocal microscopy. Scale bars represent 10 μm. Representative images from 3 independent experiments. (G) Quantification of Pearson’s coefficients between RAB7 and LAMP1 from (F). For each condition, 30 cells were quantified per 3 independent experiments (90 cells total). Pearson’s coefficients for individual cells are represented by transparent circles, colored according to the independent experiment. Error bars represent the mean, S.D. Mean represented by solid triangles, colored by replicate. Normality of data was checked prior to one-way ANOVA followed by Dunnett test for multiple comparisons. ^∗∗∗∗^ = p < 0.0001, ns = not significant.

Analogous to Retromer,^57^^,^^58^^,^^59^^,^^60^^,^^61^^,^^62^ VPS35L is an extended α-solenoid that binds VPS26C and VPS29 at its amino- and carboxy-terminal ends respectively (Figures 1B and S1A). VPS35L has little sequence similarity to the Retromer subunit VPS35 (<21% identity), but both are comprised of sixteen HEAT-like α-helical repeat structures. Unlike VPS35, VPS35L has an additional conserved N-terminal sequence (∼180 residues) that is mostly unstructured apart from elements that are predicted to engage both the last three α-helical repeats of VPS35L as well as VPS29 (discussed below). The major interactions within Retriever were validated by structure-based mutagenesis. Analogous to VPS26A/B binding to VPS35,^58^^,^^60^^,^^61^ VPS26C associates with the second and third α-helical repeats in VPS35L via its C-terminal β-sandwich subdomain (Figure 1C). VPS35L(R293E) (see inset *(i)* in Figure 1B) induced a >95% decrease in VPS26C binding but retained binding to VPS29 and the CCC complex (Figure 1C). The major interaction of VPS29 with VPS35L is supported by the carboxy-terminal region of the VPS35L α-solenoid partially wrapping around VPS29, similar to VPS35 in Retromer.^57^ Mutagenesis of key binding residues, VPS35L(H826E) and VPS35L(S781D) (see inset *(ii)* in Figure 1B), resulted in >95% loss of VPS29 binding (Figure 1D), and reciprocal mutations VPS29(I91D) and VPS29(W93A) also decreased VPS35L binding (Figure 1E). Interestingly, reduced VPS29 interaction correlated with a decrease in CCC complex association, suggesting that its binding to VPS35L is important to stabilize Retriever and CCC assembly.

### N-terminal VPS35L sequences bind a conserved VPS29 site to prevent interaction with Retromer-accessory proteins

Two interactions unique to Retriever further promote VPS29 binding and regulate its function. Firstly, a β-sheet extension at the base of VPS35L contacts VPS29 (see inset *(iii)* in Figure 1B). Mutation of this interface with VPS35L(I761G) or complete deletion induced >50% reduction in VPS29 interaction (with CCC complex retained) (Figure 1F), while reciprocal mutant VPS29(L67D) reduced binding to VPS35L by approximately 30% (Figure 1E). The second unique VPS29 interaction involves the first ∼40 residues of the extended N-terminal region of VPS35L (see inset *(iv)* in Figure 1B). The first 17 residues form an intramolecular interaction with the carboxy-terminal region of the VPS35L α-solenoid (Figure 1B), with the same structure predicted for all VPS35L orthologues across species (not shown). Deleting these residues leads to a near complete loss of VPS29 *and* CCC complex binding without affecting VPS26C association, which is replicated with VPS35L(R9D/R11D) and VPS35L(L825K) mutants (Figure 1G).

**Figure 2:**
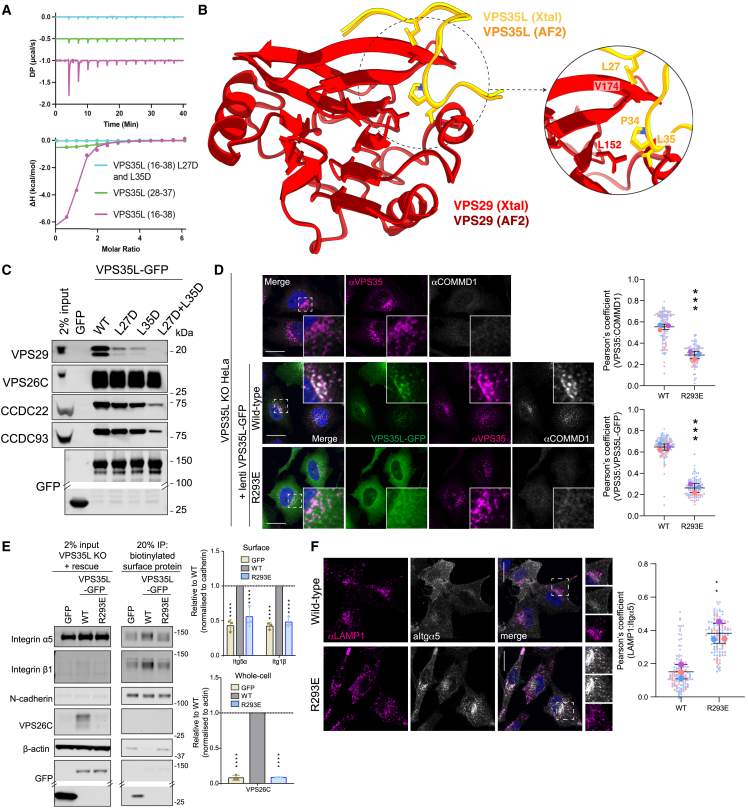
A unique structure in VPS35L regulates VPS29 interaction (A) VPS35L peptides were titrated into VPS29 and binding affinity measured by ITC. *Top* shows the raw data and *bottom* shows the integrated and normalized data fitted with a 1:1 binding model. VPS35L (16-38) had a slightly higher affinity (1.87μM ± 0.8 μM) than VPS35L (28-37) (6.8μM ± 1 μM), while the L27D/L35D mutant peptide showed no binding. *K*_d_ values and standard error of the mean (SEM) are calculated from n = 3. (B) A 1.35-Å crystal structure of VPS29 bound to VPS35L (16-38) confirms the binding of the core ^34^PL^35^ motif to VPS29 and extended interaction of adjacent residues predicted by AlphaFold2. (C) GFP-nanotrap of GFP-VPS35L mutants in the ^26^PL^27^ and ^34^PL^35^ sequences. Data S1 shows quantified and raw blots (n = 3). (D–F) Expression of VPS35L(R293E) in a VPS35L knock-out HeLa cells fails to: (D) rescue the localization of VPS35L or the CCC complex to Retromer-decorated endosomes as observed with wild-type VPS35L; (E) the expression and stability of VPS26C and the steady-state cell surface level of α5β1-integrin; (F) the trafficking of α5-integrin away from LAMP1-positive late endosomes/lysosomes. Data S1 shows quantified band intensities and raw blots. (D and F) Pearson’s coefficients were quantified from >30 cells per 3 independent experiments. Pearson’s coefficients for individual cells and means are presented by smaller and larger circles, respectively, colored according to the independent experiment. The means (n = 3) were compared using a two-tailed unpaired t test. Error bars represent the mean, S.D. ^∗^p < 0.05, ^∗∗^p < 0.01, ^∗∗∗^p < 0.001, ^∗∗∗∗^p < 0.0001. See also Figure S1.

The intramolecular association of VPS35L N- and C-terminal regions serves to tether and orient two Pro-Leu (PL) motifs, ^26^PL^27^ and ^34^PL^35^ of VPS35L, for binding to a hydrophobic surface on VPS29 (see inset *(v)* in Figure 1B). A synthetic VPS35L peptide (Glu16-Ile38) representing the predicted VPS29-binding sequence showed modest affinity for recombinant VPS29 (*K*_d_ of 1.8 ± 0.8 μM) (Figure 2A). An X-ray crystal structure of the VPS29-peptide complex unambiguously confirmed that Leu27 to Leu35 of VPS35L interact with two hydrophobic pockets on VPS29 (Figure 2B; Table S2). The conserved ^34^PL^35^ side chains of VPS35L bind the VPS29 pocket defined by Val174 and Leu152 respectively, VPS35L(L27D) and VPS35L(L35D) mutations block peptide binding by ITC (Figure 2A), and immunoprecipitations showed reduced binding to VPS29 and the CCC complex (Figure 2C). Moreover, VPS29(L152E) and VPS29(V174D) retained Retromer association but displayed a >95% decrease in binding to VPS35L (and CCDC proteins), confirming the central importance of the ^34^PL^35^-VPS29 association for Retriever assembly (Figure 1E).

Although covering a more extensive binding surface, VPS35L N-terminal sequences closely mimic VPS29 association with PL motifs in Retromer accessory proteins, including the RAB guanine nucleotide exchange factor ANKRD27, and the RAB7 GTPase-activating protein (GAP) TBC1D5, and in the Retromer hijacking effector RidL from *Legionella pneumophila*^46^^,^^47^^,^^63^ (Figure S1B). This implies that Retriever will be excluded from interacting with these Retromer accessory proteins. Indeed, recombinant Retriever failed to bind recombinant TBC1D5 (Figure S1C), GFP-TBC1D5 failed to bind endogenous Retriever in cells, and GFP-VPS35L failed to isolate endogenous TBC1D5 (Figures S1D and S1E). Retriever did not regulate the RAB7 GAP activity of endosomal TBC1D5, as CRISPR/Cas9 knockout VPS35L HeLa cells did not phenocopy the defect in endosomal maturation and accumulation of hyperactivated RAB7-GTP observed in Retromer KO cells^64^ (Figures S1F and S1G). Lastly, Retriever was not bound by over-expressed mCherry-RidL (Figure S1H). Retromer binding to TBC1D5 and RidL was observed in all controls. These results establish that while Retromer-assembled VPS29 provides a docking site for accessory proteins that regulate RAB GTPases;^46^^,^^47^^,^^63^ in Retriever, this binding site is occluded, and VPS29 serves to facilitate association with the CCC complex.

We finally assessed the functional significance of Retriever in recycling α5β1-integrin. The interrelated effects of VPS29 association on CCC complex coupling precluded a mechanistic dissection of recycling using VPS35L mutants targeting VPS29 binding. However, we confirmed the importance of VPS26C interaction using VPS35L(R293E), which blocks VPS26C binding but retains VPS29 and CCC complex association (Figure 1C). In VPS35L KO HeLa cells, loss of VPS35L induced a reduction in VPS26C levels, indicating their reciprocal requirement for Retriever stability. Knockout of VPS35L also resulted in a loss of CCC complex association with endosomes, demonstrating a requirement of Retriever for CCC endosome recruitment and an increased colocalization of α5β1-integrin with the LAMP1-positive late endosome/lysosome (Figures 2D–2F) with a corresponding reduction in surface α5β1-integrin^6^ (Figure 2E). These phenotypes were all rescued by wild-type VPS35L, but not the VPS26C-binding mutant VPS35L(R293E) (Figures 2D–2F). VPS26C therefore plays a central role in the endosomal association and function of Commander through a mechanism that may include association with the cargo adaptor SNX17,^6^ the WASH complex,^65^ and/or an inherent ability of VPS26C to associate with membranes.^60^

### COMMD proteins assemble into distinct heteromeric complexes

**Figure 3:**
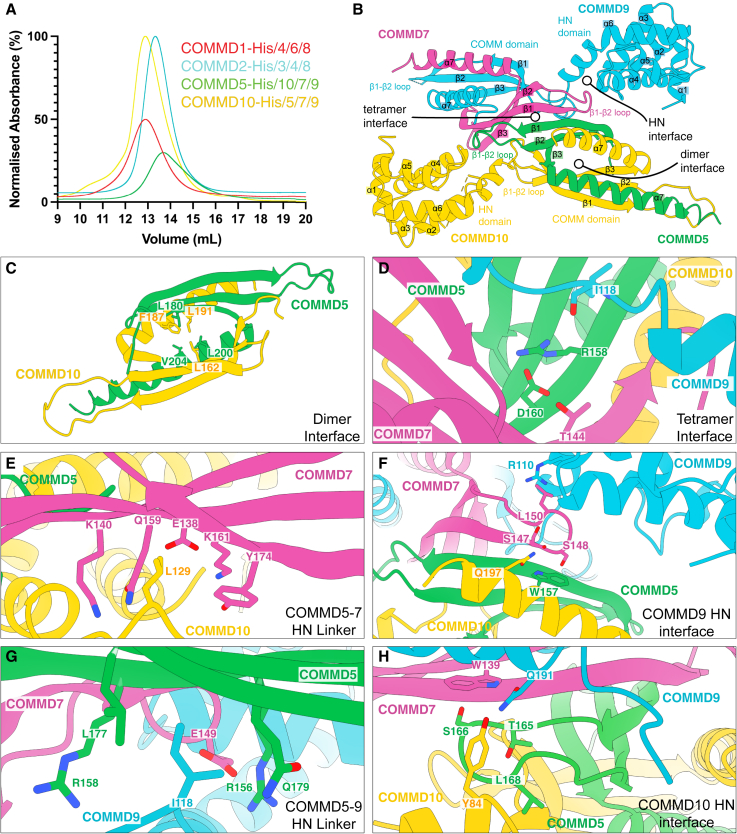
The COMMD proteins assemble into specific heteromeric complexes (A) Purification of COMMD sub-complexes. The ten human COMMD proteins were co-expressed in *E. coli* and purified via His-tags on different subunits (Methods S1) followed by gel filtration. Peptide mass spectrometry identified the subunits co-purified with each tagged protein (Methods S1) and reveals three distinct stable tetrameric complexes of COMMD1-6-4-8 (subcomplex A), COMMD2-3-4-8 (subcomplex B), and COMMD5-10-7-9 (subcomplex C). (B) 3.3-Å crystal structure of the tetrameric subcomplex C (COMMD5-10-7-9), primarily built around the three major binding interfaces shown in more detail below. (C) Key residues involved in the COMMD5-COMMD10 interface. (D) Key residues that form a β-sheet extension between COMMD5-COMMD10 and COMMD7-COMMD9 dimers. (E) Key COMMD10 residue Leu129 binds in a hydrophobic pocket to stabilize the tetramer. (F) The unique COMMD9 HN domain interface in which residues form stable and specific tetrameric interactions focusing on Trp157 of COMMD5. (G) Key interactions involving the COMMD9 linker between the HN and COMM domains centered around Ile118. (H) Similar to (F) showing the COMMD10 HN domain interactions with three subunits, centered on the COMMD7 Trp139 conserved sidechain.

To define the stoichiometry and structure of the CCC complex, we co-expressed all ten human COMMD proteins in *Escherichia coli*. Four polycistronic vectors were designed, each tagged on a different COMMD protein (Methods S1). In parallel experiments, affinity purification of these four tagged-proteins followed by gel filtration and peptide mass spectrometry resulted in the isolation of three homogeneous and stable tetrameric sub-complexes: COMMD1-4-6-8 (isolated by COMMD1-His), COMMD2-3-4-8 (isolated by COMMD2-His), and COMMD5-7-9-10 (isolated by COMMD5-His or COMMD10-His), which we referred to as subcomplex A, B, and C, respectively (Figure 3A; Methods S1).

While each COMMD subcomplex was relatively stable, only subcomplex C produced an X-ray crystal structure (3.3 Å resolution), revealing a 1:1:1:1 heterotetrameric assembly formed by COMMD5-COMMD10 and COMMD7-COMMD9 heterodimers (Figure 3B, Table S2). The four COMM domains form an intimately assembled core structure with HN domains of COMMD9 and COMMD10 located peripherally. No clear densities of COMMD5 or COMMD7 HN domains were seen, presumably due to flexibility. Notably, the structure closely matches that predicted by AlphaFold2 multimer^56^^,^^66^ (Methods S1). As expected, the four COMM domains are structurally similar, composed of three anti-parallel β-sheets followed by a C-terminal α-helix.^42^

The COMMD5-COMMD10 and COMMD7-COMMD9 dimers interact primarily via an extended β-sheet augmentation between COMMD5 and COMMD7 COMM domains (Figures 3B–3D). In addition, contacts between the HN and COMM domains of all four COMMD proteins contribute to the overall specificity of assembly. Two critical contacts involve Leu129 in COMMD10 and the analogous Ile118 in COMMD9; these lie within the respective linkers between the HN and COMM domains (Figures 3D and 3E). The linkers position these sidechains to reach across the tetramer interface and fit into complementary pockets on the distal COMMD7 and COMMD5 subunits, respectively. In addition, the two HN domains themselves interact with the other three respective subunits. For clarity, we describe these two interfaces as centered around Trp157 of COMMD5 and Trp139 of COMMD7 (Figures 3F and 3H), as these Trp residues are the *only* residues that are strictly conserved across all homologues and species of every COMMD protein.^42^ Each of these interfaces involves the HN domain enfolding the loop between the β1-β2 strands of the COMM domain from their respective dimerization partner. In this complex, the HN domain of COMMD10 enfolds the loop of the COMMD5 COMM domain, while the HN domain of COMMD9 enfolds the corresponding loop of COMMD7. In both interfaces, a serine in the β1-β2 loop forms a stacking interaction with the sidechain and a hydrogen bond with the backbone NH of the strictly conserved tryptophan residue of the neighboring COMM domain; Ser148 of COMMD7 forms a stacking and backbone hydrogen bond with Trp157 of COMMD5, while in the second interface, Ser166 of COMMD5 forms a stacking interaction and hydrogen bond with Trp139 of COMMD7. Other interactions also contribute to the specificity of each individual network (Figures 3G–3H). For example, Glu149 of COMMD7 makes a polar contact with Arg156 of COMMD5, and Tyr84 in the COMMD10 HN domain forms a hydrogen bond with Gln191 in COMMD9.

### Structure of the twelve-subunit core CCC complex

To examine the role of CCDC22 and CCDC93 in COMMD interactions and assembly of the CCC complex, we cloned all ten human COMMD proteins and CCDC22 and CCDC93 into a single biGBac construct for insect cell expression (Methods S1). StrepTactin-affinity isolation revealed that CCDC93-Strep and all other proteins were enriched in the desthiobiotin eluate, with size-exclusion chromatography revealing a single homogeneous peak (Methods S1). Western analysis confirmed a complex of all twelve proteins, with native PAGE and mass spectrometry consistent with a 1:1 stoichiometry assembly (Methods S1). Estimates of the molecular mass of the entire Commander complex are broadly consistent with the predicted molecular weight (570 kDa) if all subunits of Commander are present in a single copy.

**Figure S2:**
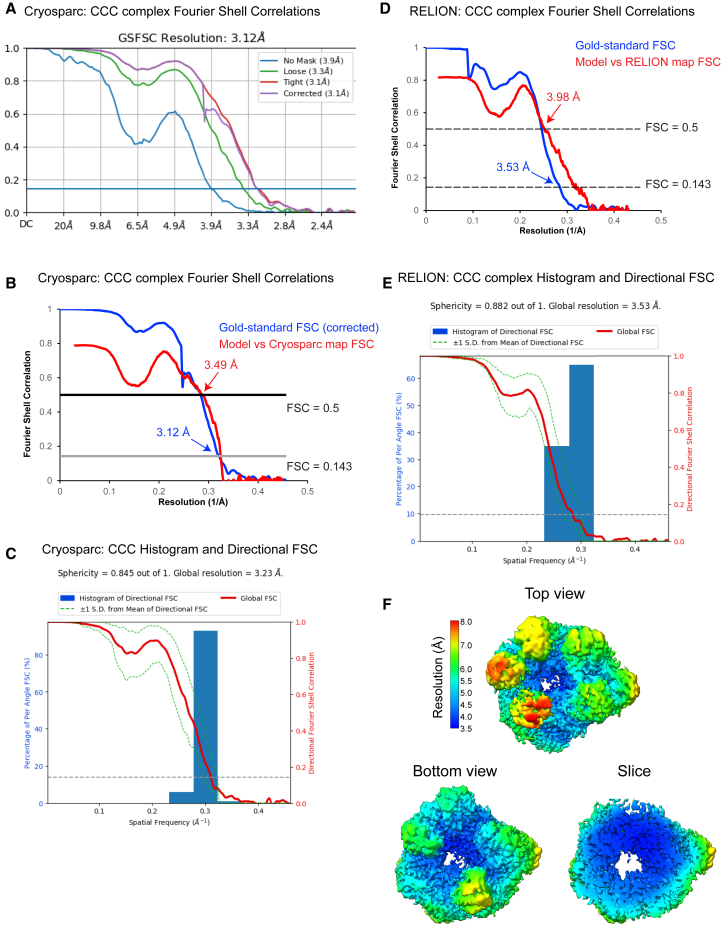
Overall, 3D and local resolution estimations, related to Figure 4 (A) Gold standard FSC (Fourier Shell Correlation) plots for the CryoSPARC reconstruction. Resolution was estimated at FSC = 0.143. (B) Gold-standard (blue) and map vs. model (red) FSC plots. Resolution of gold-standard estimated at FSC = 0.143, model-vs-map estimated at FSC = 0.5. (C) Directional FSC plots and sphericity values for the CryoSPARC reconstruction. These were calculated using a 3D-FSC server (https://3dfsc.salk.edu/). (D) Gold standard (blue) and model-vs-map (red) FSC plots for the RELION4.0 reconstruction. Gold standard resolution was estimated at FSC = 0.143, model-vs-map resolution was estimated at FSC = 0.5. (E) Directional FSC plots and sphericity values for the RELION4.0 reconstruction. These were calculated using a 3D-FSC server (https://3dfsc.salk.edu/). (F) Local resolution estimates for the RELION4.0 reconstruction. The reconstruction was colored according to local resolution estimation in RELION.

**Figure S3:**
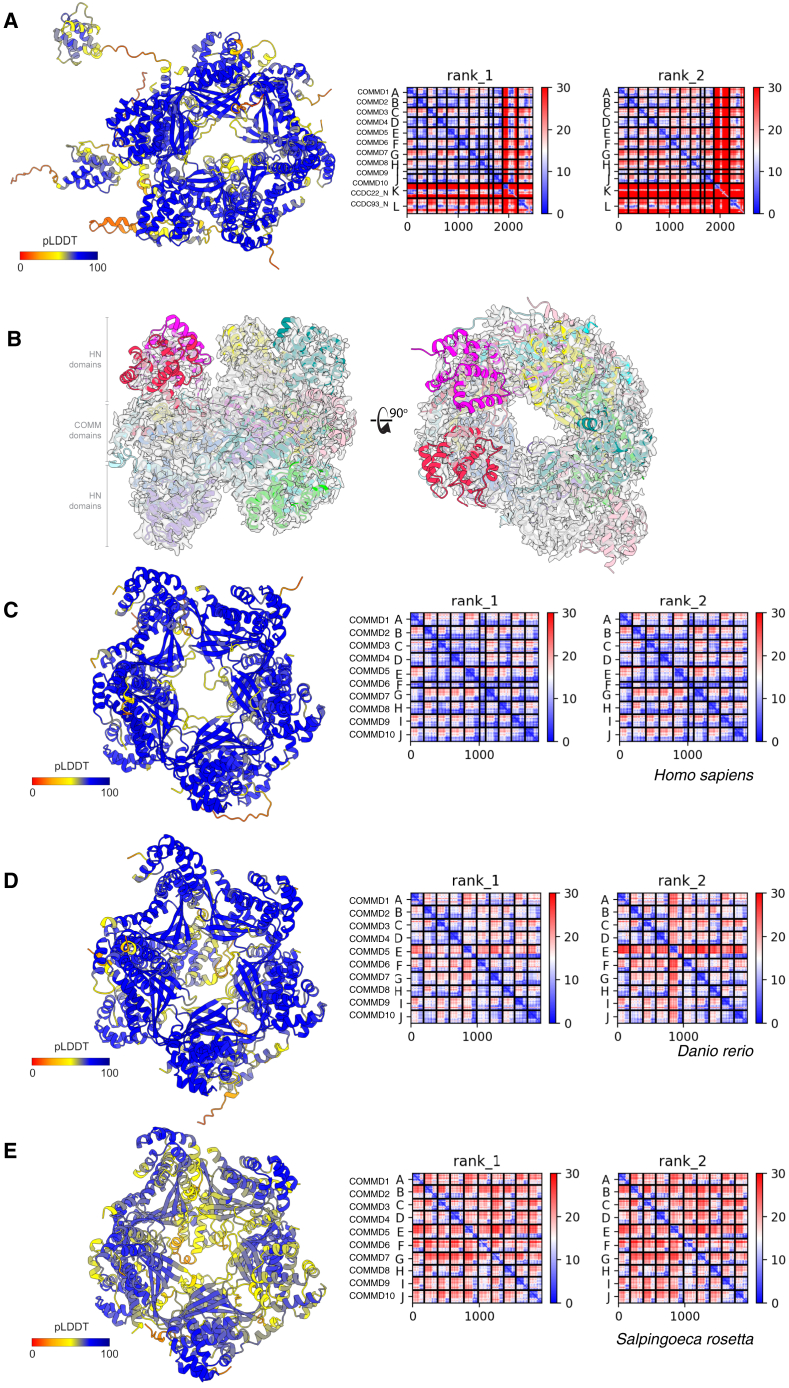
AlphaFold2 modeling of CCC complex across evolution, related to Figure 4 (A) Alphafold2 colored by the confidence metric (pLDDT) of human COMMD1-10 and the N-terminal domains of CCDC22 and CCDC93 with PAE plots of the top 2 ranked models. (B) Same view as in Figure 4A showing the fit of the core CCC subunits to the cryoEM density. (C–E) Further modeling of the COMMD decamer was conducted using sequences from (C) *Homo sapiens*, (D) *Danio rerio*, and (E) *Salpingoeca rosetta*. Each model displayed highly connected structural correlations between subunits based on PAE plots and consistent decamer assembly.

Purified BS3-crosslinked CCC dodecameric complex was vitrified on graphene-oxide coated grids for single particle cryoEM (Figure S2; Methods S1). Data processing in CryoSPARC yielded a 3D reconstruction (overall resolution 3.1 Å) (Figure S2; Methods S1; Table S1). Initially, a model of the dodecamer including all ten COMMD proteins and the N-terminal regions of CCDC22 and CCDC93 was constructed using AlphaFold2 multimer^54^^,^^55^^,^^56^ (Figure S3A). This predicts a specific arrangement of the COMMD proteins in a heterodecameric ring, with linker regions of CCDC22 and CCDC93 between their N-terminal calponin-homology (CH) domains and their C-terminal coiled-coil domains entwined through the COMMD assembly. The model was docked into the cryoEM map with minimal adjustments and refined to produce an initial structure (Figure S3B). The central ring of the COMM domains was more clearly resolved than the peripheral HN domains due to their relative flexibility (Video S2; Methods S1). To partially address this issue, we re-processed the data in RELION4.0. Several rounds of 3D particle classification with and without alignment combined with 3D refinement yielded a reconstruction with overall resolution of 3.5 Å (Figure S2; Methods S1; Table S1). The HN domains of the COMMD proteins and the CH domain of CCDC93 were better resolved in this map, albeit at lower resolution than the central core of COMM domains, facilitating further refinement (Figure S2; Methods S1). Notably, an essentially identical *ab initio* structure was built using the machine-learning guided modeling software Modelangelo,^67^ although incomplete in many HN domains due to poorer density (not shown). Although the complex studied by cryoEM includes the full-length CCDC proteins, no density is observed for the CH domain of CCDC22 or the C-terminal coiled-coil domains of either protein, indicating significant flexibility in their relative orientation to the COMMD ring. Weak density is observed for the CH domain of CCDC93, which is stabilized by its interaction with the HN domain of COMMD4.

**Figure 4:**
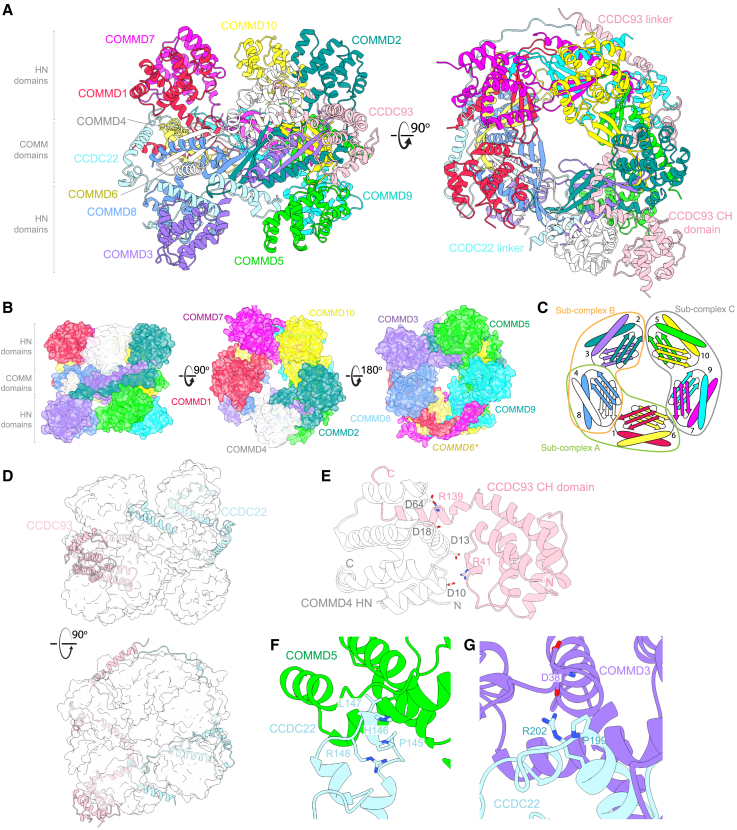
CryoEM structure of the human CCC complex (A) CryoEM structure of the CCC complex revealing the COMMD proteins, the CH domain of CCDC93, and linker regions of CCDC22 and CCDC93. Linker domains of CCDC22 and CCDC93 visible in our cryoEM map form intricate interactions with the decameric COMMD structure, leading to a highly intertwined structure. The CH domain of CCDC22 and extended coiled-coil regions of the CCDC proteins are not visible in current cryoEM maps due to flexibility relative to the stable COMMD decamer. (B) Molecular surface highlighting the organization of the HN domains of COMMD1, 4, 2, 10, and 7 on one side of the COMM domain ring, and COMMD8, 3, 5, and 9 on the other side. Human COMMD6 lacks the HN domain. For clarity, CCDC22 and CCDC93 are omitted. (C) Schematic model of COMMD decamer and arrangement of the sub-complexes. (D) Interweaving of CCDC22 and CCDC93 within the COMMD ring. (E) Interactions stabilizing the CCDC93 CH domain contact with the central COMMD ring, via the HN domain of COMMD4. (F and G) The PxxR sequences in CCDC22 that form turn structures: (F) the ^145^PHLR^148^ motif binds the HN domain of COMMD5; and (G) the ^199^ PVGR^202^ motif binds the COMMD3 HN domain. See also Figures S2, S3, S4, and S5.

Video S2. Structure of the CCC complex determined by cryoEM, related to Figure 4

**Figure S4:**
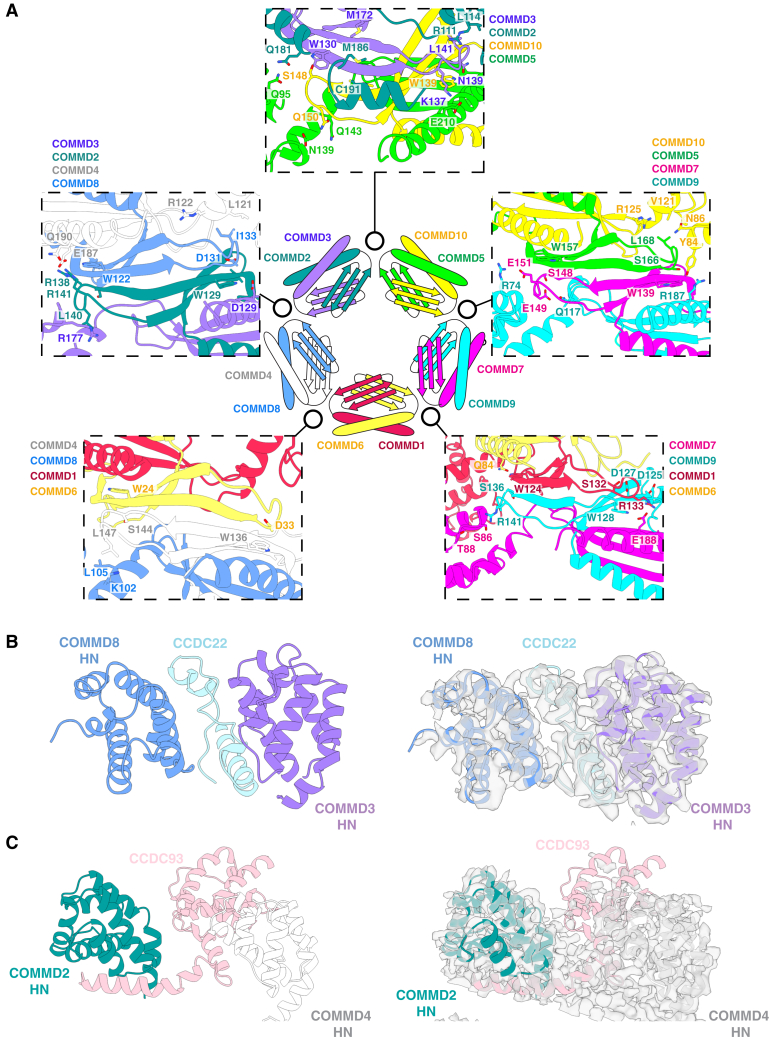
CCDC22 and CCDC93 linkers make extensive contacts with the central COMM domain ring and peripheral HN domains, related to Figure 4 (A) Details of the five interfaces between the COMMD heterodimers of the heterodecameric ring. The central schematic is as shown in Figure 4D to provide a reference for each interface. Structural panels show adjacent heterotetramers in the same orientation, placing the strictly conserved Trp sidechain of each subunit as the focal point. Many specific interactions between adjacent subunits determine the precise COMMD organization. (B and C) Interfaces between CCDC22 and the HN of COMMD3 and COMMD8, and (C) between CCDC93 and HN domains of COMMD2 and COMMD4.

The human COMMD proteins assemble into a remarkable hetero-decameric closed ring (Figures 4 and S3B). The arrangement of subunits around the ring follows a strict order of five heterodimers of (COMMD1-6)-(COMMD4-8)-(COMMD2-3)-(COMMD10-5)-(COMMD7-9) (Figures 4B and 4C). This cryoEM structure is consistent with *(i)* the COMMD10-5-COMMD7-9 crystal structure, *(ii)* the tetrameric sub-assemblies observed in bacterial expression, and *(iii)* the complex predicted with AlphaFold2 across diverse species (Figures S3C–S3E). One surface of the ring is decorated by the HN domains of COMMD1, 4, 2, 10, and 7, while the other consists of COMMD8, 3, 5, and 9 (human COMMD6 lacks the HN domain, although it is present in other species) (Figure 4B). As seen in the crystal structure of the COMMD5-7-9-10 heterotetramer, the interface between each COMMD heterodimer is mediated by specific contacts involving the four adjacent protomers (Figure S4A), resulting in the precise organization of the heterodecameric ring.

In this structure, the CCDC22 and CCDC93 linkers make extensive contacts with the central COMM domain ring and the peripheral HN domains (Figures 4D, S4B, and S4C), while the CCDC93 CH domain is partly stabilized by direct interactions with the COMMD ring via the HN domain of COMMD4 (Figure 4E). Two PxxR sequences in CCDC22 that form similar turn structures, ^145^PHLR^148^ (Figure 4F) and ^199^PVGR^202^ (Figure 4G), bind the HN domains of COMMD5 and COMMD3, respectively. The extensive interactions mediated by the linker regions of the CCDC proteins appears to enhance the stability of the COMMD ring and likely explains why only tetrameric sub-complexes are isolated when expressing the COMMD proteins alone in *E. coli* (Figure 3). The structure is also consistent with truncation analyses that found N-terminal regions of CCDC22 and CCDC93 could interact with COMMD proteins but not Retriever.^21^^,^^65^ The conserved structure strongly implies that COMMD and CCDC22/CCDC93 proteins will function strictly as a dodecameric complex in the cell.

**Figure S5:**
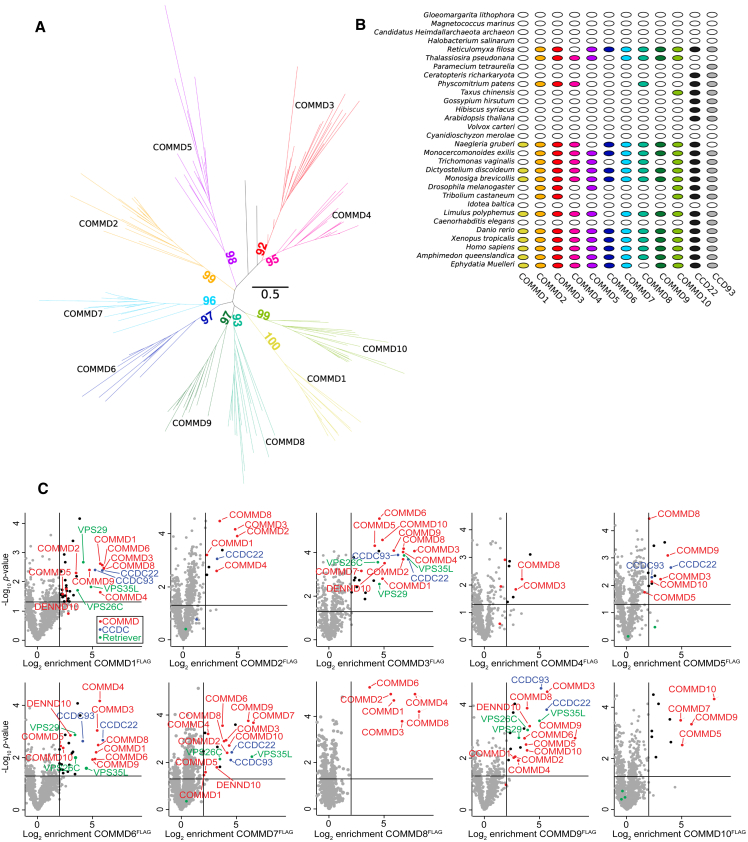
Evolutionary origins of the CCC complex, related to Figure 4 (A) Maximum-likelihood phylogeny of COMMD1-10 proteins from 23 representative eukaryotic taxa inferred under the best-fitting Q.yeast+R5 substitution model. Each COMMD forms a strongly supported (>90% bootstrap) clan in the unrooted phylogeny, and each clan contains representatives from all major lineages (supergroups) of eukaryotes; this implies that all ten COMMD subunits were already present in the last eukaryotic common ancestor (that is, LECA appears 10 times in the tree). Based on the absence of COMMD homologues in Bacteria and Archaea, this protein family likely originated on the eukaryotic stem and proliferated via a series of gene duplications prior to the radiation of the modern eukaryotic groups. (B) Presence-absence patterns of COMMD and CCDC22/CCDC93 in a set of representative modern eukaryotes. The presence-absence pattern of COMMD genes in modern eukaryotes, taken together with the phylogeny in (A), indicates that these genes have been lost independently in different eukaryotic lineages. (C) Digitonin-solubilized COMMD knockout eHap cell lines expressing the indicated COMMD^FLAG^ construct were affinity enriched using Flag agarose beads followed by label free quantitative proteomics. The threshold of significant enrichment was determined to be 2-fold (log2 fold change = 1) based on the distribution of unenriched proteins. Black and colored dots indicate significantly enriched proteins. Red, COMMD subunits; Blue, CCDC subunits; Green, Retriever subunits.

Phylogenetic analysis of the COMMD subunits CCDC22 and CCDC93 demonstrated that all twelve proteins were present in the last eukaryotic common ancestor (LECA),^2^ with no sequence homologues identified in Archaea or Bacteria (Figures S5A and S5B). This suggests the COMMD subunits likely arose by gene origination followed by gene duplication along the eukaryotic stem lineage, prior to LECA. The distribution of the ten subunits across extant eukaryotes involves parallel, lineage-specific loss in some species. These losses continued following the diversification of the major eukaryotic lineages; for example, within embryophytes (land plants), the model tracheophyte *Arabidopsis thaliana* appears to have lost Commander entirely, while the bryophyte *Physcomitrium patens* has retained four of the ancestral subunits (Figure S5B).

To further assess the interdependence of Commander subunits, the ten human COMMD proteins were each knocked out in eHAP cells, corresponding COMMD proteins were re-expressed with a C-terminal FLAG tag, immunoprecipitated, and analyzed by peptide mass spectrometry (Figure S5C). When used as bait, COMMD1, 3, 6, 7, and 9 specifically isolated the entire Commander assembly, confirming the overall inter-stability of the complex. In contrast we noted that COMMD2, 4, 5, 8, and 10 FLAG-tagged proteins were enriched only with specific subsets of COMMD proteins, which correlated with the sub-complexes observed in bacterial co-expression experiments in the absence of the CCDC proteins. Examination of the CCC structure suggests that the C-terminal FLAG-tags in these COMMD subunits may affect specific contacts with CCDC22 and CCDC93. We speculate this leads to the loss of CCDC interactions, causing disruption of the CCC complex and loss of Retriever interaction, and further validates their importance for overall assembly. The interdependency of the COMMD and CCDC proteins for Commander organization provides a molecular explanation for the high degree of subunit conservation across species.

### DENND10 is recruited by the coiled-coil domains of CCDC22 and CCDC93

**Figure 5:**
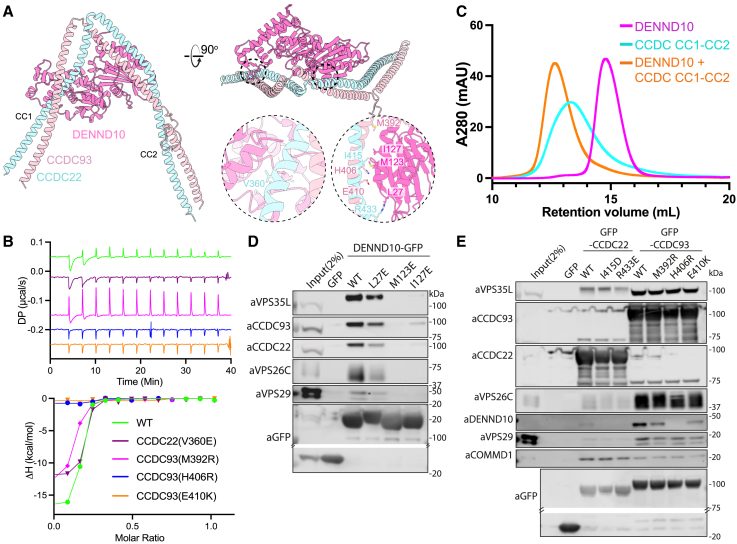
DENND10 associates with the central coiled-coil domains of CCDC22 and CCDC93 (A) Structure of DENND10 complex with the dimeric CC1 and CC2 coiled-coil regions of CCDC22 and CCDC93 predicted by AlphaFold2. Model quality and predicted alignment errors are shown in Figure S6. (B) DENND10 was titrated into purified wild-type and mutant CC1-CC2 complexes (CCDC22(325–485) + CCDC93(310–488)) and binding was measured by ITC. *Top* shows the raw data and *bottom* shows the integrated and normalized data fitted with a 1:1 binding model. The binding affinities were as follows: WT, 34 nM ± 0.5 nM; CCDC22 (V360E), 47.9 nM ± 3.5 nM; and CCDC93 (M392R), 89.1 nM ± 10 nM. No binding was detected for CCDC93H406R or E410K. (C) Analytical size exclusion chromatography of DENND10 (magenta), CC1-CC2 complex (cyan), and DENND10 mixed with CCDC22-CCDC93 forming a stable complex (orange). (D and E) GFP-nanotrap of GFP-DENND10 (D) or CCDC22 and CCDC93 mutants. Data S1 shows quantified band intensities and raw blots (n = 3). See also Figure S6.

**Figure S6:**
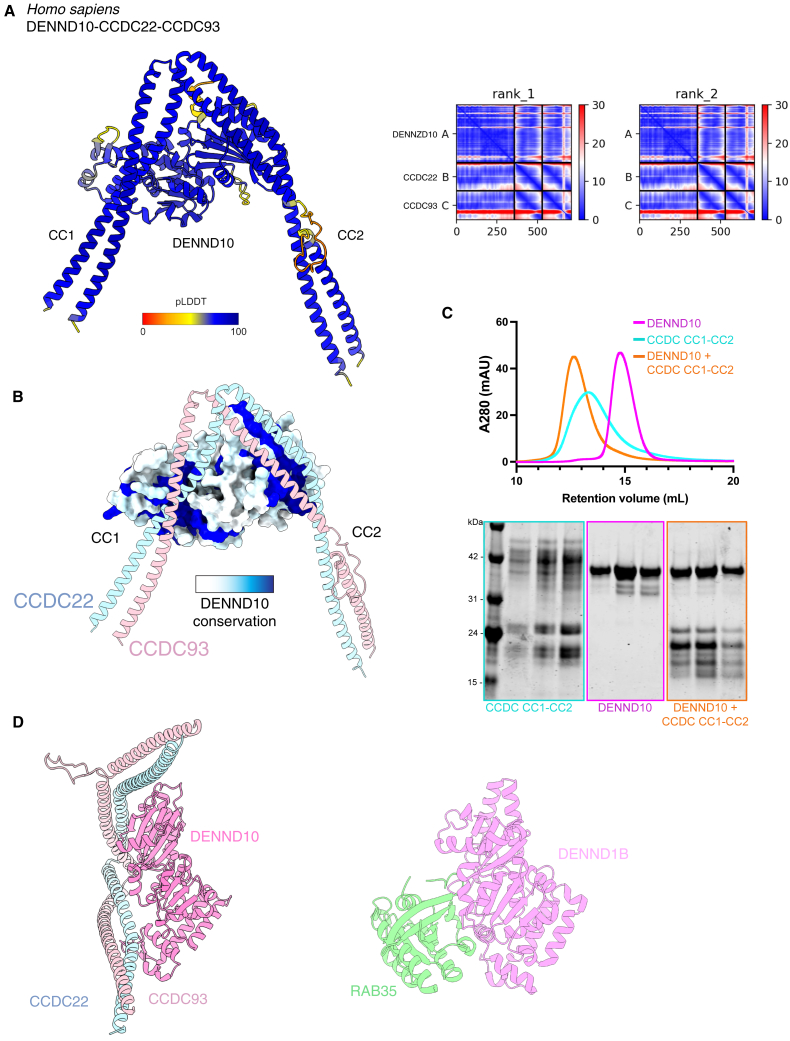
AlphaFold2 modeling of the DENND10-CCDC22-CCDC93 complex, related to Figure 5 (A) AlphaFold2 of human DENND10 and the CC1-CC2 coiled-coil domains of CCDC22 and CCDC93 colored by the confidence metric (pLDDT). The PAE plots of the top 2 ranked models are shown. (B) Same model as in (A) but with DENND10 shown in surface representation colored according to conservation with CONSURF.^113^ (C) Top panel shows analytical size exclusion chromatography of DENND10 (magenta), CC1-CC2 complex (cyan) and DENND10 mixed with the CCDC22-CCDC93 forming a stable complex (orange). Bottom panel shows Coomassie stained gel of the peak fractions. (D) Predicted DENND10 structure bound to CCDC22-CCDC93 in the same orientation alongside the crystal structure of DENND1B in complex with RAB35 (PDB ID: 3TW8).^70^.

As seen previously,^6^^,^^16^^,^^20^^,^^21^^,^^22^^,^^68^^,^^69^ our proteomic analyses routinely identified DENND10 as a Commander subunit (Figure S5C), although it is not required for Commander stability, and its deletion does not affect recycling of α5 integrin.^21^ AlphaFold2 predicted a high confidence complex between DENND10 and a dimer of two central coiled-coil regions from CCDC22 and CCDC93 (CC1 and CC2) (Figures 5A and S6A). In this predicted structure, CCDC22 and CCDC93 form a V-shaped coiled-coil dimer bridged by conserved elements of the DENND10 DENN domain (Figures 5A and S6B). Consistent with this, the CC1-CC2 coiled-coil regions of CCDC22 and CCDC93 formed a stable dimer, which bound to recombinant DENND10 with an affinity of 28 ± 6 nM (Figure 5B). The formation of a high-affinity trimer was also shown by size-exclusion chromatography (Figures 5C and S6C). Mutations in CCDC22 and CCDC93 within the predicted binding interface either reduced or abolished the interaction to below detectable levels (Figure 5B).

We further validated this complex in cells (Figures 5D and 5E). GFP-tagged DENND10 was able to precipitate Commander subunits, while mutations in the predicted interface DENND10(L27E), -(M123E), and -(I127E) either reduced or abolished these interactions. Reciprocal mutations in CCDC22 and CCDC93 also perturbed cellular interaction with DENND10, with the CCDC93(H406R) and CCDC93(E410K) mutations showing the strongest effect in line with the *in vitro* ITC measurements. Although DENN domains are generally thought to act as RAB GEFs, the only structure of a DENN domain-RAB complex is of DENND1B and RAB35.^70^ The DENND10 sequence is highly divergent from DENND1B, and no putative RAB effector protein(s) have yet been identified, although there is evidence for an association with RAB27.^69^ Comparison with the DENND1B-RAB35 complex suggests that the CCDC proteins bind to DENND10 using an overlapping surface. Thus, when associated with Commander, DENND10 would be unable to engage a RAB GTPase in the same way as DENND1B with RAB35 (Figure S6D).

### Overall structure of the holo-Commander complex and disease mutations

Encouraged by the excellent agreement of experimental crystal and cryoEM structures with AlphaFold2 modeled complexes, we performed further predictions to assess how Retriever and the CCC complex assemble to form the Commander complex (see Methods). Full-length CCDC22 and CCDC93 are predicted to form a heterodimer with four coiled-coil regions (CC1-CC4) in two V-shaped structures, the first of which interacts with DENND10 (Figure 5). Our cryoEM structure shows that the N-terminal CH domain of CCDC93 is closely associated with the COMMD ring via the COMMD4 HN domain (Figure 4). In contrast, the CH domain of CCDC22, (which is not visible in the cryoEM map) is predicted to form an intramolecular interaction with the two C-terminal coiled-coil regions (CC3-CC4). Interestingly, the two CCDC proteins share distant structural similarity with IFT subunits of the intraflagellar transport (IFT) machinery, which form comparable coiled-coil dimers with N-terminal CH domains (Methods S1).^71^^,^^72^

**Figure 6:**
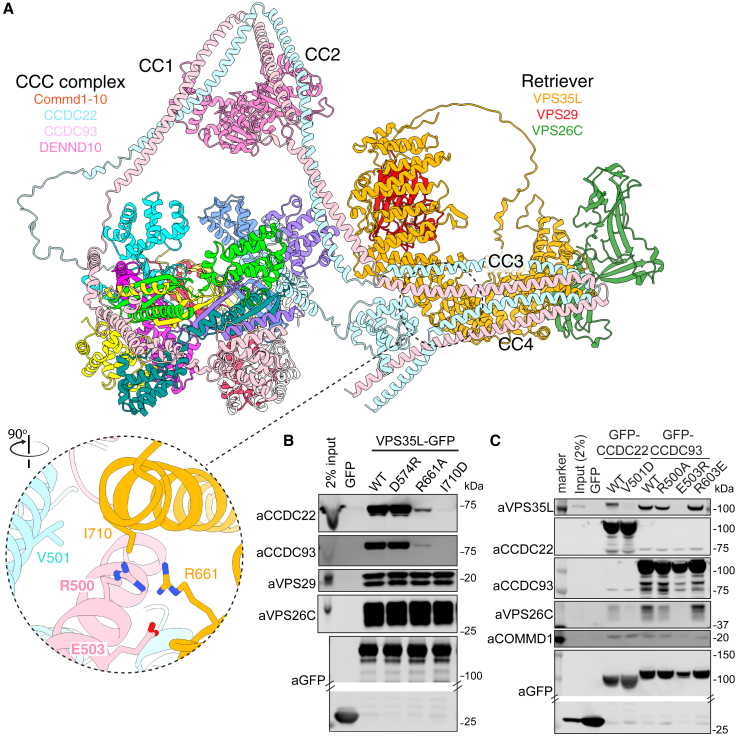
Assembly of the Commander holo-complex (A) Model of Commander complex combining cryoEM and crystal structures of the CCC and Retriever sub-assemblies and AlphaFold2 modeling of the coupling of CCC and Retriever via the C-terminal coiled-coil regions of CCDC22 and CCDC92 and the CH domain of CCDC22. The general approach is shown in Methods S1. (B) GFP-nanotrap of GFP-VPS35L wild type (WT) and mutants targeting the predicted interface with the CCC complex. (C) GFP-nanotrap of GFP-CCDC22 or CCDC93 mutants targeting the predicted interface with Retriever. Data S1 shows quantified band intensities and raw blots (n = 3). See also Figures S6 and S7.

After comprehensive testing of potential assemblies, we identified an unambiguous interaction linking the CCC and Retriever complexes between a conserved surface on VPS35L (opposite the VPS29-binding site) and the CCDC22-CCDC93 proteins (Methods S1). This is mediated primarily by the C-terminal CC3-CC4 coiled-coil structures with a minor interface involving the second CC2 region. By combining our experimental structures with AlphaFold2-derived models, we developed a structure of the sixteen subunit Commander complex (Figure 6A; Methods S1; Video S3). The decameric COMMD ring and trimeric Retriever are tethered by the heterodimeric CCDC22 and CCDC93 proteins, with DENND10 associated at the apex of the structure. As mentioned, the interaction of the CCDC proteins with Retriever is mediated by the V-shaped CC3-CC4 segment at their C terminus associating with VPS35L at a conserved surface distal from VPS26C and VPS29. The overall shape of the complex is restrained by the predicted intramolecular interaction between the CCDC22 CH domain with this CCDC22/93 C-terminal structure. We validated the major interface by mutagenesis of key residues, with VPS35L(R661A) or VPS35L(I710D) and CCDC22(V501D) or CCDC93(E503R) all specifically perturbing Retriever and CCC complex association without affecting assembly of either subcomplex (Figures 6B and 6C).

Video S3. Overall model of the Commander complex derived from cryoEM, X-ray crystallography, and AlphaFold2 modeling, related to Figure 6

**Figure S7:**
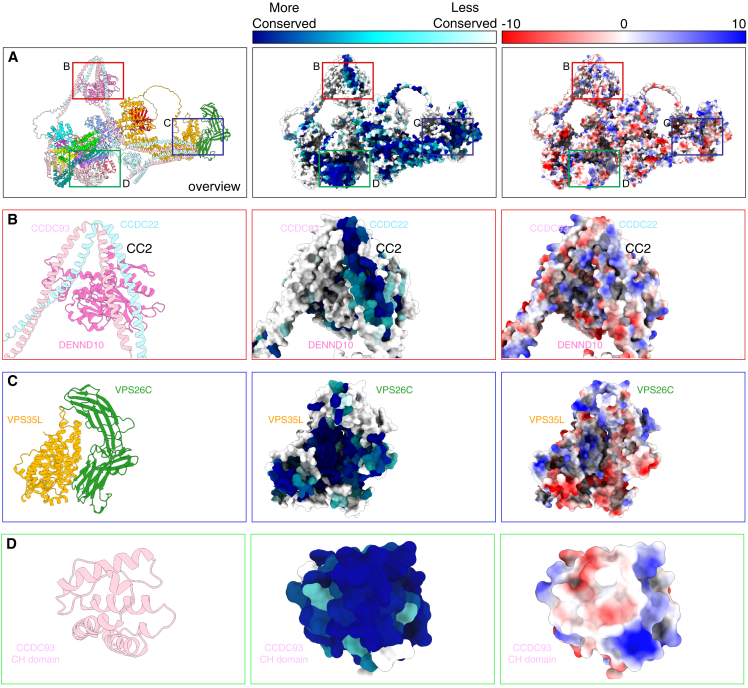
Conserved and electrostatic surfaces of the Commander complex, related to Figure 6 (A) Overview of Commander as a ribbon diagram (left), with conserved surfaces mapped with CONSURF (middle)^113^ and with electrostatic surface potential calculated with ChimeraX.^106^. (B) CCDC22-CCDC93-DENND10 interface where a conserved surface aligns with a region for binding the WASH complex subunit FAM21.^65^ (C) Conserved pocket in VPS35L-VPS26C interface.^6^ (D) CCDC93 CH domain showing highly conserved surface properties.

In Figure S7 we plotted the electrostatic surface potential of Commander as well as surface conservation to highlight regions of likely functional importance. The electrostatic surface does not reveal any regions suggestive of binding to negatively charged phospholipid membranes.^60^^,^^73^^,^^74^ In contrast, there are surfaces that show a high degree of conservation. The first is a patch on the CCDC22-CCDC93 coiled-coil structure, lying adjacent to DENND10 (Figure S7B) aligning closely with a region required for interacting with FAM21 of the WASH complex.^65^ A second pocket is formed by the interface between VPS35L and VPS26C (Figure S7C). Previously, VPS26C was shown to be required for coupling to the SNX17 cargo adaptor,^6^ and we speculate this pocket may be involved in SNX17 recruitment. Lastly, the surface of the CCDC93 CH domain is very highly conserved (Figure S7D). Given the general actin-binding activity observed for CH domains, we speculate it might mediate cytoskeletal interactions.

**Figure 7:**
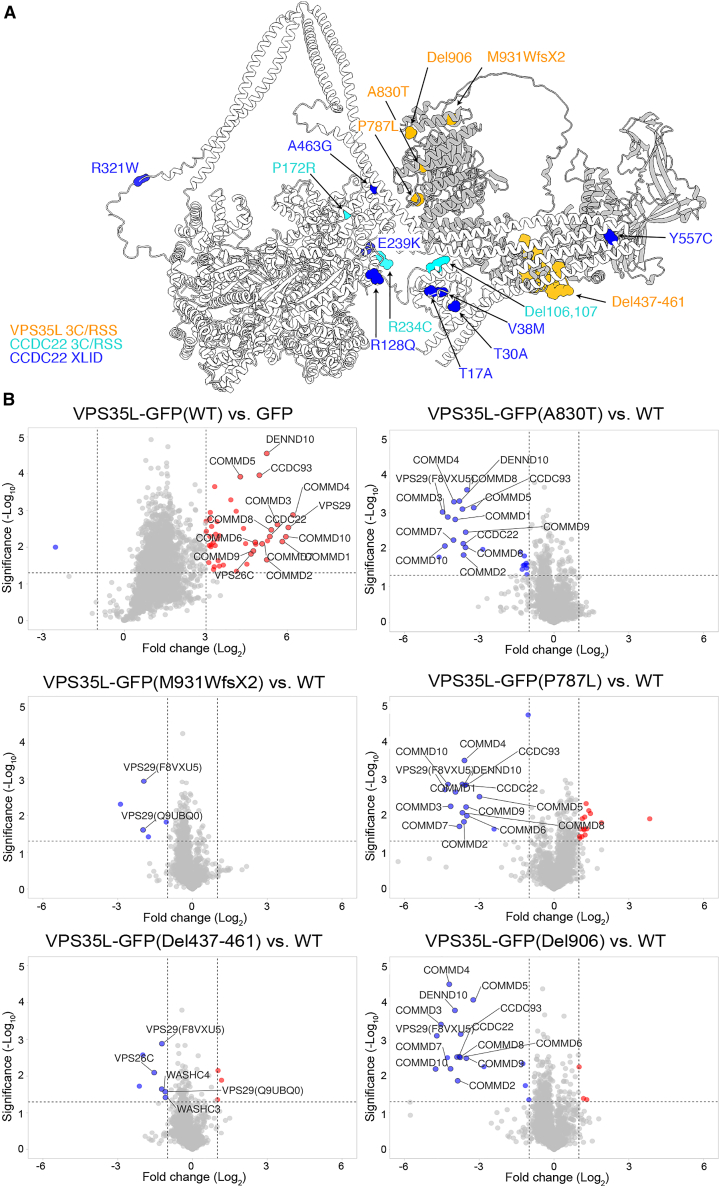
Structural and functional impacts of Commander mutations causing XLID and RSS (A) VPS35L and CCDC22 mutations associated with XLID and RSS mapped onto the Commander structure. (B) Volcano plots of enriched (red circles) or depleted (blue circles) interactors in seven-plex TMT-based proteomics comparing GFP-VPS35L wild-type and GFP-VPS35L mutants causative for RSS (n = 3).

Finally, we mapped mutations causative for XLID and RSS^7^^,^^8^^,^^9^^,^^10^^,^^11^^,^^12^ onto the Commander model (Figure 7A). This reveals clustering of VPS35L and CCDC22 pathogenic mutations around the interface between Retriever and CCC complexes, providing insight into the destabilization of protein expression observed in patients harboring these mutations.^8^^,^^9^^,^^11^^,^^12^ CCDC22(Y557C) is a highly conserved sidechain and lies directly within the interface with VPS35L. The CH domain of CCDC22 is predicted to form a key interaction with the C-terminal coiled-coil domains of CCDC22 and CCDC93 resulting in an overall compact Commander structure (Figure 6A), and a cluster of disease-causing mutations (T17A, T30A, V38M, R128Q) are predicted to destabilize this domain and its intramolecular interaction. VPS35L pathogenic mutations A830T, Del906, and P787L cluster toward the VPS29 interface and are anticipated to disrupt the C-terminal structure of the VPS35L solenoid. Unbiased interactome analysis comparing wild type VPS35L with the three mutants confirmed a pronounced loss in VPS29 and CCC complex association (Figure 7B). In contrast, the VPS35L(M931Wfs^∗^2) and VPS35L(Del437-461) mutants^11^ retained CCC complex association but had reduced VPS29 binding (Figure 7B). Taken alongside evidence that CCDC22(T17A), CCDC22(Y557C), and VPS35L(A830T) perturb endosomal recycling of LRP1 and LDLR and lead to hypercholesterolemia,^11^^,^^65^ these structural data provide a molecular explanation for the perturbed stability and assembly of the Commander complex associated with XLID and RSS.

## Discussion

Despite its essential role in membrane trafficking and importance in disease, the molecular structure of Commander has been mostly unexplored. Our studies provide a comprehensive understanding of how Retriever and CCC complexes are assembled and how they combine to form the Commander super-complex. The conservation of this complex confirms its essential role throughout evolution, and the structure provides an atomic level description of its organization that explains previous results including the co-dependence of each of the COMMD, CCDC, and VPS35L proteins for complex stability.^13^^,^^28^ This structure also indicates that each of the subunits act in unison to mediate Commander activity and implies that previous studies examining individual components of the complex, including our own,^42^ may need to be re-interpreted.

### The structure and function of Retriever are distinct from those of Retromer

Retromer is a well-characterized complex that works with different cargo adaptors including SNX3 and SNX27 to facilitate endosomal sorting.^5^ Although Retriever shares similarities with Retromer, composed of related VPS35L and VPS26C subunits and the shared VPS29 protein, and associates with one divergent adaptor SNX17,^2^^,^^3^^,^^6^^,^^15^ whether it assembled or functioned in a similar manner was unclear. Our studies have shown an analogous architecture; however, Retriever is more compact and has distinct conserved surfaces that mediate specific interactions with the CCC machinery and an intrinsically unstructured N-terminal region that binds and regulates VPS29. This sequence mimics Pro-Leu-containing motifs found in Retromer-interacting proteins, including the RAB7 GAP TBC1D5,^47^ the RAB32/RAB38 GEF and SNARE trafficking protein VARP/ANKRD27,^46^ and the secreted *L. pneumophila* effector RidL.^48^^,^^50^^,^^51^ The VPS35L sequence binds VPS29 with high affinity, which is enhanced by intramolecular tethering and thus blocks Retriever-bound VPS29 from participating in these regulatory interactions. Incorporation of VPS29 into Retriever is therefore mutually exclusive with its ability to function in canonical Retromer-mediated transport, which also explains why synthetic macrocyclic peptides targeting the conserved pocket on VPS29 interact with Retromer but not Retriever.^44^ In human cells, VPS29 is highly abundant, typically present at up to twice the level of other Retromer subunits, and more than twenty times the concentration of other Commander subunits.^75^^,^^76^ How the equilibrium between VPS29 association with either Retromer or Retriever is regulated remains an important question.

### The CCC complex is a unique assembly of enigmatic function

The COMMD proteins have been shown to undergo homo- and heteromeric interactions using co-immunoprecipitation from cells,^6^^,^^16^^,^^17^^,^^19^^,^^21^^,^^28^^,^^40^^,^^41^^,^^42^^,^^68^^,^^77^ or following pairwise co-expression in bacterial systems.^42^^,^^77^ The proteins must form obligate dimers due to the distinct structure of their C-terminal COMM domains.^42^ However, in proteomic studies, the entire set of COMMD proteins are always identified in a complex together with other Commander subunits (this study and others^6^^,^^16^^,^^17^^,^^20^^,^^21^^,^^22^). Our data define precisely how COMMD family members interact with each other in preferred heterodimers and provide a detailed understanding of their complete assembly into a heterodecameric ring, the stability of which is dependent on interaction with CCDC22 and CCDC93. Although there are many individual requirements for precise COMMD interactions, in general the β1-β2 loop within the COMM domain of each COMMD subunit docks closely into a complementary pocket formed by the HN domain and linker of its cognate dimeric partner, and these interactions involve a strictly conserved tryptophan within the C-terminal α7-helix of the neighboring subunit.

One functional implication of the COMMD assembly is that the HN domains are positioned peripherally and appear to be primed for mediating specific intermolecular interactions. It has been proposed that the sequence divergence in the N-terminal HN domains could allow for distinct interactions mediated by different family members, for example with the NF-κB complex,^34^^,^^41^ and cytoskeletal components.^78^ Identifying whether specific functional interactions are mediated by the different COMMD subunits remains an exciting avenue of investigation.

One important finding is that stable COMMD assembly requires the intercalation of CCDC22 and CCDC93 linker sequences, which make extensive contacts with different COMMD subunits to tie the assembly together. The central coiled-coil regions of the CCDC22-CCDC93 dimer mediate recruitment of the peripheral subunit DENND10, a member of the DENN domain family, which are generally thought to act as GEFs for RAB GTPases.^79^^,^^80^^,^^81^^,^^82^ DENND10 localizes to late endosomes, and its knockdown perturbs aspects of endosomal morphology and function,^21^^,^^69^ although it appears dispensable for assembly of Commander.^21^ Only one crystal structure of a DENN domain in complex with a RAB GEF substrate has been determined,^70^ while the only other structures known are Longin and DENN domain-containing dimers of C9orf72/SMCR8 and Folliculin/FNIP2 that act as RAB GAP complexes.^83^^,^^84^^,^^85^^,^^86^^,^^87^ DENND10 is distinct from these, lacking key residues found in DENND1B required for GEF activity, and the interface with CCDC22-CCDC93 would preclude RAB binding in the same region. Whether DENND10 has GEF/GAP activity or plays a distinct role within the Commander complex remains to be determined.

### Assembly of the commander holo-complex and its role in endosomal recycling

This work provides a molecular explanation for the coupling of the CCC and Retriever assemblies into the holo-Commander complex. Although our model will require full structural confirmation, we show that mutations designed based on AlphaFold2 modeling specifically block CCC and Retriever interaction. Our data thus support the idea that while the CCC and Retriever assemblies are distinct structures, the function of these proteins likely depends on their incorporation into the Commander holo-assembly. The CCDC proteins share some structural similarity with other CH domain-containing and coiled-coil heterodimers,^88^ predominantly involved in regulating cytoskeletal interactions such as with ciliary microtubules,^66^^,^^71^^,^^72^ within dynein-adaptor assemblies,^89^^,^^90^^,^^91^ or at the kinetochore,^92^ and CH domains are often involved in direct interactions with both actin and microtubule filaments.^93^^,^^94^^,^^95^^,^^96^ Endosomal recycling by Commander requires dynamic organization of actin-rich domains on the endosomal surface.^4^ Endosome-associated branched actin is nucleated by Arp2/3 following activation by the WASH complex, which interacts with Commander subunits.^4^^,^^6^^,^^13^^,^^21^^,^^65^^,^^78^ Given this functional connection, it is tempting to speculate that CH domain interactions could be important for establishing the actin-rich microdomains required for endosomal sorting.^4^

The structure of the Commander complex has allowed us to map the locations of causative mutations for XLID and RSS.^7^^,^^8^^,^^9^^,^^10^^,^^11^^,^^12^^,^^13^^,^^14^ Most missense mutations map to key structural elements or inter-subunit interfaces, and those tested all result in significant loss in overall Commander protein levels. In contrast, we found that deletion and frameshift mutations in VPS35L proximal to the VPS29 binding site specifically impact VPS29 interaction without seriously affecting overall Commander assembly. This shows that XLID/RSS mutations can lead to either overall Commander instability or loss of specific interactions within the complex and reaffirms the important role that VPS29 plays in Commander function.

### Limitations of this study

Our work provides the overall structural framework for analyzing Commander function in a wide array of cellular and disease-associated processes. However, while core structures of the CCC complex and the interaction of VPS29 with the N-terminal tail of VPS35L have been determined at high resolution using X-ray crystallography and cryoEM, the coiled-coil regions of the CCDC proteins remain unresolved by experimental methods, and cryoEM maps of Retriever have revealed its overall architecture but not its entire atomic structure. In addition, while interactions of the CCC complex with DENND10 and with Retriever have been mapped and experimentally validated *in vitro* and *in situ*, it will still be important to obtain high resolution experimental structures of these complexes in the future. Ultimately, purification and structural studies of the full sixteen subunit Commander holo-complex will be needed to provide a complete picture of this complex, identify potential conformational rearrangements in its overall organization, and determine how it assembles on the endosomal membrane.

## STAR★Methods

### Key resources table

**Table:** 

REAGENT or RESOURCE	SOURCE	IDENTIFIER
**Antibodies**		

CCDC22	Proteintech	Cat# 16636-1-AP
CCDC93	LS Bio	Cat# C336997
CCDC93	Proteintech	Cat# 20861-1-AP
EEA1	BD Biosciences	Cat# 610457
FAM21	Gift from Dan Billadeau	N/A
GFP	Roche	Cat# 11814460001
Integrin α5	BD Biosciences	Cat# 555651
Integrin α5	Abcam	Cat# EPR7854
Integrin β1	BD Biosciences	Cat# 610467
LAMP1	Developmental Studies Hybridoma Bank	Cat# H4A3
VPS26A	Abcam	Cat# ab137447
VPS26C	Merck Millipore	Cat# ABN87
VPS29	Abcam	Cat# ab98929
VPS29	Santa Cruz	Cat# 398874
VPS35	Abcam	Cat# ab157220
VPS35	Abcam	Cat# ab97545
VPS35	Abcam	Cat# ab10099
VPS35L	Abcam	Cat# ab97889
VPS35L	Pierce	Cat# PA5-28553
WASH1	Gift from Dan Billadeau	N/A
TBC1D5	Abcam	Cat# 203896
ANKRD27	Abcam	Cat# ab108216
GFP	Roche	Cat# 11814460001
N-cadherin	Cell Signaling	Cat# 14215
N-cadherin	Cell Signaling	Cat# 13116
β actin	Sigma	Cat# A1978
COMMD1	Proteintech	Cat# 11938-1-AP
COMMD1	Sigma	Cat# WH0150684M1
COMMD2	Millipore Sigma	Cat# HPA044190
COMMD3	Abcam	Cat# ab176583
COMMD4	Abcam	Cat# ab115169
COMMD5	Proteintech	Cat# 67043-1-Ig
COMMD7	Abcam	Cat# ab96091
COMMD7	GeneTex	Cat# GTX112076
COMMD8	Proteintech	Cat# 25237-1-AP
COMMD8	Santa Cruz	Cat# sc-3973869
COMMD9	Abcam	Cat# Ab121303
COMMD10	Santa Cruz	Cat# sc-398798
COMMD10	GeneTex	Cat# GTX121488
HA-tag	Insight Biotechnology	Cat# A02040
Strep-tag	GeneTex	Cat# GTX128061
6x His-tag	Abcam	Cat# Ab18184
Myc-tag	GeneTex	Cat# GTX115046
FLAG tag	Sigma	Cat# F3165
SNAP-tag	GeneTex	Cat# GTX54523
Alexa Fluor 680 anti-mouse IgG	Life Technologies	Cat# A10038
Alexa Fluor 800 anti-rabbit IgG	Life Technologies	Cat# W10824
Goat anti-rabbit IgG	Life Technologies	Cat# AB2533967
Alexa Fluor 488 anti-mouse IgG	Life Technologies	Cat# A21202
Alexa Fluor 568 anti-mouse IgG	Life Technologies	Cat# A10037
Alexa Fluor 647 anti-mouse IgG	Life Technologies	Cat# A31571
Alexa Fluor 488 anti-rabbit IgG	Life Technologies	Cat# A21206
Alexa Fluor 568 anti-rabbit IgG	Life Technologies	Cat# A10042
Alexa Fluor 647 anti-rabbit IgG	Life Technologies	Cat# A31573
Alexa Fluor 488 anti-goat IgG	Life Technologies	Cat# A11055
Alexa Fluor 568 anti-goat IgG	Life Technologies	Cat# A11057
Alexa Fluor 647 anti-goat IgG	Life Technologies	Cat# A21447

**Bacterial and virus strains**		

*E. coli* DH5α	Invitrogen	Cat# 18265017
BL21 (DE3)	New England Biolabs	Cat# C2527H
DH10EMBacY	ThermoFisher	Cat# 10361012
NEB 5-alpha	New England Biolabs	Cat# C2987H

**Experimental models: Cell lines**		

HeLa	ATCC	Cat# CCL-2
HEK293T	ATCC	Cat# CRL-3216
RPE1	ATCC	Cat# CRL-4000
*Spodoptera frugiperda* Sf21	ThermoFisher	Cat# 11497013
eHap	Horizon Discovery	Cat# C669
VPS35 knock-out HeLa cells	^97^	N/A
VPS29 knock-out HeLa cells	This study	N/A
VPS35L knock-out HeLa cells	This study	N/A
COMMD1 knock-out eHAP cells	This study	N/A
COMMD2 knock-out eHAP cells	This study	N/A
COMMD3 knock-out eHAP cells	This study	N/A
COMMD4 knock-out eHAP cells	This study	N/A
COMMD5 knock-out eHAP cells	This study	N/A
COMMD6 knock-out eHAP cells	This study	N/A
COMMD7 knock-out eHAP cells	This study	N/A
COMMD8 knock-out eHAP cells	This study	N/A
COMMD9 knock-out eHAP cells	This study	N/A
COMMD10 knock-out eHAP cells	This study	N/A

**Chemicals and peptides**		

Benzamidine hydrochloride hydrate	Sigma Aldrich	Cat# B6506
Deoxyribonuclease I (DNase I)	Sigma Aldrich	Cat# DN25
Talon® resin	Clontech	Cat# 635503
Glutathione Sepharose 4B	GE Healthcare	Cat# GEHE17-0756-0
Isopropyl β-D-1-thiogalactopyranoside	Bioline	Cat# BIO-37036
Triton X-100	Sigma Aldrich	Cat# X100-500ML
VPS35L (16-38)	Genscript	N/A
VPS35L (27-38)	Genscript	N/A
Sf-900 II SFM media	ThermoFisher Scientific	Cat# 10902088
X-tremeGENE HP DNA Transfection Reagent	Sigma-Aldrich	Cat# 6366244001
Graphene oxide	Sigma-Aldrich	Cat# 763705
DMEM	Sigma-Aldrich	Cat# D5796
25 kDa PEI	Polysciences	Cat# 23966-2
Sulfo-NHS-SS Biotin	ThermoFisher	Cat# 21217
Fluoromount-G	Invitrogen	Cat# 004958-02

**Critical commercial assays**		

QIAprep Spin Miniprep Kit	QIAGEN	Cat# 27106
NEBuilder HiFi DNA Assembly System (NEB)	New England Biolabs	Cat# E5520S
TMT seven-plex	ThermoFisher	Cat# 90061

**Oligonucleotides**		

Primers used in this study, see Table S3	This paper	N/A
gRNA targeting VPS29 exon 2 GGCAAACTGTTGCACCGGTG	This paper	N/A
gRNA targeting VPS29 exon 3 GGACATCAAGTTATTCCATG	This paper	N/A
gRNA targeting VPS35L exon 1 GCAGACGCCATGCTTGTGAG	This paper	N/A
gRNA targeting COMMD1 CGCTGGAATAGCGGGCTTCGGGG	This paper	N/A
gRNA targeting COMMD2 GCGGCGCCTTCGTAGATTTTTGG	This paper	N/A
gRNA targeting COMMD3 GCCGAGCGGCTTACCTAACACGG	This paper	N/A
gRNA targeting COMMD4 AGCGGGATACTCACCAAACTTGG	This paper	N/A
gRNA targeting COMMD5 AGCAATGGCCCGGCTACTAGGGG	This paper	N/A
gRNA targeting COMMD6 CGTTGCAGTGATGCTAAAAGTGG	This paper	N/A
gRNA targeting COMMD7 ACCCTTGCTCGATGGGCCATAGG	This paper	N/A
gRNA targeting COMMD8 TACCTTATTCGCTGCTTCCAAGG	This paper	N/A
gRNA targeting COMMD9 CACGCCTGGTCGATCTGGACTGG	This paper	N/A
gRNA targeting COMMD10 AGAATCCGAGTGAGCAACCGTGG	This paper	N/A

**Recombinant DNA**		

pACEBAC1	NovoPro	Cat# V011691#
MultiBAC pKL vector	Gift from Prof. Imre Berger	N/A
pbiG1a	Gift from Dr. Andrew Carter, MRC-LMB, UK.^98^	N/A
pbiG2b	Gift from Dr. Andrew Carter, MRC-LMB, UK.^98^	N/A
pbiG2d	Gift from Dr. Andrew Carter, MRC-LMB, UK.^98^	N/A
pbiG2e	Gift from Dr. Andrew Carter, MRC-LMB, UK.^98^	N/A
pbiG1a Strep-VPS26C, VPS35L, VPS29-His (Retriever)	This study	N/A
pbiG2bde COMMD1, myc-COMMD2, SNAP-COMMD3, His-COMMD4, HA-COMMD5, HA-COMMD6, V5-COMMD7, COMMD8, FLAG-COMMD9, COMMD10, CCDC22, CCDC93-Strep (CCC complex)	This study	N/A
pbiG1A VPS35, Strep-VPS26A, VPS29-His (Retromer)	This study	N/A
pACEBac1 His-TBC1D5	This study	N/A
pST39 Commd1-FLAG, Commd2, Commd6, Commd4, Commd7, Commd8, Commd9, Commd3, Commd5-Strep, Commd10-His.	This study	N/A
pST39 Commd1-His, Commd2, Commd6, Commd4, Commd7, Commd8, Commd9, Commd3, Commd5, Commd10.	This study	N/A
pST39 Commd1, Commd2-His, Commd6, Commd4, Commd7, Commd8, Commd9, Commd3, Commd5, Commd10.	This study	N/A
pST39 Commd1, Commd2, Commd6, Commd4, Commd7, Commd8, Commd9, Commd3, Commd5-His, Commd10.	This study	N/A
pRSF-Duet-1 COMMD1-His, COMMD6, COMMD4, COMMD8	This study	N/A
pRSF-Duet-1 COMMD2-His, COMMD3, COMMD4, COMMD8	This study	N/A
pST39 COMMD7, COMMD9, COMMD5, COMMD10-His	This study	N/A
pSpCas9(BB)-2A-GFP	Addgene	PX458
pUMVC3	Addgene	8449
pCMV-VSV-G	Addgene	8454
pEGFP-N1 VPS35L	This study	N/A
pLVX VPS35L	This study	N/A
pGEX4T-2 VPS29	^44^	N/A
pGEX6P-1 DENND10	This study	N/A
pRSF CCDC22 (325–485) + CCDC93 (310–488) CC1 and CC2	This study	N/A
DENND10-GFP	This study	N/A
GFP-CCDC22	McNally et al.^6^	N/A
GFP-CCDC93	McNally et al.^6^	N/A
mCherry RidL	Gift from Da Jia.^47^	N/A
mCherry RidL(1–200)	Gift from Da Jia.^47^	N/A

**Deposited data**		

Commd5-10-7-9 complex (crystal structure)	RCSB Protein DataBank (this study)	PDB: 8ESD
VPS29-VPS35L peptide complex (crystal structure)	RCSB Protein DataBank (this study)	PDB: 8ESE
CCC complex (cryoEM structure; RELION map)	RCSB Protein DataBank (this study)	PDB: 8F2R
CCC complex (cryoEM structure; CryoSPARC map)	RCSB Protein DataBank (this study)	PDB: 8F2U
CCC complex (cryoEM structure; RELION map)	Electron Microscopy DataBank (this study)	EMDB ID: EMD-28827
CCC complex (cryoEM structure; CryoSPARC map)	Electron Microscopy DataBank (this study)	EMDB ID: EMD-28825
Commd9 COMM domain complex (crystal structure)	RCSB Protein DataBank^42^	PDB: 6BP6
Commd9 HN domain (crystal structure)	RCSB Protein DataBank^42^	PDB: 4OE9
Commd10 HN domain (crystal structure)	This study	N/A
Commd1 human protein sequence	NCBI	Q8N668
Commd2 human protein sequence	NCBI	Q86X83
Commd3 human protein sequence	NCBI	Q9UBI1
Commd4 human protein sequence	NCBI	Q9H0A8
Commd5 human protein sequence	NCBI	Q9GZQ3
Commd6 human protein sequence	NCBI	Q7Z4G1
Commd7 human protein sequence	NCBI	Q86VX2
Commd8 human protein sequence	NCBI	Q9NX08
Commd9 human protein sequence	NCBI	Q9P000
Commd10 human protein sequence	NCBI	Q9Y6G5
CCDC22 human protein sequence	NCBI	O60826
CCDC93 human protein sequence	NCBI	Q567U6
VPS35L human protein sequence	NCBI	Q7Z3J2
VPS26C human protein sequence	NCBI	O14972
VPS29 human protein sequence	NCBI	Q9UBQ0
DENND10/FAM45A human protein sequence	NCBI	Q8TCE6
COMMD1-10+ CCDC22 (1–223) + CCDC93 (1–300) (AlphaFold2 Multimer prediction)	Model Archive (https://www.modelarchive.org)	ma-iplv4
CCDC22+ CCDC93 (AlphaFold2 Multimer prediction)	Model Archive (https://www.modelarchive.org)	ma-9nv72
VPS35L + VPS26C+ VPS29 (AlphaFold2 Multimer prediction)	Model Archive (https://www.modelarchive.org)	ma-3cag5
VPS35L + VPS26C+ VPS29+ CCDC22 (1–115) + CCDC22 (386–627) + CCDC93 (378–631) (AlphaFold2 Multimer prediction)	Model Archive (https://www.modelarchive.org)	ma-4097m
Combined models 1 + 2 + 3 (AlphaFold2 Multimer prediction)	Model Archive (https://www.modelarchive.org)	ma-ri7tb
COMMD1-10 *Danio rerio* (AlphaFold2 Multimer prediction)	Model Archive (https://www.modelarchive.org)	ma-99f82
COMMD1-10 *Salpingoeca rosetta* (AlphaFold2 Multimer prediction)	Model Archive (https://www.modelarchive.org)	ma-xfc84
DENND1B in complex with Rab35 (crystal structure)	RCSB Protein DataBank^70^	PDB: 3TW8

**Software and algorithms**		

XDS	^99^	http://xds.mpimf-heidelberg.mpg.de/
AIMLESS	^100^	http://www.ccp4.ac.uk/html/aimless.html
Phaser	^101^	http://www.phaser.cimr.cam.ac.uk/index.php/Phaser_Crystallographic_Software
Phenix	^102^	https://www.phenix-online.org/
Coot	^103^^,^^104^	https://www2.mrc-lmb.cam.ac.uk/personal/pemsley/coot/
Molprobity	^105^	http://molprobity.biochem.duke.edu
Pymol	Schrodinger, USA.	https://pymol.org/2/
ChimeraX	^106^	https://www.rbvi.ucsf.edu/chimerax/
iSOLDE	^107^	https://isolde.cimr.cam.ac.uk
RELION 4.0	^108^	https://relion.readthedocs.io/en/release-4.0/
Topaz	^109^	https://cb.csail.mit.edu/cb/topaz/
CryoSPARC V3.3.1	^110^	https://cryosparc.com
AlphaFold2 Multimer	^54^^,^^55^	https://github.com/deepmind/alphafold
ColabFold	^56^	https://github.com/sokrypton/ColabFold
MAFFT L-INS-i (v7.505)	^111^	https://mafft.cbrc.jp/alignment/software/
IQTree2.1.3	^112^	http://www.iqtree.org
Consurf	^113^	https://consurf.tau.ac.il/consurf_index.php

**Other**		

Superose6 Increase10/300 GL column	Cytiva	Cat# 29091596
HiLoad™ Superdex75 PG column	Cytiva	Cat# 28989333
Mono Q 5/50 GL column	Cytiva	Cat# 17516601
C-Flat 1.2/1.3 grids	C-Flat	N/A
Quantifoil 1.2/1.3 Copper 300 mesh grids	Quantifoil	N/A
Anti-FLAG M2 affinity gel	Sigma Aldrich	Cat# A2220
GFP-trap beads	Chromotek	Cat# gta-20
mCherry-trap beads	Chromotek	Cat# rta-20
Streptavidin Sepharose beads	Cytiva	Cat# 90100484

### Resource availability

#### Lead contact

Further information and requests for resources and reagents should be directed and will be fulfilled by the lead contact Prof. Peter Cullen (pete.cullen@bristol.ac.uk).

#### Materials availability

Plasmids generated in this study are available from the lead contact with a completed Materials Transfer Agreement.

### Experimental model and subject details

*E. coli* BL21 (DE3) cells were used for the overexpression of native recombinant proteins. Cells were grown at 37°C and protein expression was induced with 0.8 mM isopropylthio-β-galactoside (IPTG) before the temperature was reduced to 21°C and cultures were allowed to grow for 18 h. HeLa, HEK293T and RPE1 cells were maintained in DMEM (D5796; Sigma-Aldrich) plus 10% fetal calf serum (F7524; Sigma-Aldrich) under standard conditions. These cell lines were obtained from America Type Culture Collection (ATCC). Parental and stable cells lines were negative for mycoplasma by DAPI staining. *Spodoptera frugiperda* Sf21 cells (Cat no. 11497013, ThermoFisher Scientific) for baculoviral expression of recombinant proteins in insect cells were grown at 26°C in Sf-900 II SFM media (Cat no. 10902088, ThermoFisher Scientific).

### Method details

#### Cloning with biGBac plasmids

##### Retriever and Retromer

Genes for human Retriever and Retromer were codon optimized for *S. frugiperda* and synthesized by Twist Biosciences (San Francisco, CA). Codon optimised genes were cloned into pACEBAC1. Retromer and Retriever were assembled through Gibson assembly into pBIG1A (empty pBIG plasmids were gifts from Dr Andrew Carter, MRC-LMB, Cambridge, UK).^98^ For TBC1D5 expression, the coding region of TBC1D5 was subcloned from pEGFP-C1 TBC1D5 into pACEBac1. CCC complex: Genes for human CCC complex expression were codon optimized for *S. frugiperda* while avoiding the introduction of *Pme*I, *Swa*I, *Bam*H1 and *Hind*III restriction sites and synthesized by Twist Biosciences (San Francisco, CA). Codon optimised sequences were cloned into the MultiBAC pKL transfer plasmid at the *Bam*HI and *Hind*III sites within the multiple cloning site. The CCC complex genes were assembled using the biGBac cloning strategy.^52^^,^^53^ Plasmid DNA was prepared using a QIAprep Spin Miniprep Kit according to the manufacturer’s protocol (Cat no. 27106, Qiagen).

### Bacmid purification

pACEBac1 or pBIG vectors were transformed into DH10EMBacY competent cells which contain a modified baculoviral genome.^52^ Transformations were left to recover overnight before being plated onto agar plates containing 50 μg/mL kanamycin, 10 μg/mL tetracycline, 7 μg/mL gentamycin, 40 μg/mL Isopropyl β-*d*-1-thiogalactopyranoside (IPTG) and 100 μg/mL Blue-Gal (Cat no. 15519028, ThermoFisher Scientific). The multigene transfer vector integrates with the baculoviral genome via Tn7 transposition. White colonies were grown overnight in 2 mL of LB supplemented with 50 μg/mL kanamycin, 10 μg/mL tetracycline, 7 μg/mL gentamycin. Bacmid DNA was prepared using buffers from a QIAprep Spin Miniprep Kit (Cat no. 27106, Qiagen) according to the MultiBac protocol.

#### Baculovirus generation

Sf21 cells were seeded at 1x10^6^ cells/well in a 6-well plate in a total volume of 3 mL of Sf-900 II SFM media (Cat no. 10902088, ThermoFisher Scientific). Bacmid DNA was transfected into Sf21 cells using X-tremeGENE HP DNA Transfection Reagent (Cat no. 6366244001, Sigma-Aldrich) according to the manufacturer’s protocol and incubated at 26°C for 72 h. The media from the transfected culture was used to infect a 25 mL suspension culture of Sf21 cells at 1x10^6^ cells/ml. At 48 h post proliferation arrest the V1 generation of virus was harvested by pelleting the cells at 2000 rpm for 10 min and collecting the supernatant. To amplify the infectivity of the virus, V1 was added to a culture of Sf21 cells and supernatant harvested - termed V2. All viruses were stored at 4°C in the dark.

#### Protein expression in insect cells

For protein expression, the V1 or V2 virus were used to infect suspension cultures of Sf21 insect cells in Sf-900 II SFM media (Cat no. 10902088, ThermoFisher Scientific). Cells were seeded at 0.6x10^6^ cells/ml in 2 L Erlenmeyer shaker flasks in a total volume of 600 mL. At a density of 1x10^6^ cells/ml, 6 mL of V1 or V2 was added to the culture. At 48 h post proliferation arrest cells were harvested by centrifugation at 2000 rpm for 10 min. Cell pellets were either immediately used for protein purification or stored at −20°C.

#### Retriever and TBC1D5 purification

The insect cell pellets were resuspended in lysis buffer (25 mM HEPES pH 8.0, 300 mM NaCl, 2 mM β-mercaptoethanol, EDTA-free protease inhibitor tablets (A32965, Pierce)) and lysed on ice using a 130-Watt Ultrasonic Processor (UY-04714-51, Cole-Parmer) for a total of 2 min 30 s using a 10 s on 30 s off cycle. Lysates were cleared by centrifugation at 4°C for 30 min at 18,000 x g. His TALON resin was used to purify his-tagged proteins. Purification was performed at 4°C. TALON resin was equilibrated with lysis buffer (25 mM HEPES pH 8.0, 300 mM NaCl, 2 mM β-mercaptoethanol). Cleared cell lysate was then added to the column and allowed to flow through the TALON resin. Once the lysate had completely flowed through the column, the column was thoroughly washed in 10x CV lysis buffer, followed by 10x CV wash buffer (25 mM HEPES pH 8.0, 300 mM NaCl, 2 mM β-mercaptoethanol and 20 mM imidazole). His-tag proteins were eluted from the column by elution buffer (25 mM HEPES pH 8.0, 300 mM NaCl, 2 mM β-mercaptoethanol and 200 mM imidazole). Size exclusion chromatography (SEC) was performed at 4°C using an ÄKTA prime and purifier system (GE Healthcare). A Superdex200 size exclusion column 10/300 GL (GE healthcare, catalog number 28990944) was equilibrated in SEC buffer (25 mM HEPES pH 8.0, 300 mM NaCl, 2 mM β-mercaptoethanol). Protein was injected onto the column and 0.5mL fractions were collected.

#### Retromer purification

Retromer was purified using the same method as Retriever/TBC1D5 but using different buffers – lysis buffer: 25 mM HEPES pH7.5, 150 mM NaCl, 2 mM β-mercaptoethanol, EDTA-free protease inhibitor tablets (A32965, Pierce); wash buffer: 25 mM HEPES pH 7.5, 150 mM NaCl, 2 mM β-mercaptoethanol and 20 mM imidazole; elution buffer: 25 mM HEPES pH 7.5, 150 mM NaCl, 2 mM β-mercaptoethanol and 200 mM imidazole; SEC buffer: 25 mM HEPES pH 7.5, 150 mM NaCl, 2 mM β-mercaptoethanol.

#### CCC complex purification

Insect cell pellets were resuspended in 5x volume of lysis buffer (50 mM HEPES pH7.2, 150 mM NaCl, 2 mM β-mercaptoethanol, 0.1% Triton X-100 with EDTA-free protease inhibitor tablets (A32965, Pierce). Lysates were sonicated on ice using a 130-Watt Ultrasonic Processor (UY-04714-51, Cole-Parmer) for a total of 2 min 30 s using a 10 s on 30 s off cycle. Lysates were cleared by centrifugation at 20,000 rpm for 25 min at 4°C. Cleared lysates were loaded onto a Econo-Pac Chromatography Column (Cat no. 7321010, Bio-Rad) packed with 1 mL Streptactin resin (2–1201, IBA Lifesciences) pre-equilibrated in lysis buffer. The column was washed with 2 × 25 mL lysis buffer and bound protein eluted using 5 × 1 mL lysis buffer plus 2.5 mM desthiobiotin. A subset of protein containing fractions were crosslinked with 1 mM BS3 (11841245, ThermoFisher Scientific) for 2 h at 4°C. The reaction was quenched using 1 M Tris, pH 7.5 at a final concentration of 50 mM. Crosslinked and non-crosslinked protein containing fractions were gel filtered using a Superose 6 10/300 GL size exclusion column (Cat no. 29091596, GE Healthcare) attached to an ÄKTA pure chromatography system (GE Healthcare) pre-equilibrated in buffer containing 50 mM HEPES pH7.2, 150 mM NaCl, 2 mM β-mercaptoethanol, 0.01% (v/v) Triton X-100. Fractions of 500 μL were collected and analyzed. All purifications steps were performed at 4°C and samples kept on ice.

#### Native PAGE

Samples were prepared in a 1X dilution of Novex Tris-Glycine Native Sample Buffer (2X) (LC2673, ThermoFisher Scientific). Samples were separated by native polyacrylamide gel electrophoresis (native-PAGE) on a Novex WedgeWell 8 to 16% Tris-Glycine mini protein gel (XP08162BOX, Invitrogen). A 1X running buffer was prepared using (10X) Novex Tris-Glycine Native Running Buffer and used to fill the chamber of Invitrogen Mini Gel Tanks (A25977, Invitrogen). Typically, 20 μg of protein was loaded per well along with 4 μL of NativeMark Unstained Protein Standard (LC0725, ThermoFisher Scientific) in one lane as a molecular weight marker. Following PAGE, the gel was washed in ddH_2_0 for 5 min. To visualise the proteins the gel was immersed in Coomassie stain, made with 0.1% Coomassie Brilliant Blue R-250 (B7920, Sigma-Aldrich), 40% methanol, 10% acetic acid and filtered through a Whatman No. 1 filter. The gel submerged in Coomassie stain was heated in a microwave for 30 s and allowed to incubate for 2 min with gentle agitation. The stain was removed and rinsed with ddH_2_0 before immersing in de-stain (20% methanol (v/v), 10% acetic acid (v/v) in ddH_2_0) and microwaving for 30 s. The gel was incubated in de-staining solution until bands could be distinguished from background stain. Gels were visualised with an Odyssey infrared imaging system (LI-COR Biosciences).

#### Negative stain EM

5 μg of Retriever was placed onto carbon-coated pioloform copper-mesh grids and incubated for 1 min. After the incubation, the excess protein solution was blotted and the grid was washed quickly in 4 μL 3% uranyl acetate, blotted again and then incubated with 4 μL of 3% uranyl acetate for 1 min. After the uranyl acetate incubation, the grids were blotted, washed a third time in uranyl acetate before blotting dry and left to air dry. Images were recorded on a 200-kV Tecnai F20 microscope (FEI) equipped with a FEI Ceta 4k x 4k charge-coupled device camera at 68,000 magnification corresponding to a pixel size of 1.63 Å/pixel. A total of 14,000 particles from 280 images were picked and reference free two-dimensional classification was performed with RELION 3.2.

#### Graphene oxide coating of EM grids

Graphene oxide coated grids were prepared the day before use. Quantifoil 1.2/1.3 Copper 300 mesh grids were glow discharged for 1 min using an Edwards S150B Sputter coater discharger at power level 7, 40 mA. Graphene oxide (Sigma, 763705, 2 mg/mL in H_2_0) was freshly diluted 1/10 to 0.2 mg/mL in MilliQ water. The diluted graphene oxide solution was span at 600 x g until the visible flakes pelleted. 3 μL of span graphene oxide solution, taken from the top, was applied to the glow-discharged grids and incubated for 1 min. After incubation, grids were blotted with Whatman No.1 filter paper and washed/blotted twice with 20 μL of MilliQ H_2_O and a final wash with 20 μL of MilliQ H_2_0 was applied to the bottom of the grid. Grids were air-dried overnight.

#### Cryo-EM grid preparation

4 μL of ∼0.1 mg/mL purified Retriever was vitrified in ethane-propane on glow-discharged C-Flat 1.2/1.3 grids using a FEI Vitrobot. Grids were screened for suitable ice using a 200kV Talos Arctica equipped with energy filter and *a* Gatan K2 direct electron detector. 3 μL of ∼0.2 mg/mL purified, cross-linked human CCC complex was applied onto a graphene oxide coated Quantifoil 1.2/1.3 Copper 300 mesh grid. The grids were not glow discharged before sample application. Sample was incubated on the grid for 30 s prior to blotting (3–3.5 s at blot force −15) and vitrification in liquid ethane using a FEI Vitrobot Mark IV. Grids were screened for suitable ice and particle distribution using a 200 kV ThermoFisher Glacios equipped with Falcon III direct electron detector.

#### Cryo-EM data collection

Data collection of the Retriever complex was performed on at 200 kV Talos Arctica equipped with energy filter and Gatan K2 detector. EPU software (ThermoFisher Scientific) was used for automated data acquisition. Data was collected in super-resolution mode with a virtual pixel size of 0.525 Å per pixel. 4,862 movies were collected, with a dose rate of 61.7 e−/Å^2^. Data collection of the CCC complex was performed on a 300 kV ThermoFisher Scientific Titan Krios transmission electron microscope with a Falcon IV direct electron detector. EPU software (Thermo Fisher Scientific) was used for automated data acquisition. Data was collected from 2 independently prepared grids, in 2 separate sessions. The Falcon IV detector was used in counted mode with a pixel size of 1.084 Å per pixel for both data collections. The first dataset consisting of 2871 movies was collected with a total dose 43.55 e−/Å^2^ over a total exposure time of 9.01 s. The second dataset, consisting of 2779 movies was collected with a total dose of 42.73 e−/Å^2^ over a total exposure time of 9.01 s. A range of defocus values (−1.2, −1.4, −1.6, −1.8, −2.0 μm) were used for collection of both datasets. Movies on the Titan Krios were collected in EER format.

#### Cryo-EM data processing Retriever

Movies were imported into RELION 4.0.^108^ Using the motion-correction program implemented within RELION, movies were dose-weighted, drift-corrected, gain-corrected and summed into single micrographs. A binning factor of 2 was used during motion correction, resulting in motion-corrected micrographs with a pixel size of 1.05 Å. CTFFIND-4.1, integrated within RELION, was used to estimate the contrast transfer function (CTF) parameters for the motion-corrected micrographs. Micrographs with high astigmatism values or crystalline ice were removed from the dataset.

Retriever particles were manually picked, with a particle diameter of 180 Å until ∼3000 particles had been picked. The manually picked particles were used to train Topaz^109^ which is implemented within RELION. Particles were then auto picked using the Topaz trained network. Particles with a figure-of-merit (FOM) value above −3 were extracted using a box size of 240 pixels. We then performed 2 rounds of reference-free 2D classification. Following 2D classification, we only discarded classes that did not look like they contained protein particles (*e.g.* ice crystals). We kept all classes that looked to contain protein particles as we did not want to lose rare orientations of the particles during the 2D classification steps. 252,548 particle stacks were imported into CryoSPARC V3.3.1.^110^ Initial 3D reconstructions were generated using an *ab initio* job with 4 classes. The resulting four 3D initial reconstructions were then used as templates in a heterologous refinement with 4 classes. The map quality of resulting classes from the heterologous refinement were assessed in Chimera. One class, with 119,564 particles, was selected for further processing. This class was refined using homogeneous refinement followed by NU (Non-uniform) refinement. Gold-standard Fourier Shell Correlations (FSCs) and directional FSC plots and measurements of sphericity (calculated using the online 3DFSC tool) indicate that the final map has an overall resolution of ∼4.2 Å. However, this is an overestimation of the resolution of the map, as it was very clear from the reference-free 2D classification classes and 3D refinement jobs that Retriever particles displayed preferred orientation. The final CryoSPARC refined map does not contain particles observed from all angles, has a large deviation from the directional FSC and displays low sphericity (0.715 out of 1) (Methods S1). The map is therefore insufficient for model building and refinement.

We tried unsuccessfully to overcome these preferential orientation issues by changing the sample prep, including but not limited to; changing buffer composition and pH; addition of detergents; protein concentration; grid type (Quantifoil copper/gold, lacey, hole size); grid support (carbon, graphene oxide); freezing conditions (blotting time, humidity, plasma cleaning time, incubation time, blotting equipment (FEI Vitrobot, Leica EM GP)). We also collected a tilted dataset but data processing with this dataset was unsuccessful (data not shown). We also tried several methods of data processing in RELION/CryoSPARC to recover less frequent orientations. However, these were unsuccessful at improving the distribution of views and the overall final map (data not shown).

#### Cryo-EM data processing the CCC complex

Data processing of the CCC complex micrographs was first performed in CryoSPARC V3.3.1. The two movie datasets from the two independent grids were processed independently until indicated. Movies were imported into CryoSPARC and were processed using CryoSPARC’s patch motion correction. CTF estimation was achieved using CryoSPARC’s patch CTF. Particles were initially picked using the blob picker tool with a minimum particle diameter of 100 Å and a maximum particle diameter of 180 Å. Picked particles were extracted and underwent several rounds of reference-free 2D classification. Good 2D classes were selected and used as templates for a further round of particle picking using CryoSPARC’s template picker tool. These newly picked particles were extracted and underwent several rounds of 2D classification and class selection to discard any ‘bad particles’. The best 2D classes were then used to generate *ab initio* reconstructions of the complex. For the first dataset, consisting of 2,779 micrographs, 198,705 particles were used to generate 2 *ab inito* reconstructions. For the second dataset, consisting of 2,872 micrographs, 151,195 particles were used to generate 4 *ab initio* models. The *ab initio* models from each respective dataset were then used as templates in a hetero refinement job using the same particles as the *ab inito* job. Following hetero refinement, maps were opened in chimera and the best 3D classes were selected for further refinement using homo refinement and in the case of the first dataset, a further step of NU refinement. The resulting 2 refined reconstructions from the 2 independent datasets (consisting of 138,424 and 55,894 particles respectively) were then used to create templates for a further round of particle picking, using the create template tool in CryoSPARC. Particles were picked using the templates created from their respective dataset and extracted. Particles picked and extracted from the second dataset (374,899) were classified using 2D classification and good classes (209,507 particles) were used to train Topaz. This Topaz trained model was then used to pick particles in RELION4.0 (see below). The extracted particles from each independent dataset were combined to give a total of 2,063,629 particles. Good classes of particles were selected following several rounds of 2D classification. 4 *ab initio* models were generated from 333,394 particles and these models were used as templates for a subsequent hetero refinement. The classes were opened in Chimera and the best class consisting of 153,104 particles was chosen for homo refinement followed by NU refinement to give a final reconstruction with an overall resolution of 3.1 Å. Some regions of the map were less well resolved than the central core domain (which was very well resolved in the CryoSPARC reconstruction) probably due to flexibility of these domains relative to the core. To better resolve the flexible regions of the map, we processed the data in RELION 4.0. The two independent datasets were processed analogously in RELION but kept independent until stated. Micrographs were imported into RELION, dose-weighted, drift-corrected, gain-corrected and summed into single micrographs using the motion-correction program implemented within RELION. CTF estimation was done using CTFFIND-4.1 within RELION. Particles were auto picked using the Topaz implementation within RELION, using the Topaz trained model from the CryoSPARC processing (see above). Particles with a figure of merit above −1 were extracted with a box size of 264 and then binned by a factor of 4. Particles underwent one round of 2D classification, from which the best classes were selected and used in a 3D classification job with 6 classes. The final CryoSPARC map of the CCC complex (described earlier) was binned, imported into RELION and used as an initial model in these 3D classification jobs. A T parameter of 4, 40 iterations and angular sampling of 7.5° were used for the 3D classifications. 3D classes were opened in Chimera and the best 3D class was selected. Particles in this class were re-extracted without binning and then the selected particles from each independent dataset were combined to give a total of 166,533 particles which were then refined to 4.2 Å. The particles then were subjected to one consecutive rounds of CTF refinement (beam tilt, anisotropic magnification and defocus), followed by Bayesian polishing^114^ and 3D refinement. This further improved the global resolution of the map to 4.1 Å. To improve the resolution of the densities of the flexible HN domains, the angular assignments from the latest refinement were used for a round of alignment-free 3D classification, with a T parameter of 16. The best 3D class (20,034 particles), which contained densities for all the HN domains as well as the CH domain of CCDC93, was selected for a further round of 3D refinement, resulting in a 3.8 Å reconstruction, which was further improved to 3.5 Å following a final round of CTF refinement and 3D refinement.

#### Model building and refinement

The AlphaFold2 model of the COMMD1-10, CCDC22 (1–260), CCDC93 (1–306) complex (see below) was docked into the sharpened RELION 4.0^108^ map using Chimera X.^106^ The docked model was then passed through PHENIX^102^ real-space refinement to correct Ramachandran outliers and bond angles. To improve the stereochemistry and clash score this model was refined with the Chimera X plugin iSOLDE^107^ and then subjected to several rounds of further refinement and rebuilding using a combination of PHENIX,^102^ COOT^103^ and iSOLDE^107^ resulting in a final model with excellent refinement statistics and stereochemistry based on Molprobity scores. All images were rendered using ChimeraX.^106^

#### Molecular biology for *E. coli* expression

A series of gene cassettes codon optimised for bacterial expression were synthesised by the Gene Universal Corporation (USA). These were then progressively cloned into the pST39 vector to allow co-expression of all ten human COMMD family members. Briefly, gene cassettes were as follows cassette 1 (*Xba*I): COMMD1, COMMD2, COMMD6; Cassette 2 (*Hind*III): COMMD4; Cassette 3 (*Eco*RV): COMMD7, COMMD8, COMMD9; Cassette 4 (*Nru*I): COMMD3, COMMD5-Strep, COMMD10-His. Each protein contained a 5′ ribosomal binding site and a 3′ stop codon and were expressed off a single T7 promoter allowing the simultaneous expression of each of the ten COMMD proteins individually. The order of COMMD proteins was determined due to the internal restriction site and the required restriction sites for vector linearisation and cloning. A series of four vectors were then developed from this template to only have a singular His tag on COMMD1, 2 and 5. Subsequent pRSFduet co-expression vectors encoding four proteins from each of the subcomplex es Subcomplex A (COMMD1His-6-4-8) Subcomplex B (Commd2his-3-4-8) and subcomplex C (COMMD5-SBP-7-9-10-His), were synthetically generated by Gene Universal using the same codon optimised gene sequences. DENND10 was synthesized and codon optimized for *E.coli* expression by geneuniversal and subcloned into a pGEX6P-1 vector (BamHI). CCDC22 (325–485) and CCDC93 (310–488) were synthesized by geneuniversal and subcloned into a pRSFduet vector at BamHI and NdeI, respectively.

#### Protein purification from *E. coli*

The bacterial expression plasmids were transformed into *E. coli* BL21 DE3 competent cells (New England Biolabs) and plated on agar plates containing ampicillin. Clones from this agar plate were collected and grown overnight in 10 mL LB broth, overnight “starter” cultures were expanded into 1 L cultures. Cultures were grown until reaching OD_600_ reached 0.8 and induced with 0.8 mM isopropylthio-β-galactoside (IPTG). Cultures were then cooled to 20°C and allowed to grow for ∼16 h. Cells were harvested by centrifugation at 6000 x g for 5 min at 4°C and the harvested cell pellet was resuspended in lysis buffer (50 mM HEPES pH 7.4, 500 mM NaCl, 5 mM Imidazole, 10% glycerol, 1 mM n-Dodecyl-β-D-Maltopyranoside (DDM), 50 μg/mL benzamidine, 100 units of DNaseI, and 2 mM β-mercaptoethanol). Cells were lysed using sonication and the lysate was clarified by centrifugation at 50,000 x g for 30 min at 4°C. Complexes were purified on a Talon resin (Clontech) gravity column and eluted using 500 mM imidazole in a buffer containing 500 mM NaCl, 10% glycerol, and 2 mM β-mercaptoethanol. Eluted proteins were subsequently passed through a Superdex 200 10/300 column attached to an AKTA Pure system (GE healthcare) in 50 mM HEPES pH 7.4, 500 mM NaCl for crystallisation, and isothermal titration experiments; 50 mM Tris pH 7.4, 500 mM NaCl for mass photometry. For further analysis by MS COMMD complexes isolated using Talon were passed through SEC in 50 mM Tris pH 7.4 and 30 mM NaCl and further purified using a mono Q anion exchange chromatography column with a gradient running from 30 mM NaCl to 500 mM NaCl. Wild type and mutant complexes of CCDC22 (325–485) and CCDC93 (310–488) contained an amino-terminal decaHis tag on CCDC93 and was co-expressed using the pRSF-duet vector, expression and purification were as above. DENND10 was expressed as above and purified in the same manner except talon resin was substituted for glutathione resin (Clontech).

#### uHPLC-mass spectrometry

COMMD complexes purified by size exclusion chromatography and anion exchange chromatography were trypsinised at a ratio of 1:100 (trypsin:protein) overnight at 37°C and analyzed by uHPLC-MS/MS on an Eksigent, Ekspert nano LC400 uHPLC (SCIEX, Canada) coupled to a Triple TOF 6600 mass spectrometer (SCIEX, Canada) equipped with a duo microelectrospray ion source. 5 μL of each extract was injected onto a 300 μm × 150 mm ChomXP C18 CL 3 μm column (SCIEX, Canada) at 5 μL/min. Linear gradients of 2–25% solvent B over 35 min at 5 μL/min flow rate, followed by a steeper gradient from 25% to 60% solvent B in 15 min were used for peptide elution. The gradient was then extended from 60% solvent B to 80% solvent B in 2 min. Solvent B was held at 80% for 3 min for washing the column and returned to 2% solvent B for equilibration prior to the next sample injection. Solvent A consisted of 0.1% formic acid in water and solvent B contained 0.1% formic acid in acetonitrile. The ion spray voltage was set to 5500V, declustering potential (DP) 90V, curtain gas flow 25, nebuliser gas 1 (GS1) 13, GS2 to 15, interface heater at 150°C and the turbo heater to 150°C. The mass spectrometer acquired 250ms full scan TOF-MS data followed by up to 30 × 50ms full scan product ion data in an Information Dependent Acquisition, IDA, mode. Full scan TOFMS data was acquired over the mass range 350–2000 Da and for product ion MS/MS 100–1600 Da. Ions observed in the TOF-MS scan exceeding a threshold of 100 counts and a charge state of +2 to +5 were set to trigger the acquisition of product ion, MS/MS spectra of the resultant 30 most intense ions. The data was acquired and processed using Analyst TF 1.7 software (ABSCIEX, Canada). Protein identification was carried out using Protein Pilot 5.0 for database searching.

#### Mass photometry

Microscope coverslips were washed and inserted into a Refeyn mass photometry instrument (Refeyn Ltd., UK) in the Center for Microscopy and Microanalysis (CMM). All protein complexes were in a buffer containing 50 mM Tris pH 7.4 and 500 mM NaCl, and all buffers were filtered through a 0.22 μM filter. Calibration was preformed using a mass calibrant purchased from Sigma-Aldrich that contained bovine serum albumin, alcohol dehydrogenase and β-amylase. 6000 frames were collected for each protein and analyzed using the Refeyn provided software. Briefly, movies record light scattering events as proteins interaction with the coverslips and the amount of light scattered is quantified and a histogram. Gaussian distributions were then fitted to each peak to determine the molecular weights.

#### Isothermal titration calorimetry (ITC)

The affinities of VPS29 interaction with the synthetic VPS35L peptides and DENND10 with the CCDC22 (325–485) and CCDC93 (310–488) complex and associated mutants was determined using a Microcal PEAQ instrument (Malvern, UK). Experiments we performed in 100 mM Tris (pH 7.4) and 300 mM NaCl. Native and mutant VPS35L peptides at 600 μM were titrated into 20 μM of VPS29, while 50 μM of DENND10 was titrated into 10 μM of wild type and mutant CCDC22 and CCDC93 complexes. In both cases in 13 x 3.22 μL aliquots were used at a temperature of 25°C. The dissociation constants (*K*_*d*_), enthalpy of binding (Δ*H*) and stoichiometries (N) were obtained after fitting the integrated and normalised data to a single site binding model. The apparent binding free energy (Δ*G*) and entropy (Δ*S*) were calculated from the relationships Δ*G* = RTln(*K*_d_) and Δ*G* = Δ*H* - TΔ*S*. All experiments were performed at least in triplicate to check for reproducibility of the data.

#### SPARSE matrix crystal screening

COMMD subcomplex C was purified and concentrated to ∼8 mg/mL for crystallization screening. Three commercially available SPARSE matrix hanging-drop crystal screens (LMB, PEGRX, JCSG+) were setup using a Mosquito liquid handling robot (TTP LabTech) at 20°C. Numerous crystal conditions were obtained for COMMD subcomplex C and an initial optimisation screen was performed to determine the best crystallisation conditions. The largest crystals were obtained when the protein solution was supplemented with 2 μM crown ether and 10% glycerol and grown in 22% ethanol and 5 mM EDTA. This condition was optimised in a 24 well vapor diffusion plate on glass cover slips by hanging drop, mixing 5 μL protein with 1 μL reservoir solution. Crystals were relatively small with a diamond-shaped morphology (maximum dimensions ∼50 μm). For data collection crystals were cryo-protected in reservoir solution containing 25% glycerol for 10 s prior to flash-cooling in liquid nitrogen. Likewise, VPS29 was concentrated to 16 mg/mL and incubated with 10 mM of the VPS35L peptide (^16^EFASCRLEAVPLEFGDYHPLKPI^38^; Genscript, USA). Using the same SPARSE matrix screens, and crystals were obtained in JCSG+ H3 (0.1 M Bis-tris pH 5.5 and 25% (w/v) PEG3350). 24 well trays using the same solution resulted in large rod-shaped crystals. For data collection crystals were cryo-protected in reservoir solution containing 20% glycerol for 10 s prior to flash-cooling in liquid nitrogen.

#### Crystallographic structure determination

Data was collected at the Australian synchrotron on the MX2 beamline. The data was integrated with XDS^99^ and scaled with AIMLESS^100^ in the CCP4 suite.^115^ Initially the structure was solved by molecular replacement using PHASER^101^ within the PHENIX suite.^102^ For subcomplex C the templates used for molecular replacement searches were the available COMM domain dimer of COMMD9 (PDB: 6BP6),^42^ the HN domain of COMMD9 (PDB: 4OE9)^42^ and the HN domain of COMMD10 (*unpublished*). From analysis of the unit cell volume and Matthews Coefficient using XTRIAGE,^102^ it was estimated that a single copy of the COMMD5-7-9-10 tetramer was present in the asymmetric unit. PHASER was able to successfully place four copies of the COMMD9 COMM domain, one copy of the COMMD9 HN domain and one copy of the COMMD10 HN domain. The resulting model and electron density was sufficient to unambiguously determine the identities of each individual COMM domain. These COMM domains were rebuilt in COOT^103^ allowing clear definition of the core heterotetramer of the COMMD5-7-9-10 COMM domains in the structure. Electron density for the two HN domains positioned by PHASER was relatively poor but they could be identified confidently as belonging to COMMD9 and COMMD10 based on their connectivity to the core COMM domains of these two subunits. Further refinement and rebuilding using a combination of PHENIX, COOT and the ChimeraX plugin, ISOLDE resulted in a final model with excellent refinement statistics and stereochemistry based on Molprobity scores. Despite the quality of the final structure and resulting maps, no electron density was observed for the N-terminal HN domains of either COMMD5 or COMMD7. This is likely due to flexibility in the orientation of these domains. VPS29 bound to the VPS35L peptide was solved using the same method as above, however we used the AlphaFold2 predicted structure of the complex as the input template for molecular replacement.

#### AlphaFold2 modeling

All protein models were generated using AlphaFold2 Multimer^54^^,^^55^ implemented in the ColabFold interface available on the Google Colab platform.^56^ A final Commander model was compiled by combining 3 models each of ∼2000 aa (the current limit of this platform). The models were as follows: COMMD1-10 + CCDC22(1–223), COMMD1-10 + CCDC93(1–300); CCDC22 + CCDC93 + DENND10; VPS29 + VPS26C + VPS35L; VPS35L + CCDC22(1–115; 368–627) + CCDC93(378–630) (see Figure S13). Typically, three independent models were generated for each complex and the quality of the predicted complexes was assessed by examining multiple outputs including the iPTM scores (confidence scores for interfacial residues), predicted alignment error (PAE) plots, and finally a visual inspection of how well the resulting structures aligned with each other in PyMol. Notably, the various complexes invariably displayed highly consistent interfaces across multiple predictions. To generate the final assembled Commander complex, we merged the various predicted structures into a single PDB file, and then models for which we had experimental structures (COMMD1-10 + CCDC22 + CCDC93, VPS29 + VPS35L peptide, and VPS29 + VPS26C + VPS35L) were substituted where appropriate. This complete model was then refined using Phenix to fix various stereochemistry parameters including bond length and Ramachandra outliers to produce a final model. Similarly, we generated analogous complexes using AlphaFold2 implemented in ColabFold to model structures of the *Danio rerio* (zebrafish) and *Salpingoeca rosetta* (single cell choanoflagellate). These were entirely consistent with the predicted human complex.

#### Cell lines

Human cell lines (HeLa, HEK293T, and RPE1) were cultured in humidified incubators at 37°C, 5% CO_2_ in DMEM (Sigma, Catalog number D5796) supplemented with 10% (v/v) fetal bovine serum (Sigma, catalog number F7524) and penicillin/streptomycin (Gibco).

#### Generation of HeLa Retriever KO cell lines

VPS35 knock-out HeLa cells were previously generated.^97^ To generate VPS29 or VPS35L KO HeLa cells, gRNAs targeting genes of interest were designed using the Broad Institute GPP sgRNA Designer and cloned into pSpCas9(BB)-2A-GFP (PX458). HeLa cells were transfected with 2 μg pX458 using FuGene, according to manufacturer’s instructions. Cells were incubated for 24 h before cells were sorted for GFP expression by FACS. Single cells were deposited into 96 well plates containing Iscove’s modified Dulbecco’s medium (Sigma-Aldrich) supplemented with 10% (v/v) FBS (Sigma-Aldrich) and Penicillin/Streptomycin. Single cell clones were expanded and screened for gene KO by lysis and Western blotting.

#### Generation of eHAP COMMD KO cell lines

Human eHap cells were obtained from Horizon Discovery. Cells were cultured in Iscove′s Modified Dulbecco′s Medium (IMDM) supplemented with 10% (v/v) fetal calf serum (FCS; CellSera), and penicillin/streptomycin (Gibco) at 37°C under an atmosphere of 5% CO_2_. Constructs for CRISPR-Cas9 genome editing were designed using the CHOPCHOP website^116^ and oligonucleotides encoding gRNA sequences cloned into the pSpCas9(BB)-2A-GFP (PX458) plasmid (a gift from F. Zhang^117^; Addgene, plasmid 48138) as previously described.^118^ The gRNA sequences and targeting loci are described in STAR Methods. Constructs were transfected using Lipofectamine 3000 (ThermoFisher Scientific) according to manufacturer’s instructions, and single GFP positive cells sorted into 96 well plates. Clonal populations were expanded and screen by a combination of SDS-PAGE and immunoblotting and Sanger sequencing of genomic PCR products cloned into pGEM4Z.^119^ Genomic mutations detected by Sanger sequencing are described in Table S7. For generation of FLAG-tagged cell lines, inserts containing cDNA sequences were commercially synthesized (IDT technologies) to contain a C-terminal FLAG tag and compatible overhangs for Gibson assembly. Inserts were combined with pBABE-puro plasmid (Addgene, 1764) cut with BamHI-HF and HindIII-HF restriction enzymes (NEB) and Gibson assembled using the NEBuilder HiFi DNA Assembly System (NEB) as per manufacturer’s instructions. Retroviral particles were made in HEK293T cells using pUMVC3 and pCMV-VSV-G (Addgene, 8449 and 8454) packaging plasmids as previously described. Viral supernatant was collected at 48 h post-transfection, filtered with 0.45 μm PVDF membrane (Milipore) and combined with 8 μg mL^−1^ polybrene for transduction. Infected cells were selected using 2 μg mL^−1^ puromycin, and transduction verified by SDS-PAGE and immunoblotting.

#### Molecular cloning of VPS35L-GFP

To generate VPS35L-GFP, VPS35L was subcloned into the EGFP-N1 or lentiviral pLVX vector. VPS35L was amplified using Q5 High-Fidelity 2X Master Mix (NEB, M0492) following the manufacturer’s protocol. Following PCR, bands were resolved on agarose gel and purified with GFX PCR DNA and Gel Band purification kit (GE Healthcare, 28-9034-70). Amplified gene or 1 μg of plasmid backbone were then digested using appropriate restriction enzymes (1.5 μL), and the plasmid backbone was additionally treated with 1.5 μL of quick-CIP (NEB, M0525) to prevent self-ligation. Digestion reaction was carried out in 1x CutSmart buffer and nuclease-free water in a final volume of 40 μL at 37°C for 1 h. Digestion products were purified as previous and 50 μg of backbone and 6-times excess of insert were then used for ligation using T4 DNA ligase (Invitrogen, 15224017).

#### Site-directed mutagenesis

Primers for site-directed mutagenesis were designed using Agilent QuikChange Primer design tool. PCR reactions were carried out using QuikChange II Site-Directed Mutagenesis Kit (Agilent, 200523-5) following the manufacturer’s protocol. After PCR, non-mutated template vector was removed from the PCR mixture through digestion for 1 h at 37°C by the enzyme Dpn1. Following digestion by Dpn1, 4 μL of the mixture was transformed into XL10 Gold (Agilent, 200315) chemically competent cells and plated on suitable antibiotic-containing agar plates. Sequencing of purified plasmid DNA established whether the desired mutation had been introduced.

#### Gibson assembly

NEBuilder Assembly Tool was used to design primers for Gibson Assembly reactions. The fragments were amplified using overlapping primers. 0.02 pmol of pLVX_Puro digested with EcoRI and BamHI and −0.04-0.06 pmol PCR-amplified fragments were mixed with Gibson Assembly 2x Master Mix (NEB, E2611) according to manufacturer’s instructions and incubated for 1h at 50°C. 2 μL of the reaction was transformed into NEB 5-alpha Competent *E. coli* (NEB, C2987H) cells.

#### Transfection and transduction of cell lines

PEI (polyethyleneimine) was used to transfect HEK293T cells with constructs for GFP/mCherry traps or to produce lentivirus. An aqueous 10 μg/mL stock of linear 25 kDa PEI (Polysciences, catalog number 23966-2) was used for transfections. For 10 cm or 15 cm, 2.5 mL or 5 mL of Opti-MEM was added to 2 separate sterile tubes respectively. In the first Opti-MEM containing tube, 10 μg or 15 μg DNA was added for 10 cm or 15 cm dishes respectively. To the second tube, 3:1 PEI:DNA ratio was added and the contents vortexed. The Opti-MEM/PEI mixture was then filter sterilised by filtering through a 0.2μm filter. The sterilised PEI/Opti-MEM was then added to the Opti-MEM/DNA mixture and the tube was mixed by vortexing. The mixture was left to incubate at room temperature for 20 min. Following incubation, HEK293T cells were washed in PBS, then PBS was removed and the transfection mixtures were carefully added to the cell dishes. HEK293T cells were incubated with the transfection mixture, under normal growth conditions, for 4 h. The transfection media was removed at the end of the incubation period and replaced with normal growth media. Cells were further incubated for another 24/48 h prior to experimental use. To generate lentivirus, a 15 cm dish of HEK293T cells were transfected with 15 μg of PAX2, 5 μg pMD2.G and 20 μg of lentiviral expression vector using PEI as described above. After the 48 h incubation, the growth media containing the lentivirus was harvested and filtered through a 0.45 μm filter. Cells to be transduced were seeded at 50,000 cells per well of a 6-well plate and left to settle prior to addition of lentivirus.

#### GFP/mCherry nanotraps

Dishes containing cells expressing GFP/mCherry or GFP/mCherry tagged proteins (either transiently or stably) were placed on ice. The cell media was removed and the cells were washed three times with ice-cold PBS (Sigma). Cells were lysed with lysis buffer (20 mM HEPES pH 7.2, 50 mM potassium acetate, 1 mM EDTA, 200 mM D-sorbitol, 0.1% Triton X-100, 1x protease cocktail inhibitor, pH7.5 or 50mM Tris pH7.5 with 0.5% NP-40 in PBS with protease inhibitors). 500 μL or 1 mL of lysis buffer was used per 10 cm or 15 cm dish respectively. Lysis was aided through the use of a cell scraper. The lysates were then cleared by centrifugation at 13,200 rpm for 10 min at 4°C. 15 μL of GFP-trap beads (Chromotek, catalog number gta-20) or mCherry-trap beads (Chromotek, catalog number rta-20) were pre-equilibrated in lysis buffer, through three rounds of washing in lysis buffer, prior to adding cleared cell lysate. 10% of cell lysate was retained for input analysis. Trap beads and lysates were incubated together on a rocker at 4°C for 1 h. Following incubation, Trap beads were pelleted by centrifugation at 2000 rpm, for 30 s at 4°C. Supernatant was then removed, and beads were either washed a further three times in 20 mM HEPES pH 7.2, 50 mM potassium acetate, 1 mM EDTA, 200 mM D-sorbitol, 0.1% Triton X-100, 1x protease cocktail inhibitor, pH7.5 or twice in 50 mM Tris pH7.5 with 0.25% NP-40 in PBS with protease inhibitors and once with 50mM Tris pH7.5 in PBS with protease inhibitors through rounds of re-suspension and pelleting. After the final wash, all lysis buffer was removed from the Trap beads. Beads were then either stored at −20°C or processed for SDS-PAGE analysis.

#### Quantitative western blot analysis

BCA assay kit (ThermoFisher Scientific, USA) was used to determine protein concentration with equal amounts being resolved on 4%–12% NuPAGE precast gels (Invitrogen, USA). Polyvinylidene fluoride membranes (Immobilon-FL; EMD Millipore, USA) were used for transfer with protein detection quantified using the Odyssey infrared scanning system (LI-COR Biosciences, USA) and fluorescently labeled secondary antibodies. We routinely performed western blot quantification where a single blot is simultaneously probed with distinct antibody species targeting proteins of interest followed by visualisation of secondary antibodies conjugated with distinct spectral dyes. All quantified western blots are the mean of at least 3 independent experimental repeats, with statistical analysis performed using Prism 7 (GraphPad Software, USA). All quantitation of western blots is shown in Data S1.

#### Biotinylation of cell surface proteins

Fresh Sulfo-NHS-SS Biotin (ThermoFisher Scientific, no. 21217) was dissolved in 4°C PBS (pH 7.4) at 0.2 mg/mL prior to incubating with prewashed (twice with ice-cold PBS) cells placed on ice to reduce the rate of endocytosis and endocytic pathway flux. Cells were incubated for 30 min at 4°C, followed by incubation in TBS for 10 min to quench the biotinylation reaction. Cells were then lysed in lysis buffer and subjected to Streptavidin bead-based affinity isolation (GE Healthcare, USA).

#### Immunofluorescence staining

Cells were seeded onto sterile 13mm glass coverslips. Once cells were ready to be fixed, growth media was aspirated off and cells were washed three times in PBS prior to fixation in 4% PFA (w/v) (paraformaldehyde, Pierce 16% Formaldehyde (w/v), Methanol-free, catalog number 28906, diluted to 4% (w/v) in PBS). Cells were incubated in 4% PFA for 20 min at room temperature. Coverslips were then washed a further 3 times in PBS. For permeabilization, coverslips were incubated in 0.1% (v/v) Triton X-100 in PBS for 5 min at room temperature. Alternatively, if the cells were going to be stained for LAMP1, cells were permeabilised in 0.1% (w/v) saponin in PBS for 5 min. After permeabilization, coverslips were then washed a further 3 times in PBS. Coverslips were blocked for 15 min in 1% (w/v) BSA in PBS at room temperature. Primary antibodies were diluted in 1% (w/v) BSA in PBS (for Triton X-100 permeabilised cells) or 1% (w/v) BSA, 0.01% (w/v) saponin in PBS. 60μL of diluted antibody solution was pipetted onto a strip of Parafilm as a dot. Coverslips were inverted and placed onto the dots so that the cells were immersed into the antibody solution. Coverslips were incubated with primary antibody for 1 h at room temperature. Coverslips were then washed three times in PBS before placing onto 60 μL dots containing Alexa Fluor-conjugated secondary antibodies and DAPI (if required, 0.5 μg/mL) for 1 h at room temperature, then washed 3 times in PBS and once in water. Coverslips were mounted onto glass microscope slides in Fluoromount-G (Invitrogen, 004958-02).

#### Confocal microscopy

Fixed cells were imaged at room temperature using a Leica SP5, Leica SP5-II or Leica SP8 multi-laser confocal microscope. A 63x NA 1.4 UV oil-immersion lens was used to take all images. Leica LCS or LAS X software was used for the acquisition of images. Colocalisation analysis was performed in Volocity 6.3.1 software (PerkinElmer) with automatic Costes background thresholding.

#### Mass spectrometry of FLAG-COMMDs

Cell pellets (triplicate sub-cultures representing each cell line) were harvested by scraping and washed in PBS (137 mM NaCl, 2.7 mM KCl, 10 mM Na_2_HPO_4_, 1.8 mM KH_2_PO_4,_ pH 7.4). Protein concentration was determined using the Pierce Protein Assay Kit (ThermoFisher Scientific), following which 1 mg of material was solubilized for affinity enrichment mass spectrometry (AEMS) as previously described. Briefly, cell pellets were solubilized in 20mM Tris-Cl pH 7.4, 50mM NaCl, 10% (v/v) glycerol, 0.1mM EDTA, 1% (w/v) digitonin and 125 units of benzonase (Merck) and soluble material loaded onto Pierce Spin Columns (ThermoFisher Scientific) containing anti-FLAG M2 affinity gel (Sigma) pre-equilibrated with 20mM Tris-Cl pH 7.4, 60mM NaCl, 10% v/v glycerol, 0.5mM EDTA, 0.1% w/v digitonin. Following a 2 h incubation at 4°C, columns were washed with the same buffer and enriched protein complexes eluted with the addition of 100 μg mL^−1^ FLAG peptide (Sigma). Eluates were acetone precipitated and precipitates resuspended in 8 M urea in 50 mM ammonium bicarbonate (ABC). Proteins were reduced and alkylated by incubation at 37°C for 30 min with 10 mM tris(2-carboxyethyl)phosphine hydrochloride (TCEP; Bondbreaker, ThermoFisher Scientific) and 50 mM chloroacetamide (Sigma Aldrich), following which samples were diluted to 2 M urea using 50 mM ABC prior to digestion with 1 μg of trypsin (ThermoFisher Scientific) at 37°C overnight. Peptides were acidified to 1% Trifluoroacetic acid (TFA) and desalted using stagetips containing 2x 14G plugs of 3M Empore SDB-XC Extraction Disks (Sigma) as described.^118^ Peptides dried using CentriVap concentrator (Labconco) and samples reconstituted in 0.1% TFA, 2% CAN for analysis by mass spectrometry.

For COMMD1^FLAG^, COMMD6^FLAG^, and COMMD9^FLAG^ and parental eHap1 cell lines, eluates prepared as above were analyzed on an LTQ Orbitrap Elite (Thermo Scientific) in conjunction with an Ultimate 3000 RSLC nano HPLC (Dionex Ultimate 3000) using the liquid chromatography (LC) and mass spectrometry instrument parameters previously described.^120^ The basic LC setup consisted of a trap column (Dionex-C18 trap column 75 μm × 2 cm, 3 μm, particle size, 100 Å pore size; ThermoFisher Scientific) run at 5 μL/min before switching the pre-column in line with the analytical column (Dionex-C18 analytical column 75 μm × 50 cm, 2 μm particle size, 100 Å pore size; ThermoFisher Scientific). The separation of peptides was performed at 300 nL/min using a 95 min non-linear ACN gradient of buffer A [0.1% formic acid, 2% ACN, 5% DMSO] and buffer B [0.1% formic acid in ACN, 5% DMSO]. Mass spectrometry data were collected in Data Dependent Acquisition (DDA) mode using *m*/*z* 300–1650 as MS scan range, rCID for MS/MS of the 20 most intense ions. Lockmass of 401.92272 from DMSO was used. Other instrument parameters were: MS scan at 100,000 resolution, maximum injection time 150 ms, AGC target 1_E_6, CID at 30% energy for a maximum injection time of 150 ms with AGC target of 5000. Dynamic exclusion with of 30 s was applied for repeated precursors. For COMMD2^FLAG^, COMMD4^FLAG^, COMMD5^FLAG^, COMMD7^FLAG^, COMMD8^FLAG^, COMMD10^FLAG^ and parental eHap1 cell lines, eluates were analyzed on an Orbitrap Exploris 480 Thermo Scientific) in conjunction with an Ultimate 3000 RSLC nanoHPLC (Dionex Ultimate 3000) using liquid chromatography and mass spectrometry instrument parameters previously described.^120^ The LC setup was identical to that described above, except that for COMMD2^FLAG^, COMMD4^FLAG^, COMMD5^FLAG^, COMMD7^FLAG^, COMMD8^FLAG^ and parental control, the non-linear ACN gradient used for the separation of peptides was 65 min in length. Mass spectrometry was conducted in data-dependent acquisition mode, whereby full MS1 spectra were acquired in a positive mode at 120000 resolution using a scan range of 300–1600 *m*/*z*. The ‘top speed’ acquisition mode with 3 s cycle time on the most intense precursor ion was used, whereby ions with charge states of 2–6 were selected. MS/MS analyses were performed by 1.2 *m*/*z* isolation with the quadrupole, fragmented by HCD with collision energy of 30%. MS2 resolution was at 15000. AGC target was set to *standard* with auto maximum injection mode. Dynamic exclusion was activated for 20 s.

Affinity enrichment mass spectrometry data were analyzed using the MaxQuant^121^ and Perseus^122^ platforms as previously described for similar data in.^123^ In brief, raw mass spectrometry data from each batch of AEMS experiments were separately analyzed in MaxQuant with the data combined during workup in Perseus. Default MaxQuant search parameters were used with “Label free quantitation” set to “LFQ” and “Match between runs” enabled. Trypsin/P cleavage specificity (cleaves after lysine or arginine, even when proline is present) was used with a maximum of 2 missed cleavages. Oxidation of methionine and N-terminal acetylation were specified as variable modifications. Carbamidomethylation of cysteines was set as a fixed modification. A search tolerance of 4.5 ppm was used for MS1 and 20 ppm for MS2 matching. False discovery rates (FDR) were determined through the target-decoy approach set to 1% for both peptides and proteins. The MaxQuant ProteinGroups.txt output tables were imported into Perseus and LFQ intensities were log2 transformed. Values listed as being “Only identified by site,” “Reverse,” or “Contaminants” were removed from the dataset. Experimental groups were assigned to each set of triplicates and the number of valid values for each row group calculated. For each experiment (containing a control and an enrichment group), single replicates with significant variation as evident through a principal component analysis (PCA) were removed, along with rows having less than 2 valid values in the enrichment group. Missing values in the relevant control group were imputed to values consistent with the limit of detection. A two-sided, two-sample Student’s *t* test was performed between control and each enrichment group, with the resulting data plotted on volcano plot. The threshold of significant enrichment was set to 2-fold (log2 fold change = 1) based on the distribution of unenriched proteins quantified.

#### TMT labeling and high pH RP chromatography

The samples were reduced (10 mM TCEP, 55°C for 1 h), alkylated (18.75 mM iodoacetamide, room temperature for 30 min) and then digested from the beads with trypsin (2.5 μg trypsin; 37°C, overnight). The resulting peptides were then labeled with TMT seven-plex reagents according to the manufacturer’s protocol (ThermoFisher Scientific, Loughborough, LE11 5RG, UK) and the labeled samples pooled and desalted using a SepPak cartridge according to the manufacturer’s instructions (Waters, Milford, Massachusetts, USA). Eluate from the SepPak cartridge was evaporated to dryness and resuspended in buffer A (20 mM ammonium hydroxide, pH 10) prior to fractionation by high pH reversed-phase chromatography using an Ultimate 3000 liquid chromatography system (ThermoFisher Scientific). In brief, the sample was loaded onto an XBridge BEH C18 Column (130 Å, 3.5 μm, 2.1 mm × 150 mm, Waters, UK) in buffer A and peptides eluted with an increasing gradient of buffer B (20 mM Ammonium Hydroxide in acetonitrile, pH 10) from 0 to 95% over 60 min. The resulting fractions (5 in total) were evaporated to dryness and resuspended in 1% formic acid prior to analysis by nano-LC MSMS using an Orbitrap Fusion Tribrid mass spectrometer (Thermo Scientific).

#### Nano-LC mass spectrometry

High pH RP fractions were further fractionated using an Ultimate 3000 nano-LC system in line with an Orbitrap Fusion Tribrid mass spectrometer (Thermo Scientific). In brief, peptides in 1% (v/v) formic acid were injected onto an Acclaim PepMap C18 nano-trap column (Thermo Scientific). After washing with 0.5% (v/v) acetonitrile 0.1% (v/v) formic acid peptides were resolved on a 250 mm × 75 μm Acclaim PepMap C18 reverse phase analytical column (Thermo Scientific) over a 150 min organic gradient, using 7 gradient segments (1–6% solvent B over 1 min, 6–15% B over 58 min, 15–32% B over 58 min, 32–40% B over 5 min, 40–90% B over 1 min, held at 90% B for 6 min and then reduced to 1% B over 1min) with a flow rate of 300 nL min^−1^. Solvent A was 0.1% formic acid and Solvent B was aqueous 80% acetonitrile in 0.1% formic acid. Peptides were ionized by nano-electrospray ionization at 2.0 kV using a stainless-steel emitter with an internal diameter of 30 μm (Thermo Scientific) and a capillary temperature of 275°C. All spectra were acquired using an Orbitrap Fusion Tribrid mass spectrometer controlled by Xcalibur 2.1 software (Thermo Scientific) and operated in data-dependent acquisition mode using an SPS-MS3 workflow. FTMS1 spectra were collected at a resolution of 120,000, with an automatic gain control (AGC) target of 200,000 and a max injection time of 50 ms. Precursors were filtered with an intensity threshold of 5000, according to charge state (to include charge states 2–7) and with monoisotopic peak determination set to peptide. Previously interrogated precursors were excluded using a dynamic window (60s +/−10 ppm). The MS2 precursors were isolated with a quadrupole isolation window of 1.2*m*/*z*. ITMS2 spectra were collected with an AGC target of 10,000, max injection time of 70ms and CID collision energy of 35%. For FTMS3 analysis, the Orbitrap was operated at 50,000 resolution with an AGC target of 50,000 and a max injection time of 105 ms. Precursors were fragmented by high energy collision dissociation (HCD) at a normalised collision energy of 60% to ensure maximal TMT reporter ion yield. Synchronous Precursor Selection (SPS) was enabled to include up to 10 MS2 fragment ions in the FTMS3 scan.

#### Proteomic data analysis

The raw data files were processed and quantified using Proteome Discoverer software v2.1 (Thermo Scientific) and searched against the UniProt Human database (downloaded January 2021; 169297 sequences) using the SEQUEST HT algorithm. Peptide precursor mass tolerance was set at 10 ppm, and MS/MS tolerance was set at 0.6 Da. Search criteria included oxidation of methionine (+15.995Da), acetylation of the protein N-terminus (+42.011Da) and Methionine loss plus acetylation of the protein N-terminus (−89.03Da) as variable modifications and carbamidomethylation of cysteine (+57.021Da) and the addition of the TMT mass tag (+229.163Da) to peptide N-termini and lysine as fixed modifications. Searches were performed with full tryptic digestion and a maximum of 2 missed cleavages were allowed. The reverse database search option was enabled and all data was filtered to satisfy false discovery rate (FDR) of 5%.

#### Phylogenetic analyses

Representative sequences of CCDC22, CCDC93, COMMD1, COMMD2, COMMD3, COMMD4, COMMD5, COMMD6, COMMD7, COMMD8, COMMD9 and COMMD10 were used to construct HMM profilers (HMMER 3.3.2) which were then searched against 30 proteomes from a representative selection of organisms (from RefSeq^124^ and GenBank^125^) with an E-value threshold of 1 × 10^−5^. Duplicate COMMD sequences were removed, and representative query sequences were added (for identification of each different COMMD protein) before sequences were aligned using MAFFT L-INS-i (v7.505),^111^ with separate alignments for CCD22, CCD93 and one alignment for all 10 COMMD proteins. Maximum likelihood trees were then inferred using IQTree (details below) and manually inspected, and outgroups to the COMMD10, CCD22 and CCD93 clades were removed from the unaligned sequence sets before alignment and subsequent tree inference. All maximum likelihood trees were inferred under the best-fitting model according to the Bayesian Information Criterion implemented in ModelFinder (part of IQTree2.1.3^112^), including complex models allowing for across-site compositional heterogeneity (-m MFP -madd LG + C60 + F+G,LG + C50 + F+G,LG + C40 + F+G,LG + C30 + F+G,LG + C20 + F+G,LG + C10 + F+G,LG + F + G,LG + R + F --score-diff ALL). Each tree was inferred with 10,000 ultrafast bootstrap replicates. The best-fitting models were Q.yeast+R5 for the COMMD1-10 tree, LG + C20 + F+G for CCD22, and Q.insect+F+I + G4 for CCD93.

### Quantification and statistical analysis

For data analysis of FLAG-tagged COMMD subunit mass spectrometry experiments, raw files were analyzed using the MaxQuant platform,^126^ version 1.6.10.43 against canonical, reviewed and isoform variants of human protein sequences in FASTA format (Uniprot, January 2019). The default settings: “LFQ” and “Match between runs” were enabled. N-terminal acetylation and methionine oxidation were set as variable modifications while cysteine carbamidomethylation was specified as a fixed modification. Computation of protein enrichment was performed in Perseus (version 1.6.10.43).^122^ Peptides labeled by MaxQuant as ‘only identified by site’, ‘reverse’ or ‘potential contaminant’ were removed and only those proteins quantified based on >1 unique peptide were considered for further analysis. LFQ intensities were log2 transformed and rows having less than 3 valid values in the enrichment group were removed and the missing values in the control group were imputed to values consistent with the limit of detection. The mean log_2_ LFQ intensities for proteins detected in each experimental group, along with p values, were calculated using a two-sided two-tailed t-test. Significance was determined by permutation-based FDR statistics^122^ where the s0 factor was iteratively modified to exclude all identifications enriched in the control experiment, yielding an s0 of 1 at 1% FDR.

For quantitation of Western blots protein detection was performed using the Odyssey infrared scanning system (LI-COR Biosciences, USA) and fluorescently labeled secondary antibodies. We routinely performed western blot quantification where a single blot is simultaneously probed with distinct antibody species targeting proteins of interest followed by visualisation of secondary antibodies conjugated with distinct spectral dyes. All quantified western blots are the mean of at least 3 independent experimental repeats, with statistical analysis performed using Prism 7 (GraphPad Software, USA). Colocalisation analysis of fluorescently labeled proteins was performed in Volocity 6.3.1 software (PerkinElmer) with automatic Costes background thresholding.

## Data Availability

• Coordinates for the COMMD1-10/CCDC22/CCDC93 complex have been deposited at the Protein DataBank (PDB) with accession codes 8F2R (CryoSPARC) and 8F2U (Relion) with respective Electron Microscopy DataBank under accession codes EMD-28825 (CryoSPARC) and EMD-28827 (Relion). Coordinates and structure factors for the crystal structure of the COMMD5-COMMD10-COMMD9-COMMD7 complex have been deposited at the PDB with accession code 8ESD. Coordinates and structure factors for the crystal structure of the VPS29-VPS35L peptide complex have been deposited at the PDB with accession code 8ESE. All datasets are publicly available as of the date of the publication. • Predicted structures of the Commander complex and sub-assemblies using AlphaFold2 have been deposited in the ModelArchive (https://www.modelarchive.org) with accession numbers as outlined in the key resources table. All datasets are publicly available as of the date of the publication. • This paper does not report original code. • Any additional information and all the relevant raw data required to reanalyze the data reported in this paper is available from the lead contact upon request.

## Supplemental information

Table S1. Cryo-EM data collection, refinement, and validation statistics, related to Figures 1 and 4

Table S2. Crystal structure data collection, refinement and validation statistics^a^, related to Figures 2 and 3

Table S3. Primers used in this study and accession numbers for AlphaFold models, related to STAR Methods.

Data S1. Quantification of band intensities from all Western blots and raw images of gels and Western blots included in this study, related to Figures 1, 2, 5, and 6

Methods S1. Additional details of molecular biology, protein purification, cryo-EM data processing, and Alpha-fold modeling used in this study, related to Figures 1–4 and Figure 6
