# Supplementary material for: Structure of the endosomal Commander complex linked to Ritscher-Schinzel syndrome
Source: Cell. 2023 May 11;186(10):2219–2237.e29. doi: 10.1016/j.cell.2023.04.003 (PMC10187114; doi:10.1016/j.cell.2023.04.003)
Supplement: Table S1. Cryo-EM data collection, refinement, and validation statistics, related to Figures 1 and 4 [file mmc1.pdf]

Cell, Volume *186*

Supplemental information

Structure of the endosomal Commander complex linked to Ritscher-Schinzel syndrome

Michael D. Healy, Kerrie E. McNally, Rebeka Butkovič, Molly Chilton, Kohji Kato, Joanna Sacharz, Calum McConville, Edmund R.R. Moody, Shrestha Shaw, Vicente J. Planelles-Herrero, Sathish K.N. Yadav, Jennifer Ross, Ufuk Borucu, Catherine S. Palmer, Kai-En Chen, Tristan I. Croll, Ryan J. Hall, Nikeisha J. Caruana, Rajesh Ghai, Thi H.D. Nguyen, Kate J. Heesom, Shinji Saitoh, Imre Berger, Christiane Schaffitzel, Tom A. Williams, David A. Stroud, Emmanuel Derivery, Brett M. Collins, and Peter J. Cullen

Table S1. Cryo-EM data collection, refinement, and validation statistics. Related to Figures 1 and 4.

	**Retriever**<br>VPS35L-VPS26C-VPS29	**CCC**<br>COMMD1 to COMMD10-CCDC22-CCDC93	
**Data collection and processing**			
Magnification	130,000	75,000	
Voltage (kV)	200	300	
Electron exposure ($e^{-}/Å^2$) Defocus range (µm)	61.7 $e^{-}/Å^2$<br>-2, -1.6, -1.2, -0.8 µm	43.55/42.73 $e^{-}/Å^2$<br>-2, -1.8, -1.6, -1.4, -1.2 µm	
Pixel size (Å)	0.525	1.084	
Symmetry imposed	C1	C1	
Programme used	RELION/cryoSPARC	cryoSPARC	RELION
Initial particle images (no.)	297,077	2,666,858	1,321,587
Final particle images (no.)	119,564	153,104	20,034
Map resolution (Å)	4.3[a]	3.12	3.53
FSC threshold	0.143	0.143	0.143
**Refinement**			
Initial model used		Colabfold	CryoSPARC/Colabfold
Model resolution range (Å)		3.49	3.98
FSC threshold		0.5	0.5
Map sharpening *B* factor ($Å^2$)		83	- 24
Model composition			
Non-hydrogen atoms		29276	36574
Protein residues		1837	2298
R.m.s. deviations			
Bond lengths (Å)		0.005	0.002

Bond angles (°)		1.118	0.561
Validation			
MolProbity score		1.41	1.49
Clashscore		6.01	6.24 (90th percentile)
Poor rotamers (%)		0.12	0
Ramachandran plot			
Favored (%)		97.63	97.18
Outliers (%)		0.17	0
EMDB ID		EMD-28825	EMD-28827
PDB ID		8F2R	8F2U

a. This is significantly overestimated due to preferred particle orientations.
