# Supplementary material for: Structure of the endosomal Commander complex linked to Ritscher-Schinzel syndrome
Source: Cell. 2023 May 11;186(10):2219–2237.e29. doi: 10.1016/j.cell.2023.04.003 (PMC10187114; doi:10.1016/j.cell.2023.04.003)
Supplement: Table S2. Crystal structure data collection, refinement and validation statisticsa, related to Figures 2 and 3 [file mmc2.pdf]

Table S2. Crystal structure data collection, refinement and validation statistics[a]. Related to Figures 2 and 3.

	**COMMD5-COMMD7-COMMD9-COMMD10**	**VPS29-VPS35L peptide**
**Data Collection**		
Space group	C2	P212121
Unit cell dimensions (a,b,c) (Å)	215.19 74.89 63.63	43.72 55.28 82.64
Unit cell angles (α,β,γ) (º)	90.00 96.40 90.00	90.00 90.00 90.00
Wavelength (Å)	0.95372	0.95365
Resolution range (Å)	48.02-3.33 (3.60-3.33)	45.95-1.35 (1.37-1.35)
Total reflections	102614 (20743)	277842 (11606)
Unique reflections	14773 (2987)	44459 (1992)
Multiplicity	6.9 (6.9)	6.2 (5.8)
Completeness (%)	99.0 (97.6)	99.1 (91.6)
Mean $I/\sigma(I)$	8.9 (1.3)	11.3 (1.0)
R-merge	0.108 (1.088)	0.07 (1.47)
R-meas	0.127 (1.278)	0.077 (1.61)
R-pim	0.048 (0.480)	0.031 (0.643)
CC(1/2)	0.999 (0.732)	0.998 (0.481)
**Refinement**		
R-work	0.243 (0.318)	0.219 (0.400)
R-free	0.287 (0.404)	0.246 (0.382)
Number of non-hydrogen atoms	4157	1770
Number macromolecule atoms	4157	1576
Number water atoms	0	194
RMS bonds (Å)	0.002	0.023
RMS angles (º)	0.519	1.95
Ramachandran favored (%)	96.5	97.9
Ramachandran allowed (%)	3.5	2.1
Ramachandran outliers (%)	0	0
Clashscore	2.97	9.8
Overall Molprobity score	1.11	1.7
Average B-factor	141.4	26.7
PDB ID	8ESD	8ESE

a. Statistics for the highest-resolution shell are shown in parentheses.
