# Supplementary material for: Structure of the endosomal Commander complex linked to Ritscher-Schinzel syndrome
Source: Cell. 2023 May 11;186(10):2219–2237.e29. doi: 10.1016/j.cell.2023.04.003 (PMC10187114; doi:10.1016/j.cell.2023.04.003)
Supplement: Table S3. Primers used in this study and accession numbers for AlphaFold models, related to STAR Methods. [file mmc3.pdf]

Table S3. Primers used in this study and accession numbers for AlphaFold models

Primer name	Sequence (5'->3')
VPS35L (transfer to pEGFP-N1) F	GACTGTCGACTGCCACCATGGCCGTCTTTCCTTGG
VPS35L (transfer to pEGFP-N1) R	CTATGGATCCATGTACAAGAAAGCTGGGT
VPS35L_deltaA2-F17_F	GACTGTCGACTGCCACCATGGCATCATGCCGACTGGAG
VPS35L (transfer to pLVX) F	GACTCCCGGGTAATGGCCGTCTTTCCTTGG
VPS35L (transfer to pLVX) R	CTATTCTAGATTACTTGTACAGCTCGTC
VPS35L_V158D_F	CTCCAGCCGGGTCCGATCCTTCTCTGACATTG
VPS35L_V158D_R	CAATGTCAGAGAAGGATCGGACCCGGCTGGAG
VPS35L_L162D_F	GTCATCCAGCTCCTCATCCCGGGTCCGCACCTT
VPS35L_L162D_R	AAGGTGCGGACCCGGGATGAGGAGCTGGATGAC
VPS35L_F282D_F	TGATGGAGGCAATCTTGTCAAACCAATTTAGGCATGTTTCCTTGG
VPS35L_F282D_R	CCAAGGAAACATGCCTAAATTGGTTTGACAAGATTGCCTCCATCA
VPS35L_A285D_F	GGAATGAGTTCCCTGATGGAGTCAATCTTGAAAAACCAATTTA
VPS35L_A285D_R	TAAATTGGTTTTTCAAGATTGACTCCATCAGGGAACTCATTCC
VPS35L_R293E_F	CCTCCATCAGGGAACTCATTCCAGAGTTTTACGTGGAGGCATCCA
VPS35L_R293E_R	AGGATGGATGCCTCCACGTAAAACTCTGGAATGAGTTCCCTGATG
VPS35L_H676E_F	ACCGGTTCACACTCTCAATCAACTGCACAAGAACAGGCTCC
VPS35L_H676E_R	GGAGCCTGTTCTTGTGCAGTTGATTGAGAGTGTGAACCGGT
VPS35L_H826E_F	CTACACCTGCGTCCTGGAGCTCCTCTCCGCCA
VPS35L_H826E_R	CATGGCGGAGAGGAGCTCCAGGACGCAGGTG
VPS35L_S781D_F	GATCCGGAACTATTAATAAAGTATCAAAGAAATTGCAGAGGAATTCCAGAAGG
VPS35L_S781D_R	CCTTCTGGAATTCCTCTGCAATTTCTTTGATACTTTATTAATAGTTCCGGATC
VPS35L_D574R _F	CACACTCTCTTTTTGGAACATGCGCAGAAACGGCAGAAATTTTTCC
VPS35L_D574R _R	GGAAAAATTTCTGCCGTTTCTGCGCATGTTCCAAAAAGAGAGTGTG
VPS29_L67D_F	CACAACTTTCTGTTCTGGATAATTATCATTCTCATCGAAGTCTCCTCTCAC
VPS29_L67D_R	GTGAGAGGAGACTTCGATGAGAATGATAATTATCCAGAACAGAAAGTTGTG
VPS29_I91D_F	ATCTCCCCATGGATCAACTTGATGTCCATGGATCAGACCA
VPS29_I91D_R	TGGTCTGATCCATGGACATCAAGTTGATCCATGGGGAGAT
VPS29_W93A_F	TGGCCATATCTCCCGCTGGAATAACTTGATGTCCATGGAT
VPS29_W93A_R	ATCCATGGACATCAAGTTATTCCAGCGGGAGATATGGCCA
VPS29_L142D_F	CAAATGATGGAATAATGTTTGTTTCATCGGCATTATATGCCCCAGTGGCAGA
VPS29_L142D_R	TCTGCCACTGGGGCATATAATGCCGATGAAACAAACATTATTCCATCATTTG
VPS29_L152E_F	CTGTAGAAGCCTGGATATCCATCTCCACAAATGATGGAATAATGTTTGT
VPS29_L152E_R	ACAAACATTATTCCATCATTTGTGGAGATGGATATCCAGGCTTCTACAG
VPS29_V174D_F	AAGGTTTTTTGTATTCGATTCGTTCATCTTTCACATCATCTCCAATTAGCTG
VPS29_V174D_R	CAGCTAATTGGAGATGATGTGAAAGATGAACGAATCGAATACAAAAAACCTT
VPS35L_I761G_F	CGCATCTTCCCATCACCATTAATCATCTTTGGAACTTCCGGAACAA
VPS35L_I761G_R	TTGTTCCGGAAGTTCCAAAGATGATTAATGGTGATGGGAAGATGCG
VPS35L_D762G_F	ATGGCCGCATCTTCCCACCAATATTAATCATCTTTGGAACT
VPS35L_D762G_R	AGTTCCAAAGATGATTAATATTGGTGGGAAGATGCGGCCAT
VPS35L_K764G_F	CGATTCCGATGGCCGCATCCCCCCATCAATATTAATCATCTTT
VPS35L_K764G_R	AAAGATGATTAATATTGATGGGGGGATGCGGCCATCGGAATCG
VPS35L_R766G_F	GAACGATTCCGATGGCCCCATCTTCCCATCAATAT
VPS35L_R766G_R	ATATTGATGGGAAGATGGGGCCATCGGAATCGTTC
VPS35L.Nt.miniB eta.GA.R	CCAGAAGGAACGATTCCGA ACCCCCCCCTGGAACTTC CGGAACAAGGC
VPS35L.Ct.miniB eta.GA.F	TTCGGAATCGTTCCTTCTG GAAT
VPS35L_GA	GATCTCGAGCTCAAGCTTCGCCCGGGATGGCCGTCTTT
VPS35L-STOP-GA_R	AGAATTATCTAGAGTCGCGGTTACTTGTACAGCTCGTCCATGC
VPS35L_F4R_F	CTGGAGTGCCAAGGACGGACGGCCATGGTGGC
VPS35L_F4R_R	GCCACCATGGCCGTCCGTCCTTGGCACTCCAG
VPS35L_S8A_F	GTAGTTCCTATTCCTGGCGTGCCAAGGAAAGACGG
VPS35L_S8A_R	CCGTCTTTCCTTGGCACGCCAGGAATAGGAACTAC
VPS35L_R9D_F	TTCAGCTTTGTAGTTCCTATTATCGGAGTGCCAAGGAAAGACGGC
VPS35L_R9D_R	GCCGTCTTTCCTTGGCACTCCGATAATAGGAACTACAAAGCTGAA
double_R11D_F	GATGCAAATTCAGCTTTGTAGTTATCATTATCGGAGTGCCAAGGAAAGACGG
double_R11D_R	CCGTCTTTCCTTGGCACTCCGATAATGATAACTACAAAGCTGAATTTGCATC
VPS35L_L825K_F	GGCGGAGAGGAGATGCTTGACGCAGGTGTAGATG
VPS35L_L825K_R	CATCTACACCTGCGTCAAGCATCTCCTCTCCGCC
VPS35L_S829A_F	TGGCTCATGGCGGCGAGGAGATGCAGG
VPS35L_S829A_R	CCTGCATCTCCTCGCCGCCATGAGCCA
VPS35L_L27D_F	GGTGATAGTCCCCAAACTCATCTGGTACAGCCTCCAGTCGG
VPS35L_L27D_R	CCGACTGGAGGCTGTACCAGATGAGTTTGGGGACTATCACC
VPS35L_L35D_F	CTCTGTGACAGTTATGGGTTTATCAGGGTGATAGTCCCCAAACTC
VPS35L_L35D_R	GAGTTTGGGGACTATCACCCTGATAAACCCATAACTGTCACAGAG
VPS35L_R661A_F	TCCAGATTGCAAAACATCGACGCGGACTCAACATAAAAACTCAG
VPS35L_R661A_R	CTGAGTTTTTATGTTGAGTCCGCGTCGATGTTTTGCAATCTGGA
VPS35L_I710D_F	CAGGGAGGGGATGGTGTCGAAGCAGTAGGCAACA
VPS35L_I710D_R	TGTTGCCTACTGCTTCGACACCATCCCCTCCCTG
CCDC22_V501D_F	GCTTCCGGATGTTGCCATCGATCTCCAGGATGCG
CCDC22_V501D_R	CGCATCCTGGAGATCGATGGCAACATCCGGAAGC
CCDC93_R500A_F	CTGGCGGTAGAGTTCAATAAATGCCTTCTGATACTGTATTAGCTCG
CCDC93_R500A_R	CGAGCTAATACAGTATCAGAAGGCATTTATTGAACTCTACCGCCAG
CCDC93_E503R_F	AATCTGGCGGTAGAGTCTAATAAATCTCTTCTGATACTGTATTAGCTCG
CCDC93_E503R_R	CGAGCTAATACAGTATCAGAAGAGATTTATTAGACTCTACCGCCAGATT
CCDC93_R603E_F	CTTTCACAGTCTTAAAGTATAGCTCCTGCTTTTCTAACAGCTCCAAGT
CCDC93_R603E_R	ACTTGGAGCTGTTAGAAAAGCAGGAGCTATACTTTAAGACTGTGAAAG
COMMD1_EcoR1_F	CTTGGAATTCGATGGCGGCGGGCGAG
COMMD1_KpnI_R	GTTGGGTACTCAGTTAGGCTGGCTGATCA
COMMD2_EcoR1_F	CTTGGAATTCGATGCTGCTGGAATTGTCCG
COMMD2_Kpn1_R	GTTGGGTACCTACTTGATGTTGCGAACAA
COMMD6_EcoR1_F	CTTGGAATTCGATGGAGGCGTCCAGCGA
COMMD6_Kpn1_R	GTTGGGTACTCACACCGTTTCAATAACTG
COMMD7_EcoR1_F	CTTGGAATTCGATGGGCCGCCTGCACTG
COMMD7_Kpn1_R	GTTGGGTACTCAGCAGAAACACTCCATGC
COMMD8_EcoR1_F	CTTGGAATTCGATGGAGCCGGAAGAGGG
COMMD8_Kpn1_R	GTTGGGTACTCAGTTAGGCTGGCTGATCA
COMMD9_EcoR1_F	CTTGGAATTCGATGCTGCTGGAATTGTCCG
COMMD9_Kpn1_R	GTTGGGTACCTACTTGATGTTGCGAACAA
COMMD10_EcoR1_F	CTTGGAATTCGATGGAGGCGTCCAGCGA
COMMD10_Kpn1_R	GTTGGGTACTCACACCGTTTCAATAACTG
Kozak_GFP_F	CTTGGAATTCGATGGGCCGCCTGCACTG
Kozak_GFP_R	GTTGGGTACTCAGCAGAAACACTCCATGC
COMMD1_WT_F	CTTGGAATTCGATGGAGCCGGAAGAGGG
COMMD1_WT_R	GTTGGGTACTTATTTCAACTGCAGGACCA
COMMD1_N-terminal His_Tag_F	CATGGGATCCATGCATCACCATCACCATCACGGTAGCGGCAGCGGTAGCGAAAACCTGTATTTTCAGGGCATGGCGGC GGGCGAGCTTGA
COMMD1_N-terminal His_Tag_R	GTTGGGTACTCATGTAAGGGAATCCAGCT
CCDC93_C-terminal_Triple_Strep_Tag_F	CCCGGGGGGGATCTAGCTAGCGCTACCGGTCGC
CCDC93_C-terminal_Triple_Strep_Tag_R	CATGTCTAGATTACTATTTTTCGAACTGCGGGTGGCTCCATTTTTCGAACTGCGGGTGGCTCCATTTTTCGAACTGCGGG TGGCTCCAGCTACCGCTGCCGCTACCGCCCTGAAAATACAGGTTTTCGGAGGCCTTCGCTTTCACCTTGGAC
pB_pIDC_CasI_F	CTGTACAAGGGTGGAAGCGGTGGAATGGCGGCGGGCGAGCTT
pB_pIDC_CasI_R	CCAGATGGCCATCCGGTTAGGCTGGCTGATCAGTGTGCTGATAC
pB_pIDC_CasII_F	CTGTACAAGGGTGGAAGCGGTGGAATGGCGGCGGGCGAACTG
pB_pIDC_CasII_R	CCAGATGGCCATCCGTTAATTCGGTTGGCTAATCAGGGTGCTAATGC
pB_pIDC_CasIII_F	CATGGGATCCATGCATCACCATCACCATCACGGTAGCGGCAGCGGTAGCGAAAACCTGTATTTTCAGGGCATGGCGGC GGGCGAGCTTGA
pB_pIDC_CasIII_R	CATGAAGCTTTTACTAGTTAGGCTGGCTGATCAGTGTGC
pB_pIDC_CasIV_F	CATGGGATCCATGGGGTTGCCCAGGGGGCCGGA
pB_pIDC_CasIV_R	CATGTCTAGATTACTATTTTTCGAACTGCGGGTGGCTCCATTTTTCGAACTGCGGGTGGCTCCATTTTTCGAACTGCGGG TGGCTCCAGCTACCGCTGCCGCTACCGCCCTGAAAATACAGGTTTTCGGAGGCCTTCGCTTTCACCTTGGAC
pB_pIDC_CasV_F	AACGCTCTATGGTCTAAAGATTTAAATCGACCTACTCCGGAATATTAATAGATC
pB_pIDC_Casw_R	AAACGTGCAATAGTATCCAGTTTATTTAAATGGTTATGATAGTTATTGCTCAGCG
pIDS_CasI_F	AACGCTCTATGGTCTAAAGATTTAAATGGCACCTAGGTATCGATACTAGTATAC
pIDS_CasI_R	AAACGTGCAATAGTATCCAGTTTATTTAAATGGTTATGATAGTTATTGCTCAGCG
pIDS_CasII_F	AAACTGGATACTATTGCACGTTTAAATGGCACCTAGGTATCGATACTAGTATAC
pIDS_CasII_R	AAACATCAGGCATCATTAGGTTTATTTAAATGGTTATGATAGTTATTGCTCAGCG
pIDS_CasIII_F	AAACCTAATGATGCCTGATGTTTAAATGGCACCTAGGTATCGATACTAGTATAC
pIDS_CasIII_R	AAACTAAGCTATGTGAACCGTTTATTTAAATGGTTATGATAGTTATTGCTCAGCG
pIDS_CasIV_F	AAACGGTTCACATAGCTTAGTTTAAATGGCACCTAGGTATCGATACTAGTATAC
pIDS_CasIV_R	AAACCAAGTCAATGTCAGTGTTTATTTAAATGGTTATGATAGTTATTGCTCAGCG
pIDS_CasV_F	AAACACTGACATTGACTTGGTTTAAATGGCACCTAGGTATCGATACTAGTATAC
pIDS_Casw_R	AACCCCGATTGAGATATAGATTTATTTAAATGGTTATGATAGTTATTGCTCAGCG
pB_pKL_CasI_R	AAACTGGATACTATTGCACGTTTAAATGGCACCTAGGTATCGATACTAGTATAC
pB_pKL_CasII_R	AAACATCAGGCATCATTAGGTTTATTTAAATGTACCCGTAGTGGCTATGGCAGGG
pB_pKL_CasIII_R	AAACCTAATGATGCCTGATGTTTAAATGGCACCTAGGTATCGATACTAGTATAC
pB_pKL_CasIV_R	AAACTAAGCTATGTGAACCGTTTATTTAAATGTACCCGTAGTGGCTATGGCAGGG
pB_pKL_CasV_R	AAACGGTTCACATAGCTTAGTTTAAATGGCACCTAGGTATCGATACTAGTATAC

gRNA sequences for HeLa KO

Gene	Exon	gRNA sequence
VPS29	2	GGCAAACTGTTGCACCGGTG
	3	GGACATCAAGTTATTCCATG
VPS35L	1	GCAGACGCCATGCTTGTGAG

gRNA sequences for eHAP KO

Gene	UniprotID	gRNA (PAM)	KO verified by SDS-PAGE	Sequencing primers	Mutation	Protein impact
COMMD1	Q8N668	CGCTGGAATAG	Yes	CGGGGATCCTCTTGACTGCTCAAACCAAAAA / GACAAGCTTTATTGGCCCATTCTGTTCTTCT	c.[356_363del]	p.L119Pfs*7
COMMD2	Q86X83	GCGGCGCCTTC	Yes	CGGGGATCCTTCTGACTTTCATTGCAGTGGT / GACAAGCTTCTTGCAATTTACAGTTGACCCA	c.[127_129insAA]	p.I43Kfs*43
COMMD3	Q9UBI1	GCCGAGCGGCT	Yes	CGGGGATCCCTTCGACTCCAACGCCTTC / GACAAGCTTAACAATGCTCAGAGTCTGCTCC	c.[138_139insTA]	p.D47Ter
COMMD4	Q9H0A8	AGCGGGATACT	Yes	NA	NA	NA
COMMD5	Q9GZQ3	AGCAATGGCCC	Yes	NA	NA	NA
COMMD6	Q7Z4G1	CGTTGCAGTGATGCTAAAAGTGG	No antibody available	CGGGGATCCAAGGAAACCAATACTGCCACAT / GACAAGCTTAAACCTGAAACTGTGGAATCGT	c.[143_144ins(200)]	p.K48Nfs*91
COMMD7	Q86VX2	ACCCTTGCTCG	Yes	NA	NA	NA
COMMD8	Q9NX08	TACCTTATTCGC	Yes	CGGGGATCCTTTAAACAGCTTGCACTTTCCA / GACAAGCTTAGGTCAAGAGTTTGAGACCAGC	c.[514_531+102del	p.L172Ter
COMMD9	Q9P000	CACGCCTGGTCGATCTGGACTGG	No antibody available	CGGGGATCCGATACGCTGCCACTGTGAATTA / GACAAGCTTAAGGAGGATAAAGGGGATGGTA	c.[368_376delins(124);G379A]	p.V124Pfs*19
COMMD 10	Q9Y6G5	AGAATCCGAGT	Yes	NA	NA	NA

AlphaFold2 predicted complexes

https://www.modelarchive.org

Model number	Species	Proteins	ModelArchive ID
1	*Homo sapiens*	COMMD1-10 + CCDC22 (1-223) + CCDC93 (1-300)	ma-iplv4
2	*Homo sapiens*	CCDC22 + CCDC93	ma-9nv72
3	*Homo sapiens*	VPS35L+ VPS26C + VPS29	ma-3cag5
4	*Homo sapiens*	VPS35L + VPS26C + VPS29 + CCDC22 (1-115) + CCDC22 (386-627) + CCDC93 (378-631)	ma-4097m
5	*Homo sapiens*	Combined models 1 + 2 + 3	ma-ri7tb
6	*Danio rerio*	COMMD1-10	ma-99f82
7	*Salpingoeca rosetta*	COMMD1-10	ma-xfc84
