# Supplementary material for: Structure of the endosomal Commander complex linked to Ritscher-Schinzel syndrome
Source: Cell. 2023 May 11;186(10):2219–2237.e29. doi: 10.1016/j.cell.2023.04.003 (PMC10187114; doi:10.1016/j.cell.2023.04.003)

Data S1. Quantified band intensities from all western blots in this study. Page 1/1

Band intensities were quantified using Odyssey software and normalized to GFP expression. 2-way ANOVA with Dunnett's multiple comparisons test. Error bars represent the mean, s.d.

*P < 0.05, **P < 0.01, ***P < 0.001, ****P < 0.0001.

Figure 1 blot quantification

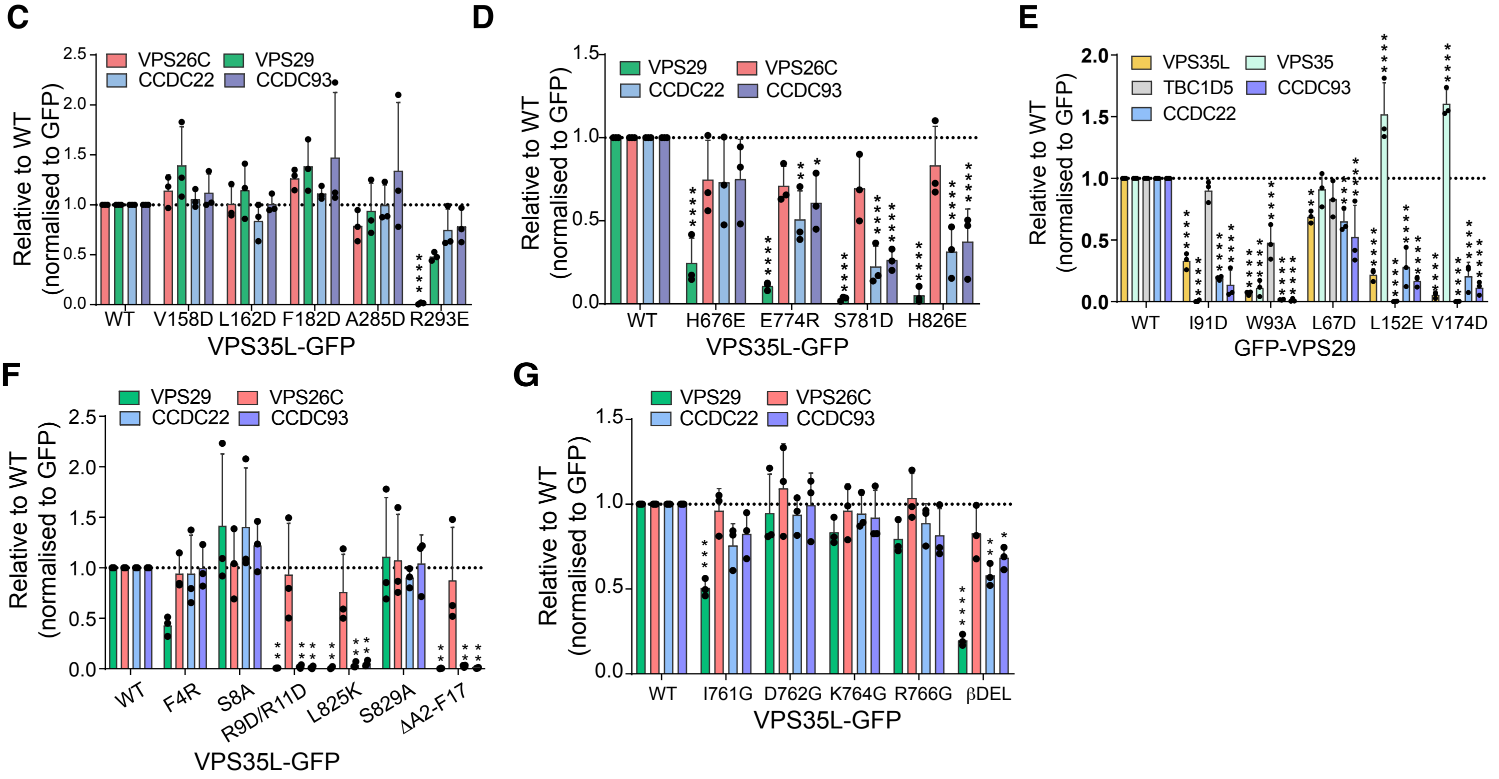

Figure 2 blot quantification

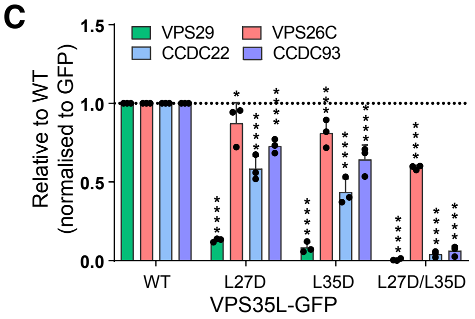

Figure 5 blot quantification

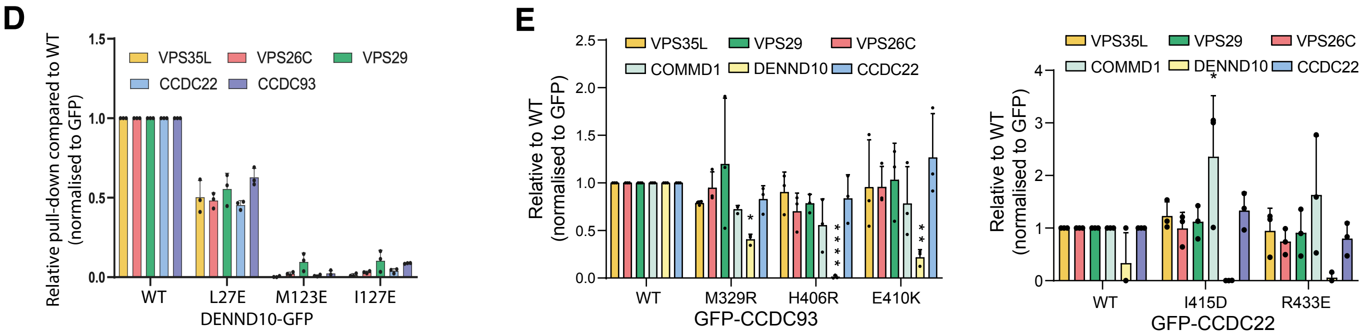

Figure 6 blot quantification

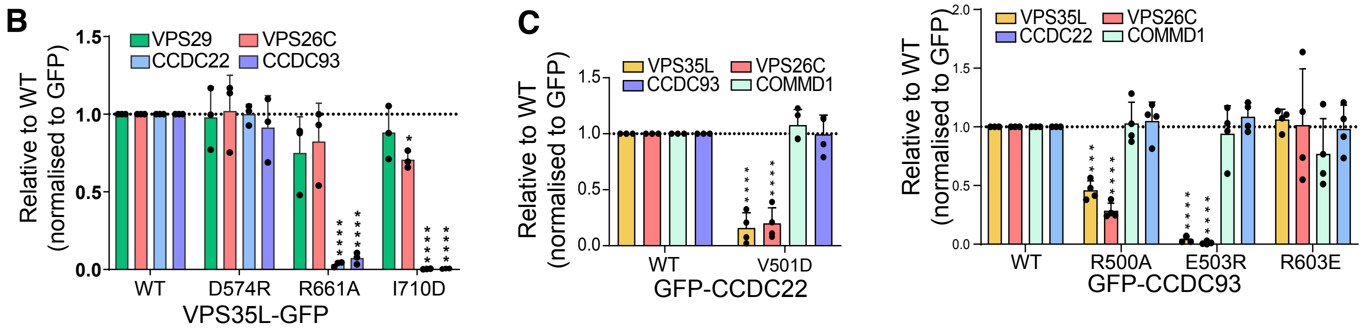

Raw images of gels and western blots included in this study. Page 1/3

Raw data for Fig1C:

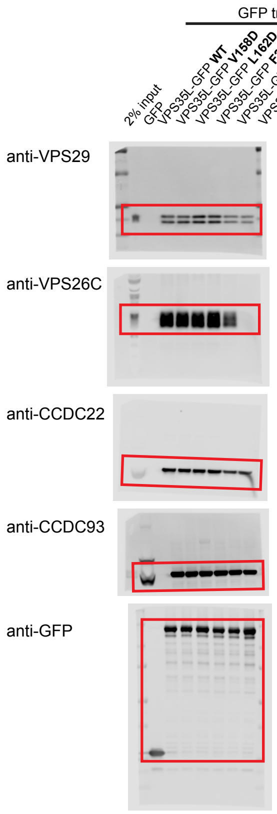

Raw data for Fig1D:

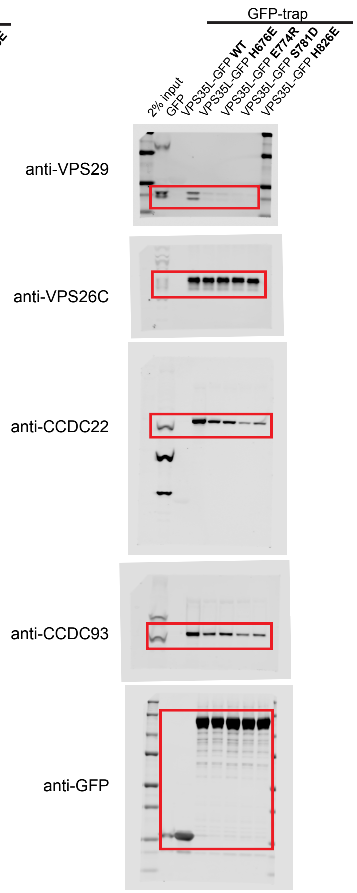

Raw data for Fig1E:

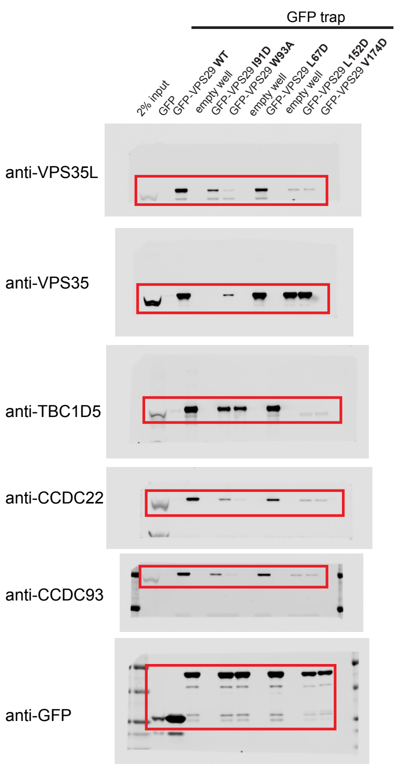

Raw data for Fig1F:

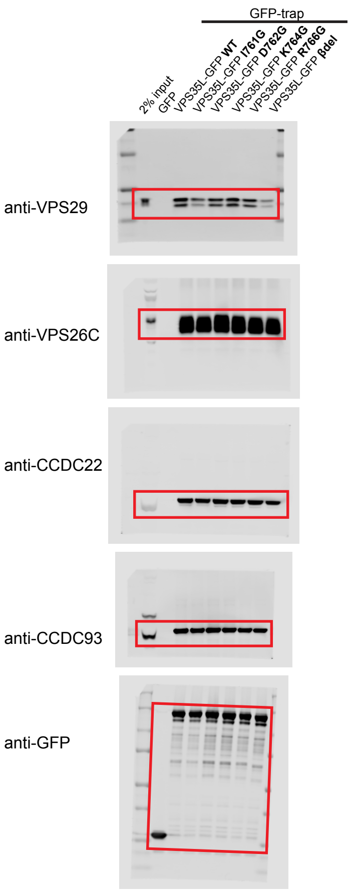

Raw data for Fig1G:

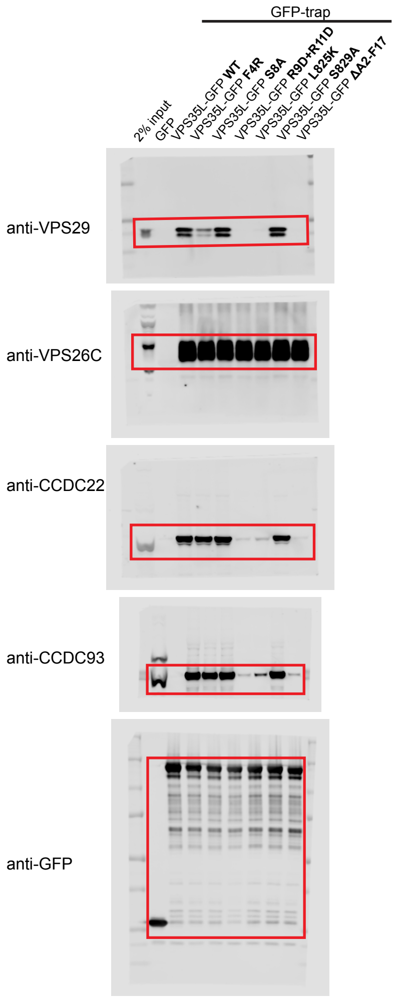

Raw data for Fig2C:

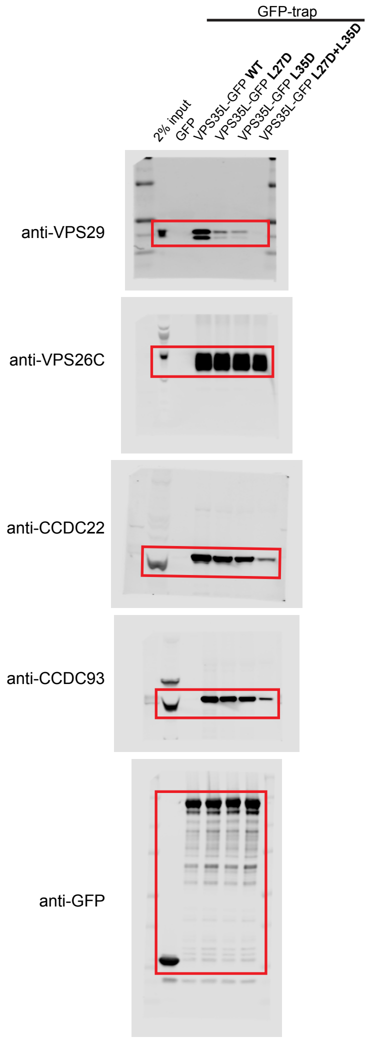

Raw data for Fig2E:

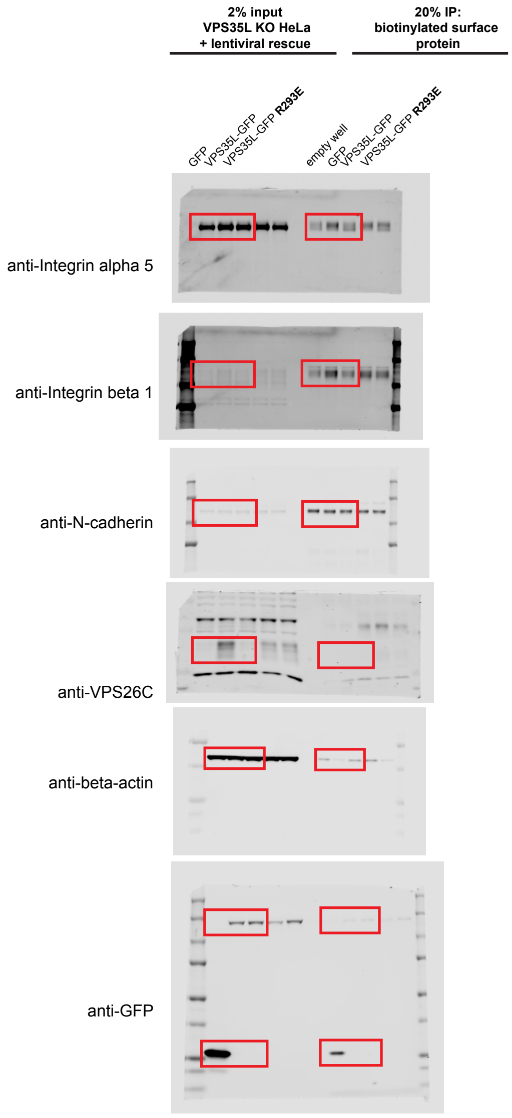

Raw images of gels and western blots included in this study. Page 2/3

Raw data for Fig5B:

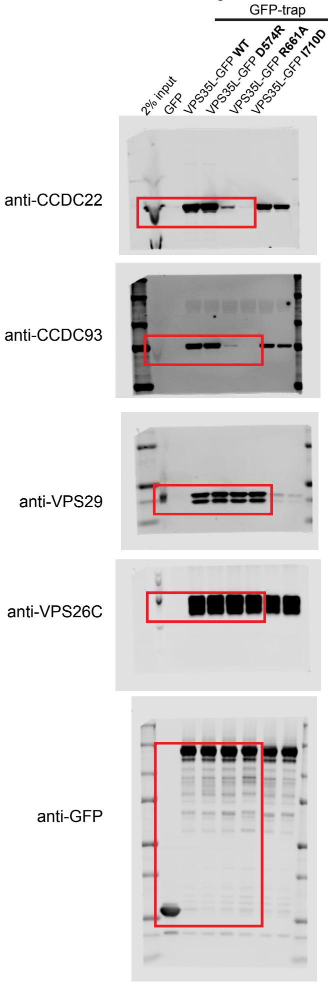

Raw data for Fig5C:

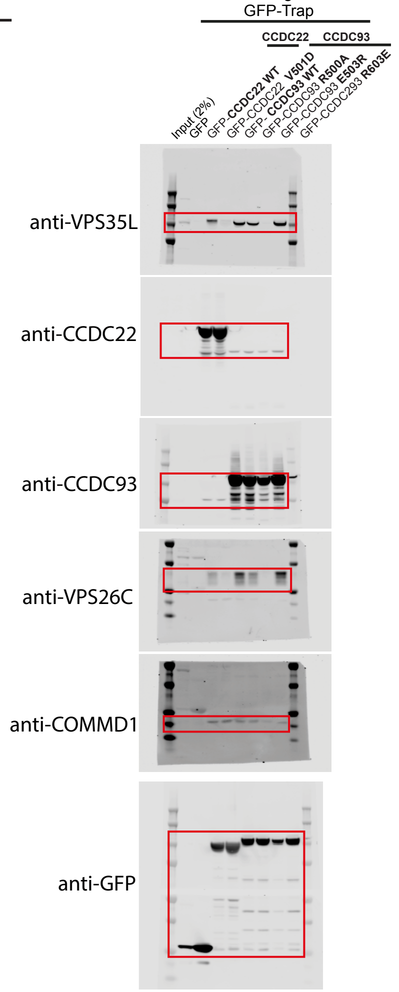

Raw data for FigS2C:

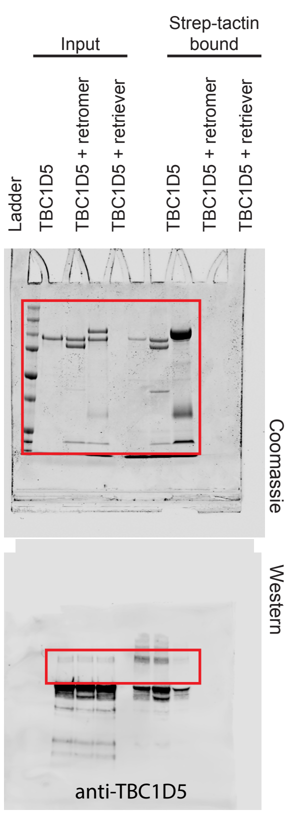

Raw data for FigS2D:

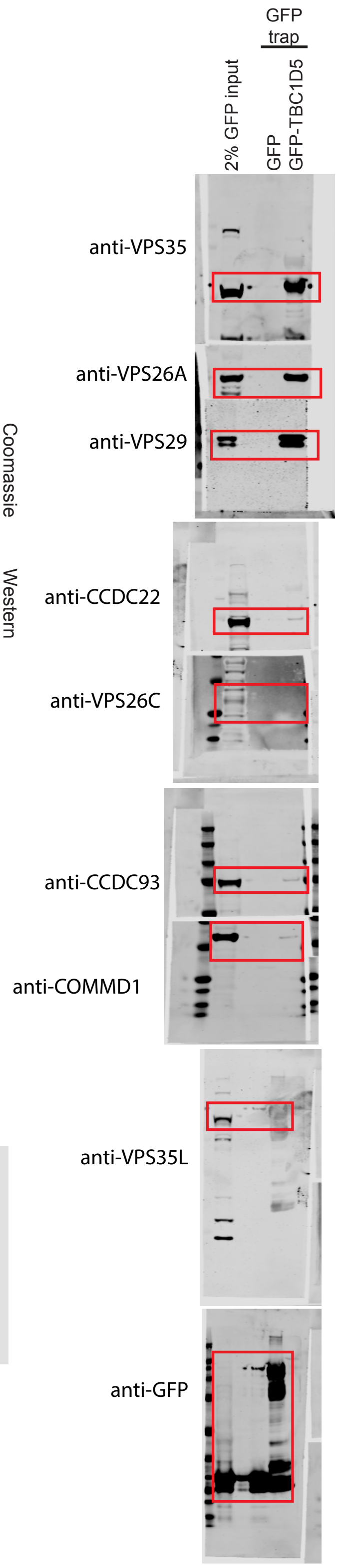

Raw data for FigS2E:

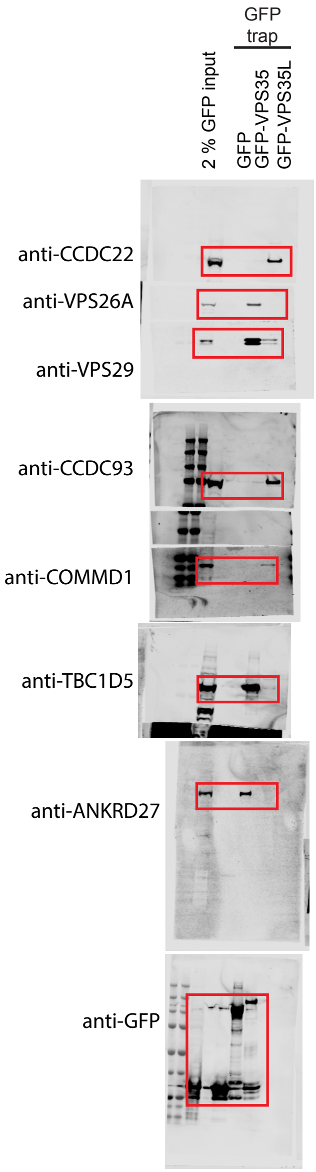

Raw data for FigS1B:

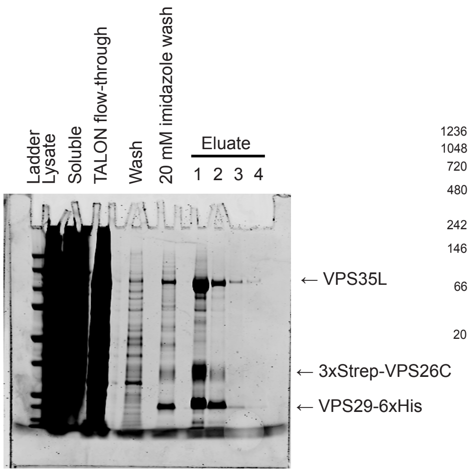

Coomassie gel

Raw data for FigS1D:

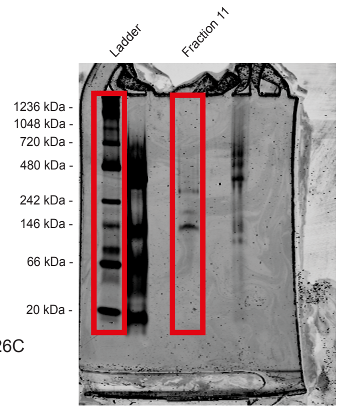

Raw data for FigS1C:

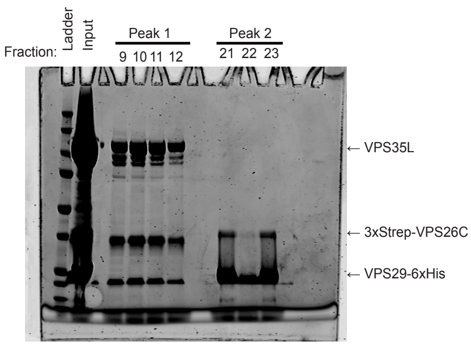

Coomassie gel

Raw images of gels and western blots included in this study. Page 3/3

Raw data for FigS2H:

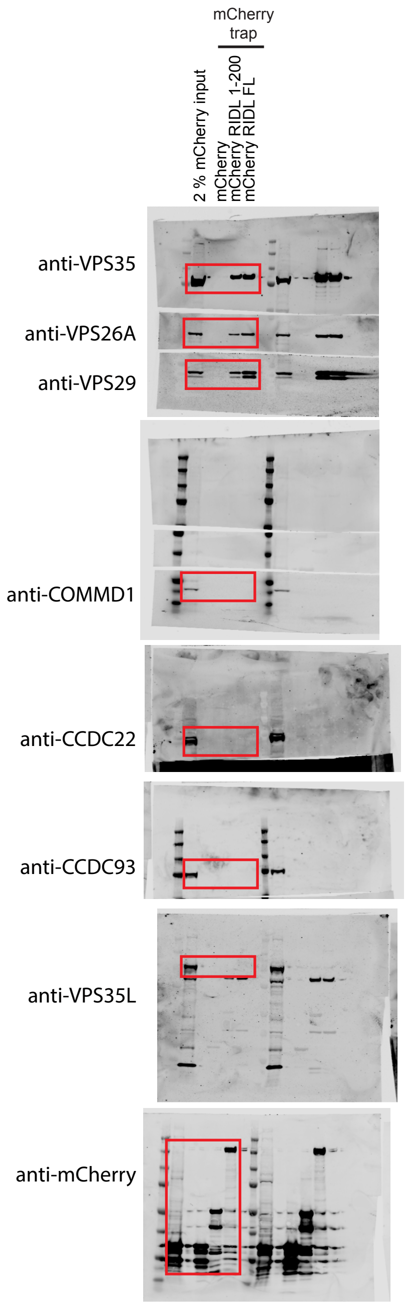

Raw data for FigS4B:

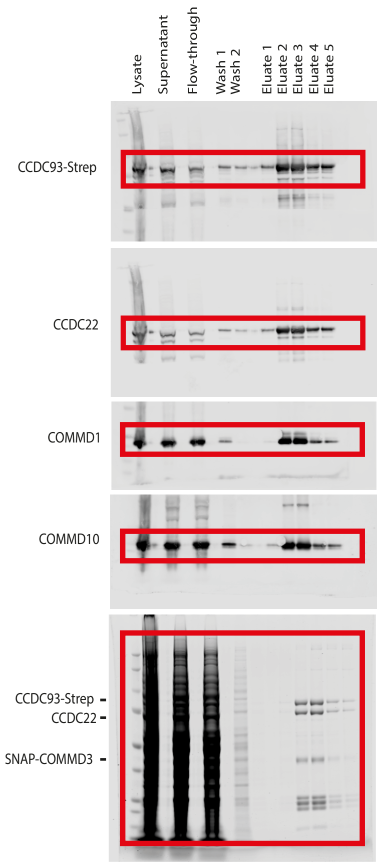

Raw data for Fig4B:

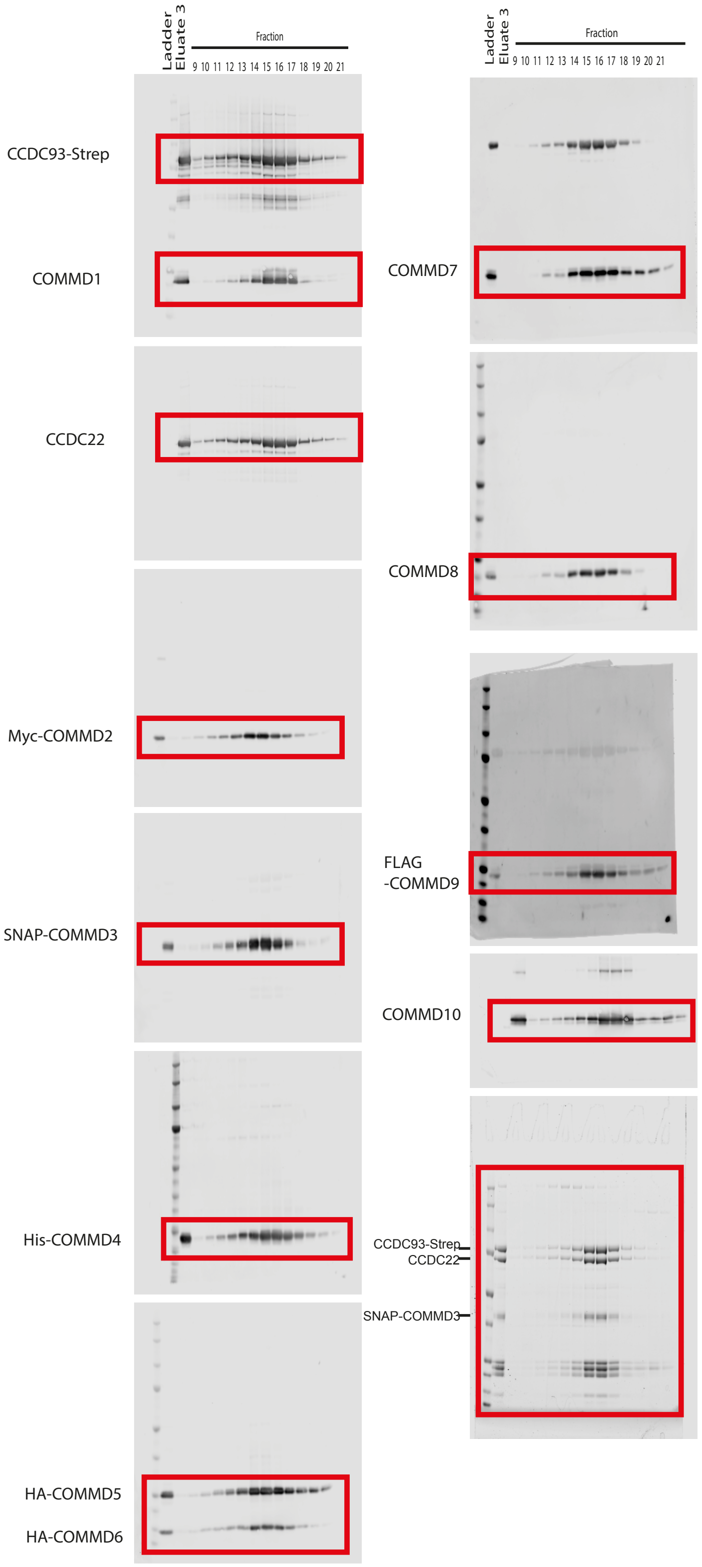

Raw data for FigS3C:

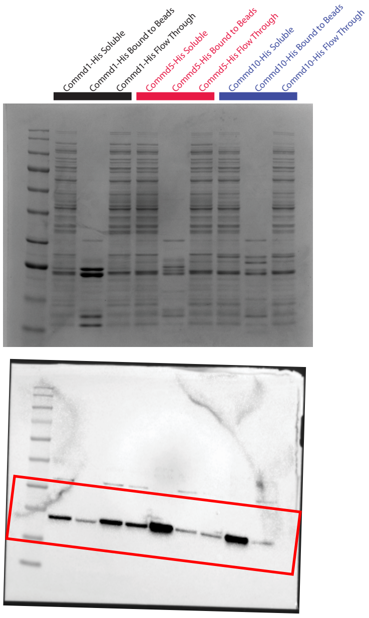

Raw data for FigS4D:

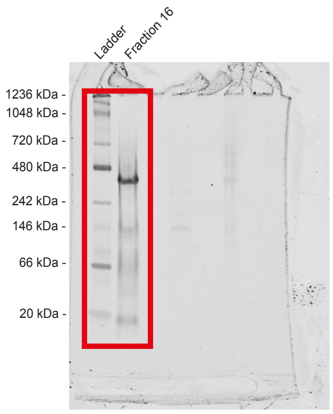

Raw data for FigS11C:

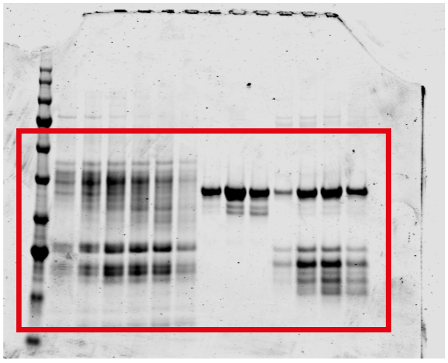

Supplement: Data S1. Quantification of band intensities from all Western blots and raw images of gels and Western blots included in this study, related to Figures 1, 2, 5, and 6 [file mmc4.pdf]
