# Supplementary material for: Structure of the endosomal Commander complex linked to Ritscher-Schinzel syndrome
Source: Cell. 2023 May 11;186(10):2219–2237.e29. doi: 10.1016/j.cell.2023.04.003 (PMC10187114; doi:10.1016/j.cell.2023.04.003)
Supplement: Methods S1. Additional details of molecular biology, protein purification, cryo-EM data processing, and Alpha-fold modeling used in this study, related to Figures 1–4 and Figure 6 [file mmc5.pdf]

Methods S1.

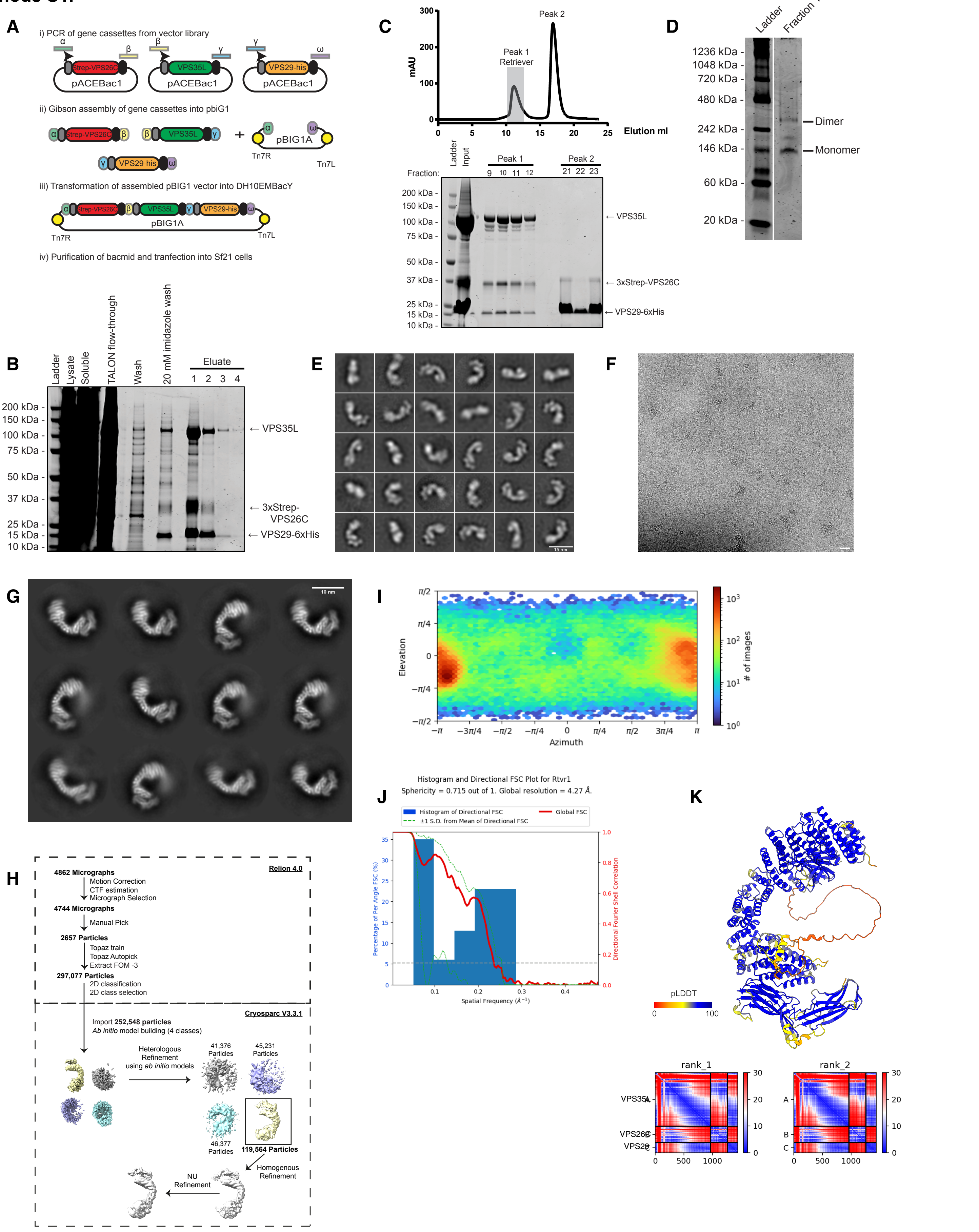

Expression, purification, cryoEM analysis and Alphafold modelling of the human Retriever complex.
(A) Schematic for the biGBac cloning approach used to co-express Retriever in insect cells. (B) Representative Coomassie stained SDS-PAGE gel of the isolation of recombinant Retriever from Sf21 insect through affinity purification of VPS29-6xhis. Eluate fractions were combined and subjected to gel filtration. (C) Gel filtration profile of his-purified Retriever. Retriever was gel filtrated using a Superdex200 column. Fractions corresponding to A280 peaks were analysed by running SDS-PAGE gel and stained with Coomassie. Full Retriever complex corresponds to Peak 1. (D) Native PAGE of purified Retriever. (E) Negative stain electron microscopy analysis of Retriever revealed the elongated 'footprint'-like morphology. Representative 2D classification classes of negatively stained Retriever. Scale bar represents 15 nm. (F) Representative motion-corrected micrograph. (G) Representative single particle cryoEM 2D class averages of Retriever. Note that only a 'front' view of the complex is visible in 2D classes. Scale bar represents 10 nm. (H) Data processing used to obtain a low resolution cryo-EM reconstruction of Retriever. (I) Angular projections of Retriever particles used for the final 3D reconstruction, indicating preferential orientation of the particle. Heat map calculated in CryoSPARC displaying the number of particles per viewing orientation. (J) Directional FSC plots and sphericity values for the Retriever reconstruction. These data were generated using a 3D-FSC server (https://3dfsc.salk.edu/). (K) AlphaFold2 prediction of Retriever with associated PAE plots for the top 2 ranked models.

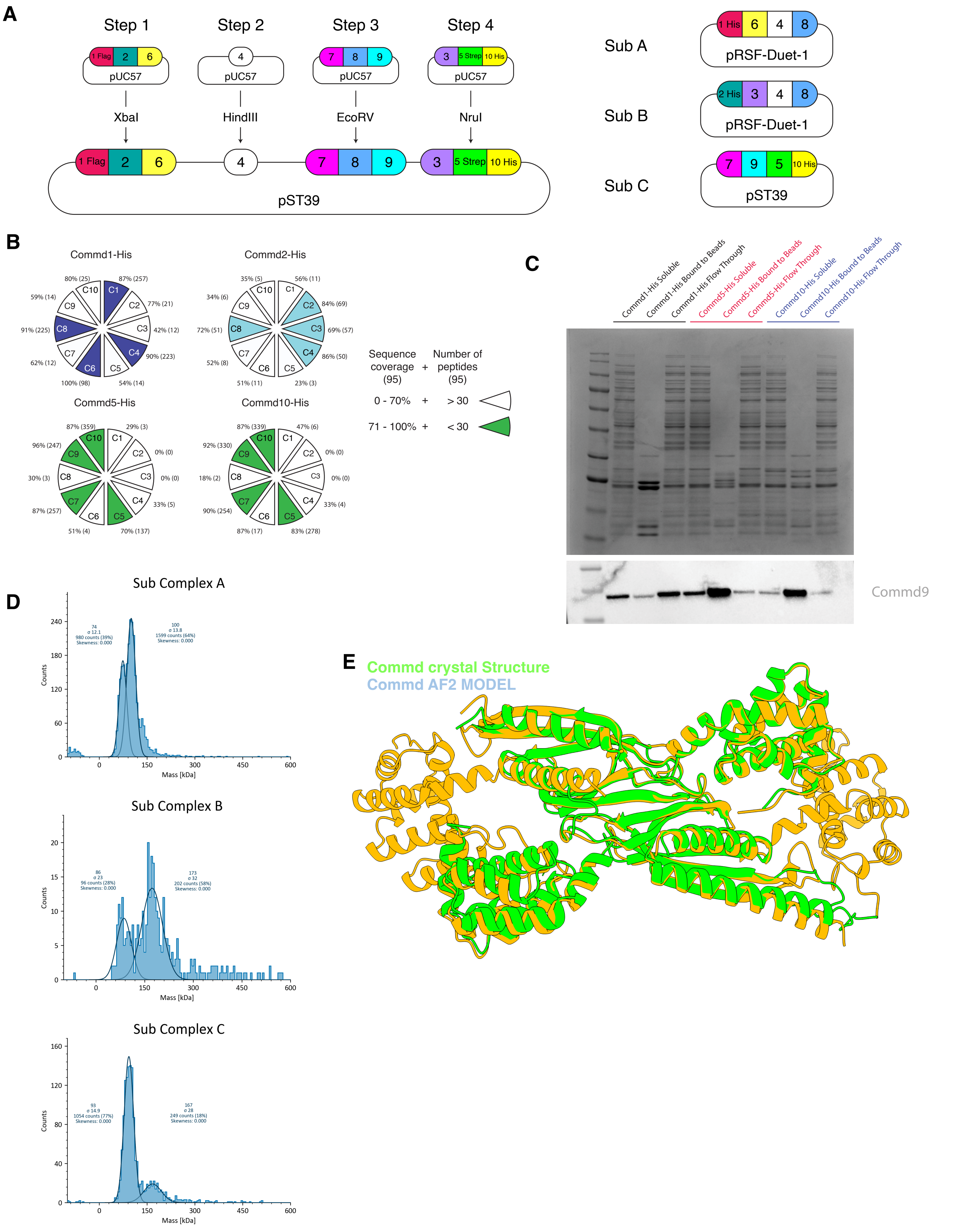

Expression, purification, and characterisation of recombinant COMMD complexes in E. coli.
(A) Design of co-expression vector for isolation of COMMD complexes from E. coli. Left gene cassettes were ordered from Gene Universal in pUC57 vectors and sequentially cloned in a pST39 vector for simultaneous expression. This vector included 3 proteins with affinity tags COMMD1-FLAG, COMMD5-StrepII, and COMMD10-His. From this initial vector, 4 vectors were generated that contained only a single His tag on COMMD1, COMMD2, COMMD5 and COMMD10. Right shows pRSF-Duet-1 vectors that express the individual COMMD subcomplexes. (B) Purification of protein using different His tag locations lead to the proteomic identification of subcomplex A, subcomplex B, and subcomplex C. (C) This result was also confirmed by western blot with only COMMD5 and COMMD10 His able to enrich for COMMD9. (D) Mass photometry confirmed that the masses of subcomplexes generally agreed with the expected values (A, 74 kDa (74); B, 86 kDa (88); C, 93 kDa (92)). (E) Comparison of the AlphaFold 2 (AF2) prediction shows good agreement with the 3.3 Å crystal structure of subcomplex C.

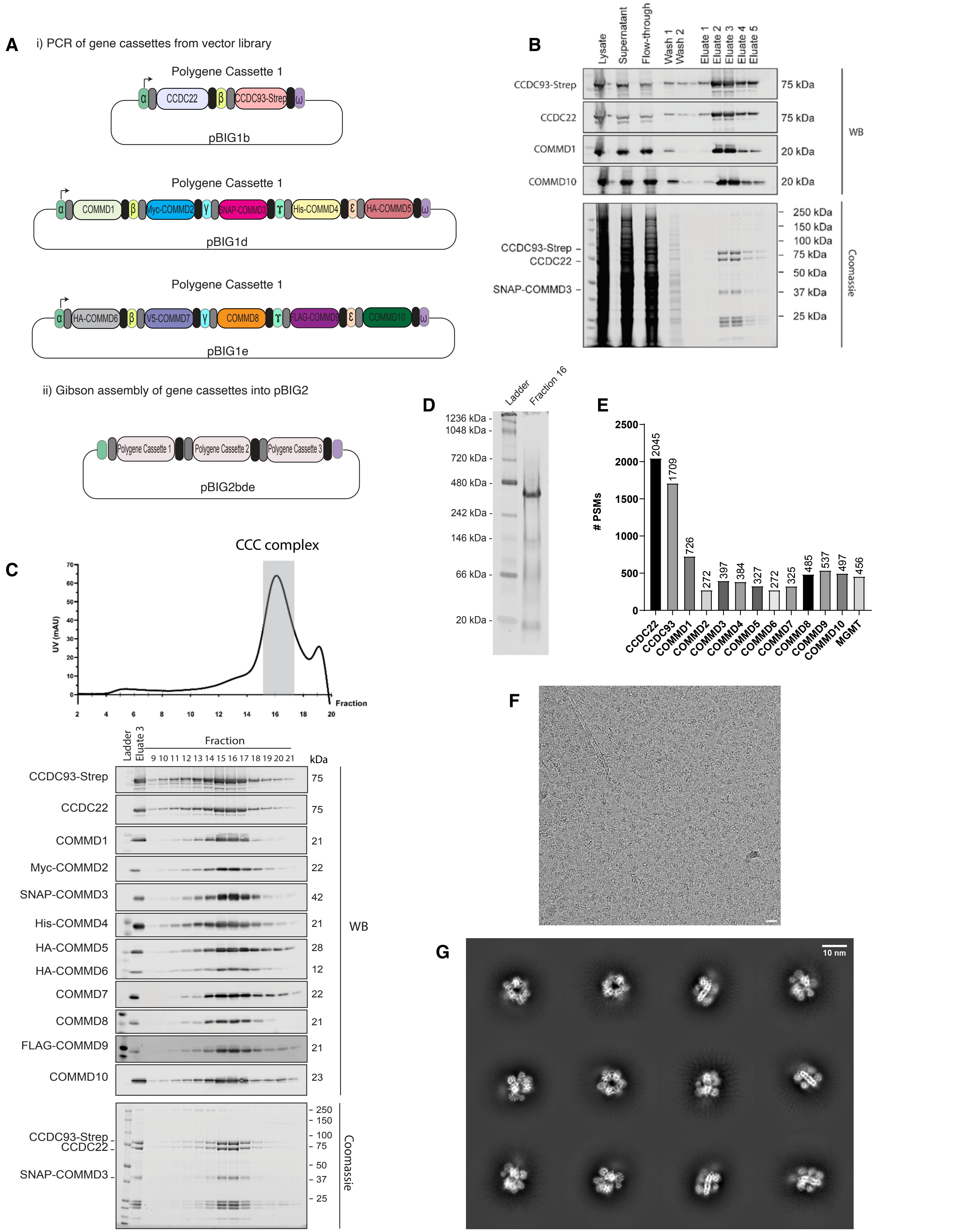

Expression, purification, and characterisation of recombinant CCC complexes in insect cells.
(A) Schematic of the engineering of pbiGbde-CCC from Gibson assembly of individual polygene cassettes. (B) Coomassie and western analysis of strep-actin affinity purified human CCC complex from baculovirus infected insect cells. (C) Gel filtration of the affinity purified CCC complex and western analysis of the purified human CCC complex. (D) Native PAGE of purified CCC complex. (E). Mass spec analysis of purified CCC complex establishing the presence of all twelve proteins. Data is from one of two independent experiments. (F) Representative motion-corrected 10 Å lowpass filtered micrograph of vitrified, cross-linked CCC complex on graphene oxide coated 1.2/1.3 Quantifoil™ grids. Scale bar represents 20 nm. (G) Single-particle 2D cryo-EM classes, generated in cryoSPARC, revealing the globular, ring-like structure of the CCC complex. Scale bar represents 10 nm.

Cryosparc data processing flow chart

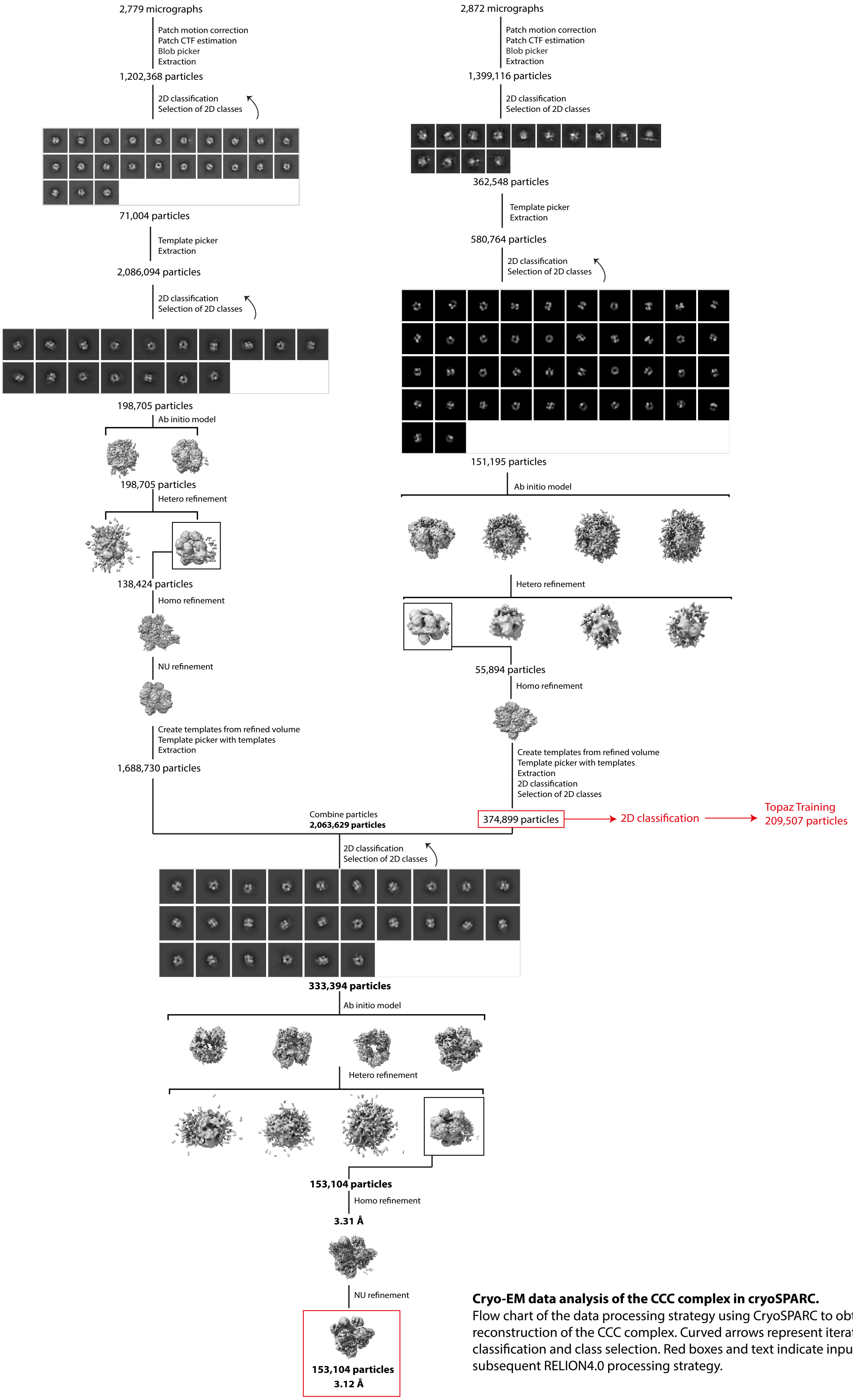

Cryo-EM data analysis of the CCC complex in cryoSPARC.
Flow chart of the data processing strategy using CryoSPARC to obtain a 3.12 Å reconstruction of the CCC complex. Curved arrows represent iterative rounds of classification and class selection. Red boxes and text indicate inputs into the subsequent RELION4.0 processing strategy.

RELION 4.0 data processing flow chart

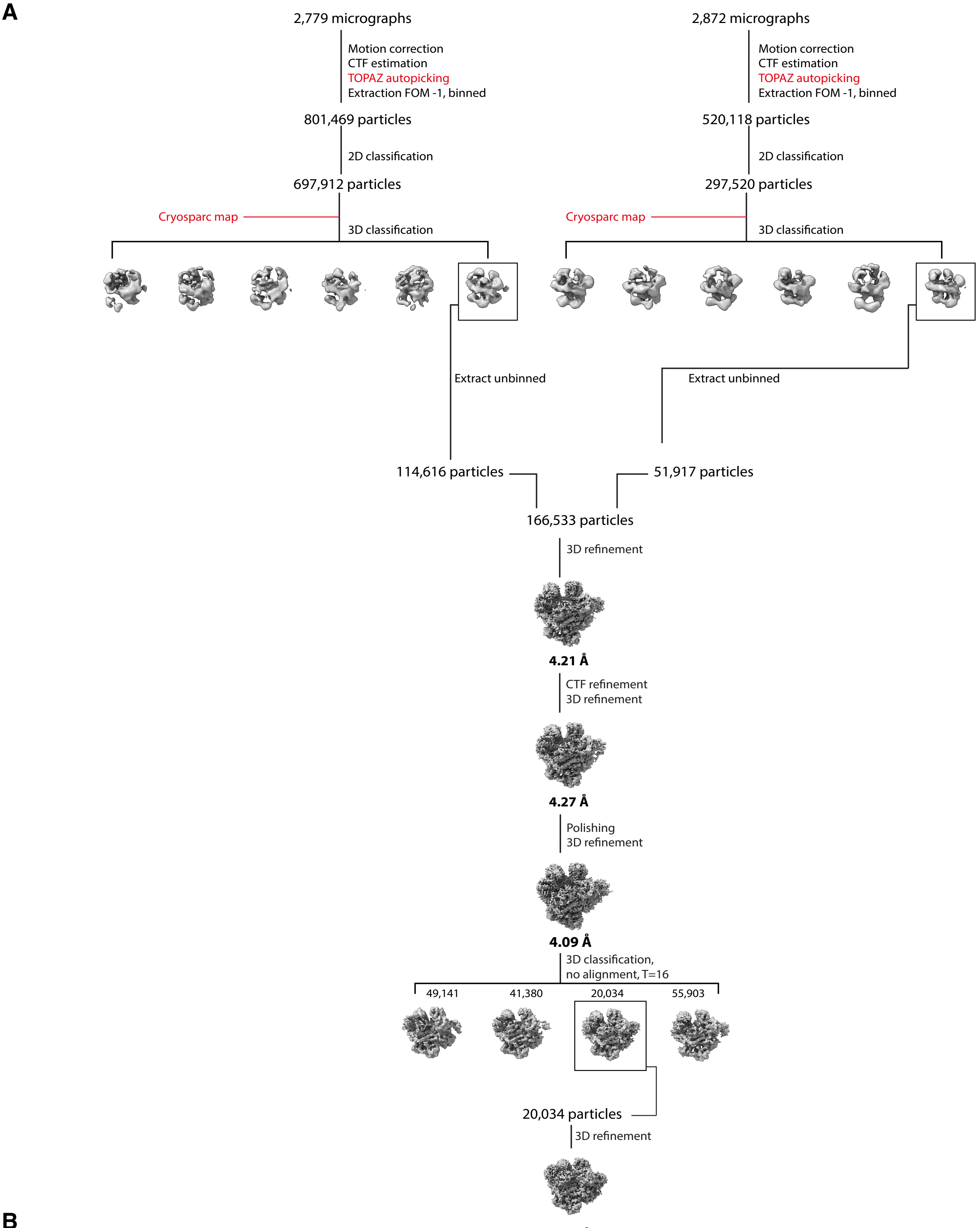

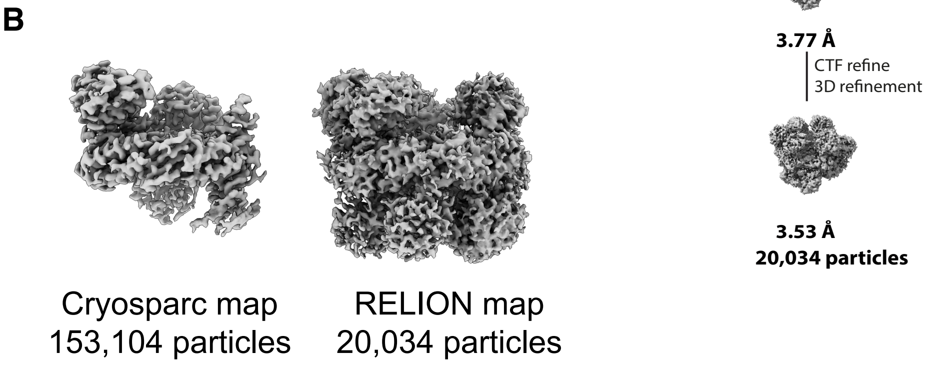

Cryo-EM data analysis of the CCC complex in RELION4.0. (A) Flow chart of the data processing strategy using RELION4.0 to obtain a 3.53 Å reconstruction of the CCC complex. Red text indicates inputs from processing the data in CryoSPARC. (B) Representative snapshots of cryoSPARC and RELION reconstructions from ChimeraX. Whilst the cryoSPARC reconstruction has a core of higher resolution, the RELION reconstruction contains better resolved densities for flexible regions.

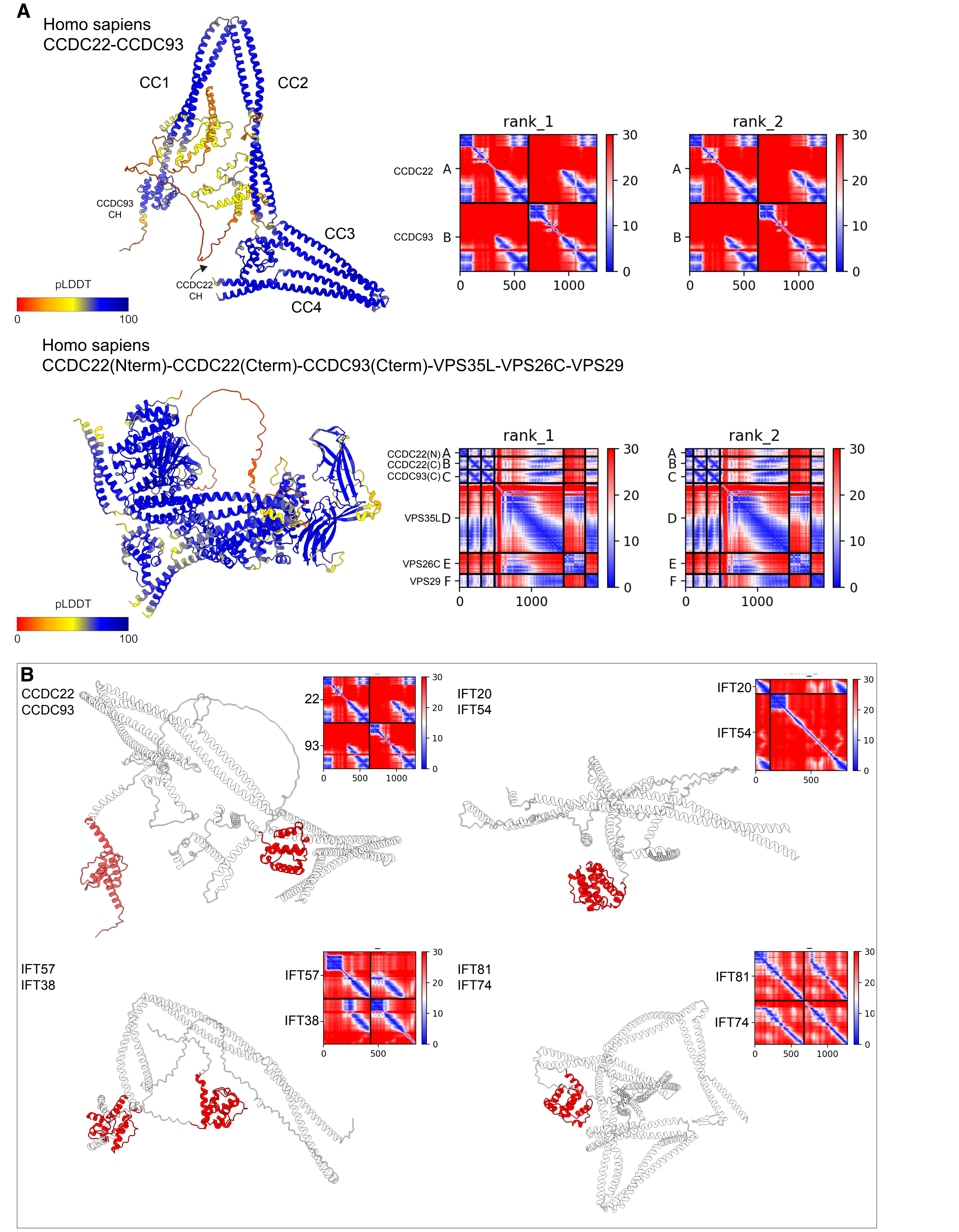

CCDC22 and CCDC93 heterodimeric coiled-coil share similarity with various IFT subunits of the intraflagellar transport machinery.
(A) AlphaFold2 models of full length CCDC22 and CCDC93 (top) as well as truncated CCDC22 and CCDC93 in complex with Retriever (bottom) show the formation of a highly conserved coiled-coil structure able to interact with Retriever. (B) Highlights the similarity between CCDC22 and CCDC93 and IFT proteins. Each of these proteins contain a calponin homology (CH) domain (red) and an extended coiled-coil region. Previously CH domains have been shown to bind both actin and microtubules which could suggest a similar function for the CH domains of CCDC22 and CCDC93.

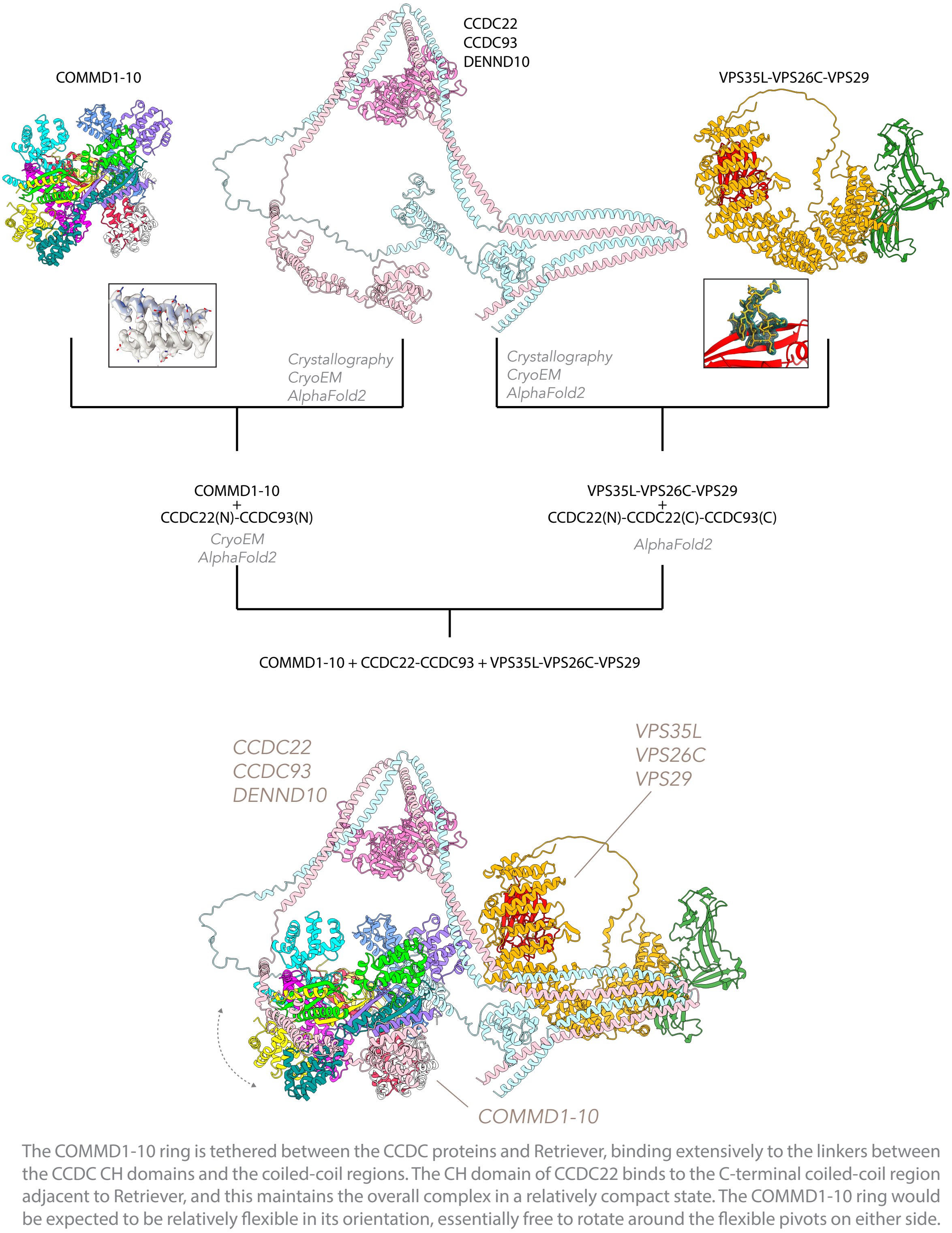

Workflow for assembling the complete Commander complex combining experimental structures and AlphaFold2 modelling.
A flow chart showing the various methodologies used to assemble the Commander complex. We used a combination of AlphaFold2 modelling, X-ray crystallographic structures and cryoEM to establish models of Retriever and the CCC complex. Using AlphaFold2 it was possible to model CCDC22 and CCDC93, which act as a bridge between the two complexes. The final model reveals that the COMMD1-10 ring is tethered between the CCDC proteins and Retriever, binding extensively to the linkers between the CCDC CH domains and the coiled-coil regions. The CH domain of CCDC22 binds to the C-terminal coiled-coil region adjacent to Retriever, and this maintains the overall complex in a relatively compact state. The COMMD1-10 ring would be expected to be relatively flexible in its orientation, essentially free to rotate around the flexible pivots on either side. As outlined in the text, all key interfaces have been experimentally validated by mutagenesis, co-immunoprecipitation and cellular rescue studies.
